# Risk of Adverse Outcomes for Older People with Dementia Prescribed Antipsychotic Medication: A Population Based e-Cohort Study

**DOI:** 10.1007/s40120-016-0060-6

**Published:** 2017-01-04

**Authors:** Michael Dennis, Laura Shine, Ann John, Amanda Marchant, Joanna McGregor, Ronan A. Lyons, Sinead Brophy

**Affiliations:** 1 Farr Institute of Health Informatics Research, Swansea University Medical School, Swansea, Wales UK; 2 Cwm Taf Health Board, Port Talbot, Wales UK

**Keywords:** Antipsychotic medication, Dementia, Mortality, Older people

## Abstract

**Introduction:**

Over recent years there has been growing evidence of increased risk of mortality associated with antipsychotic use in older people with dementia. Although this concern combined with limited evidence of efficacy has informed guidelines restricting antipsychotic prescription in this population, the use of antipsycotics remains common. Many published studies only report short-term outcomes, are restricted to examining mortality and stroke risk or have other limitations. The aim of this study was to assess adverse outcomes associated with the use of antipsychotics in older people living with dementia in Wales (UK).

**Methods:**

This was a retrospective study of a population-based dementia cohort using the Welsh Secure Anonymised Information Linkage databank. The prior event rate ratio (PERR) was used to estimate the influence of exposure to antipsychotic medication on acute cardiac events, venous thromboembolism, stroke and hip fracture, and adjusted Cox proportional hazard models were used to compare all-cause mortality.

**Results:**

A total of 10,339 people aged ≥65 years were identified with newly diagnosed dementia. After excluding those who did not meet the inclusion criteria, 9674 people remained in the main cohort of whom 3735 were exposed to antipsychotic medication. An increased risk of a venous thromboembolic episode [PERR 1.95, 95% confidence interval (CI) 1.83–2.0], stroke (PERR 1.41, 95% CI 1.4–1.46) and hip fracture (PERR 1.62, 95% CI 1.59–1.65) was associated with antipsychotic use. However, there was no long-term increased mortality in people exposed to antipsychotics (adjusted hazard ratio 1.06, 95% CI 0.99–1.13).

**Conclusions:**

The increase in adverse medical events supports guidelines restricting antipsychotic use in this population.

**Electronic supplementary material:**

The online version of this article (doi:10.1007/s40120-016-0060-6) contains supplementary material, which is available to authorized users.

## Introduction

There are currently approximately 700,000 people in the UK with dementia [1]. As well as cognitive disturbances, people living with dementia also commonly develop behavioural and psychological symptoms (BPSD). Clinical guidelines for BPSD emphasise managing aetiological factors, and the use of behavioural and psychological interventions [2]. However, antipsychotic medications have often been prescribed for BPSD despite evidence of limited clinical improvement [3–8]. Over the past decade concerns have been raised over the prescribing of antipsychotics in people living with dementia, with the UK Committee for the Safety of Medicines (CSM) and the U.S. Food and Drug Administration (FDA) issuing statements to this effect in 2004 [9] and 2005 [10], respectively. These concerns are supported by meta-analyses of randomised controlled trials (RCTs) [5, 7, 11] and a large U.S. cohort study [12]. Reports and guidelines for clinicians have subsequently expressed the need for extreme caution in prescribing antipsychotic medication to people living with dementia [1, 2, 13, 14]. Despite this, antipsychotics are still commonly used in clinical practice in the UK in this patient group [15, 16]. The newer, ‘atypical’ antipsychotic drugs appear to carry lower risk than conventional (typical) antipsychotic agents and the highest risk is in the first 1–3 months of treatment [17–19].

There are, however, issues concerning the findings of increased risk of mortality and other adverse health outcomes in people with dementia prescribed antipsychotic medication. Many of the published studies only report short-term adverse outcomes, the trials are often from highly selected populations, control group selection in observational studies is often inadequate and increased mortality is not a universal finding [20–23].

The objective of our study was to perform a large population-based retrospective electronic-cohort study of older people living with dementia using linked routine data, examining the relationship between antipsychotics and serious adverse outcomes.

## Method

### Data Source

The retrospective cohort of older people with dementia for this study was drawn from the Secure Anonymised Information Linkage (SAIL) databank based at the Health Information Research Unit (HIRU), Swansea University. SAIL brings together and links the widest possible range of anonymised routinely collected person-based data held in health and social care datasets in Wales (UK). A large amount of preliminary work on anonymisation methodologies to protect privacy was undertaken to create the SAIL system [24, 25]. SAIL uses a split-file approach to anonymisation to overcome confidentiality and disclosure issues. Within each routine dataset held in the SAIL databank, anonymised individuals are assigned a unique linking field or ALF (Anonymised Linking Field). These are generated by the NHS Wales Information Service using the Matching Algorithm for Consistent Results in Anonymised Linkage with 99.85% accuracy [25]. ALFs are further encrypted within SAIL and used to link de-identified individuals across multiple datasets. The SAIL system represents a valuable resource for health-related research whilst complying with the requirements of data protection legislation and confidentiality guidelines. For a more detailed description of SAIL see Jones et al. [26].

The particular datasets within the SAIL databank utilised for this project were:

- National Health Service (NHS) Administrative Register, which is a register of all individuals registered with a Welsh general practitioner (GP) or who have ever had contact with the NHS;
- General Practice Primary Care attendance and clinical information database for all general practice interactions including symptoms, investigations, diagnoses including co-morbidities, prescribed medication and referrals to secondary and tertiary care. The GPD database covered approximately 40% of the population of Wales at the time of the study;
- Cause of death (Office of National Statistics Public Health Mortality files);
- Patient Episode Dataset for Wales (PEDW), which includes demographic and clinical data on all inpatient and day-case admissions in NHS Wales hospitals and all Welsh residents treated in other UK countries;
- Outpatient Dataset, a minimum dataset listing all outpatient appointments, including mental health services in NHS Wales hospitals.

### Study Cohort

The SAIL databank was interrogated using structured query language (DB2 SQL) to create a cohort of people registered to a SAIL supplying GP practice and aged ≥57 years at the onset of the study period (1 January 2003). From this group, those with a new diagnosis of dementia during the study period (1 January 2003 to 31 December 2011) and aged ≥65 years at diagnosis were identified. The diagnosis of dementia was taken from GP computer records using NHS Read browser terminology. Read codes are a coded thesaurus of clinical terms which have been used in the NHS since 1985 and are still widely used in the primary care sector. They are the standard clinical terminology by which clinicians can record patient findings and procedures. The Read codes used to identify participants for the cohort were determined by using READ Version 2 (5-byte). This database was manually reviewed and searched by the researchers to identify all the possible codes that may have been used to record a diagnosis of dementia (Appendix). To ensure the list of codes was complete as possible, we also checked our list against codes for dementia used to identify cases for the Quality and Outcomes Framework (QOF READ codes v27 for 2013/2014 [27]) and the updated Charlson Index [28]. Patients entered the cohort at the date of diagnosis of dementia and left on the date of death, date of leaving a SAIL supplying a GP or the study end, whichever was the sooner.

Exclusions from the cohort included people who had a prior diagnosis of schizophrenia and bipolar affective disorder (functional psychosis) and individuals diagnosed with cancer within one year date of death. These individuals were again identified in a similar manner from the GP Read Coding [Electronic Supplementary Material (ESM) Tables S1–3]. The exclusion of people with functional psychosis was due to the fact that the antipsychotic prescription was likely to be for the treatment of the underlying psychotic illness, and the exclusion of cancer patients was because antipsychotics are used commonly for a different clinical indication, namely nausea in the context of palliative care.

### Exposure to Antipsychotic and Other Psychotropic Medications

From linked and anonymised prescribing records we then determined whether participants in the cohort had received antipsychotic medication. From the GP database we collated all prescriptions of antipsychotic medications, again determined by NHS Read codes (ESM Table S20). The type of medication was further divided into conventional (typical) and newer atypical antipsychotics. Because of the debate concerning the status of sulpiride (historically conventional but structurally related to newer drugs), this drug was coded separately to allow inclusion in either group. We also recorded information on the prescription of other psychotropic medications, in particular hypnotics and anxiolytics (ESM Tables S21 and S22). Within these groups data on benzodiazepine prescriptions were reviewed (ESM Table S23).

With regard to the prescriptions the date of first prescription after dementia diagnosis was recorded.

**Fig. 1:**
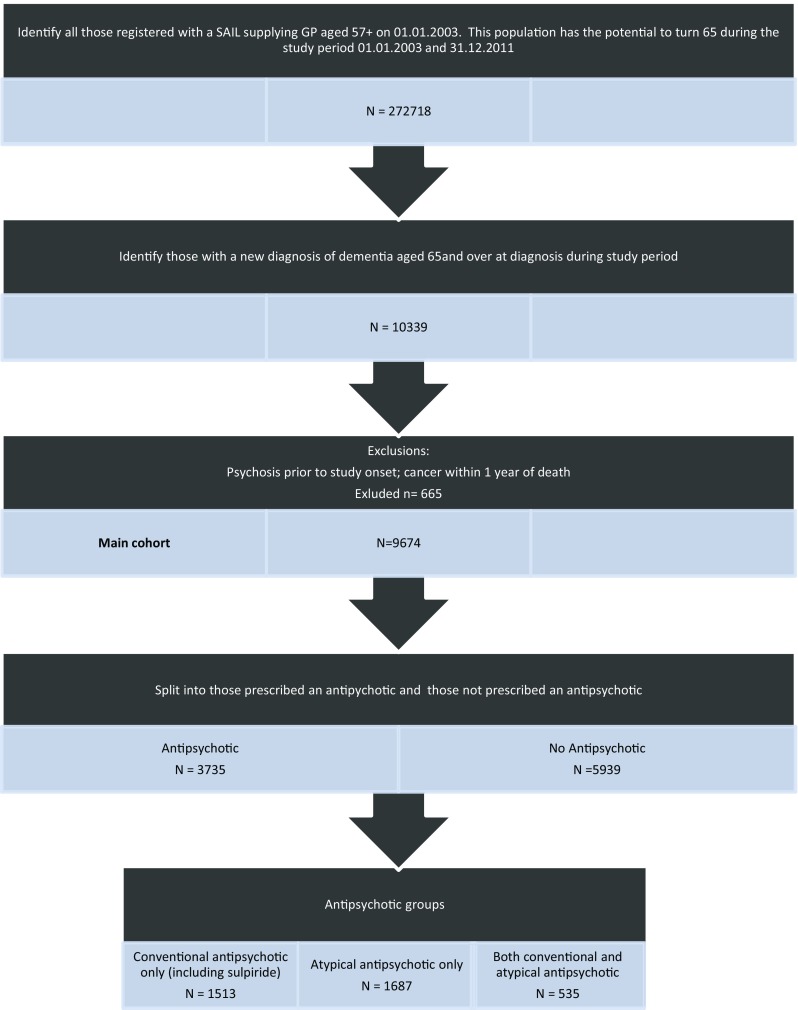
Assembly of study cohort of older people with dementia.* SAIL* Secure Anonymised Information Linkage database

We divided the cohort into two groups: one who had received a prescription for antipsychotic medications and a comparison who had not (Fig. 1).

### Clinical Characteristics, Co-morbidity and Outcomes

Data evaluated on all participants included in the cohort were gender and age at diagnosis of dementia. The following co-variants were also determined from the database information: cerebrovascular disease, ischaemic heart disease, parkinsonism, hip fracture, venous thromboembolic event, atrial fibrillation, epilepsy and diabetes. These co-variants were identified using NHS Read codes (ESM Tables S4–11) and their occurrence or non-occurrence prior to the onset of dementia was recorded.

The primary outcome was a serious adverse event, including death, cerebrovascular disease (episode of stroke or transient ischaemic attack), acute cardiac event, venous thromboembolic events [deep vein thrombosis (DVT) or pulmonary embolism (PE)] and hip fracture.

Participants were followed for the duration of the study, their time in the cohort, and length of follow-up dependent on their date of diagnosis of dementia. The outcomes were determined by using anonymously linked information on each participant in the cohort. Adverse outcomes were recorded from GP data based on NHS Read codes (ESM Supplementary Tables S12, S14, S16, S18). This information was combined with data from PEDW; diagnosis in our study was based on ICD-10 codes (ESM Supplementary Tables S13, S15, S17, S19). Data on death was obtained from the Office of National Statistics database and included information on date and cause of death. The Read and ICD10 codes were selected by manually searching the relevant classification lists. To ensure completeness of the included Read codes and ICD-10 we again checked our list against updated Charlson Index codes [28, 29].

### Statistical Analysis

Basic demographic characteristics and history of medical co-morbidities were compared between people with and without exposure to antipsychotic medication. Adjusted relative risks of death were calculated with Cox’s proportional hazard models. Risk was adjusted for age, gender and co-morbidities at dementia diagnosis (epilepsy, parkinsonism, atrial fibrillation, history of thromboembolism, diabetes, ischaemic heart disease, cerebrovascular disease, and previous hip fracture; Model 1). Further adjustment was made for use of hypnotics, anxiolytics and benzodiazepines (Model 2).

The prior event rate ratio (PERR) adjustment method was used to estimate the effects of exposure to antipsychotic medication on the likelihood of experiencing the adverse medical outcomes of acute cardiac event, venous thromboembolism, stroke and hip fracture. The time to first event over 12 months of follow-up (incidence) was calculated from the date of the first antipsychotic (follow-up for 12 months) and for the 12-month period prior to the date of the first antipsychotic. For those not given an antipsychotic, a random date was chosen to calculate time to first event prior and post this random date. The PERR is calculated as:

$$ {\text{PERR}} = \frac{{{\text{Rate}}\;{\text{ratio}}\;{\text{during}}\;{\text{post}}\;{\text{period}}}}{{{\text{Rate}}\;{\text{ratio}}\;{\text{during}}\;{\text{prior}}\;{\text{period}}}}. $$

Confidence intervals (CIs) were obtained by bootstrapping. The method assumes that the confounding effects are constant across the prior and post exposure periods and that there is no confounder by treatment interactions [30, 31]. The PERR cannot be applied for terminal events and so was used to examine acute cardiac events, venous thromboembolism (DVT and PE), stroke and hip fracture—but not mortality.

All data were analysed using STATA version 12 [32].

### Secondary Analyses

To explore whether particular types of dementia had different risk profiles we performed a sub-group analysis examining outcomes for people suffering from the main type of dementia, Alzheimer’s disease (AD). Secondary analyses also included a comparison of outcomes in people receiving newer atypical antipsychotics and those receiving conventional antipsychotic medication. Adjusted hazard ratios (HRs) were calculated for both models. Additionally, we examined mortality rates in 100-day blocks following initial antipsychotic prescription.

### Additional Sensitivity Analyses

We conducted two additional analyses to further examine mortality risk differences:

1. Excluding people with contact with specialist services in old age psychiatry. This was performed in the case that antipsychotic medication had been prescribed in a secondary care setting that would not have been identified in the primary care database. It is possible that antipsychotic medication could be prescribed by specialist services in an emergency situation or at the commencement of treatment in an out-patient consultation.
2. To determine whether the exclusion of people with a diagnosis of cancer within 1 year of death influenced the results, we repeated the main analysis by including these people.

### Compliance with Ethics Guidelines

This article does not contain any new studies with human or animal subjects performed by any of the authors. Ethical approval was granted from the HIRU Information Governance Review Panel, an independent body consisting of a range of government, regulatory and professional agencies which oversees study approvals in line with permissions already granted to the analysis of data in the SAIL databank [24, 25].

The results are reported in accordance with the STROBE checklist (http://www.epidem.com/).

## Results

### Cohort Characteristics

From 2003 until 2011 there were 272,718 people registered with a SAIL supplying GP in Wales who were or had the potential to turn 65 years of age during the study period. From these we identified 10,339 people with a new diagnosis of dementia aged ≥65 years at diagnosis. A total of 665 were excluded because of pre-existing functional psychosis or a diagnosis of cancer within 1 year of death, leaving a total of 9674 people with dementia forming the main cohort. Within this main cohort, 3735 were exposed to antipsychotic medication, and 5939 did not receive any antipsychotics from their GP following their dementia diagnosis. Figure 1 provides a summary of the dementia e-cohort assembly process.

The demographic characteristics and baseline medical co-morbidities of older people with dementia exposed and not exposed to antipsychotic medications were remarkably similar (Table 1). The exceptions to this were a small difference in gender, with males more likely to receive antipsychotics, and a higher proportion of people in the non-exposed group suffering from diabetes before dementia diagnosis. There were high rates of baseline co-morbidity, particularly ischaemic heart disease and cerebrovascular disease. The median follow-up period (until death, leaving a SAIL supplying GP, or end of study period) was 1.8 [interquartile range (IQR) 0.8–3.3] years in the main dementia cohort, 2.2 (IQR 1.1–3.8) years in the group exposed to antipsychotic medication and 1.5 (IQR 0.6–3.0) years in those not exposed to antipsychotics.

**Table 1:** General characteristics of dementia cohort by antipsychotic use

General characteristics of study cohort	Exposed to antipsychotic medication (*n* = 3735)	Not exposed to antipsychotic medication (*n* = 5939)	% Difference (95% CI)
Female	66.2% (2474)	67.8% (4025)	1.5* (0–3.4)
Mean age at diagnosis of dementia (years)	82.4 (6.8)	82.5 (6.8)	0.1 (−0.1 to 0.4)
History of			
Epilepsy	2.7% (99)	2.3% (138)	0.3 (−0.3 to 1.0)
Parkinsonism	4.0% (149)	3.7% (222)	0.25 (−0.5 to 1.1)
Atrial fibrillation	14.6% (546)	15.4% (914)	0.7 (−2.2 to 0.7)
Venous thromboembolism	5.8% (217)	5.5% (329)	0.27 (−0.6 to 1.2)
Diabetes	13.7% (511)	15.8% (941)	2.2* (0.7–3.6)
Ischaemic heart disease	23.9% (893)	23.2% (1377)	0.7 (−1.0 to 2.5)
Cerebrovascular disease	22.3% (832)	22.5% (1338)	0.25 (−2.0 to 1.5)
Hip fracture	6.5% (243)	6.1% (364)	0.4 (−0.6 to 1.4)

* Significant difference between the group exposed to antipsychotic medication and the group not exposed

Values in table are presented as the percentage with the number (of people) in parenthesis, with the exception of Mean age at diagnosis which is presented as the mean age with the standard deviation in parenthesis

### Antipsychotic Exposure, Mortality and Other Main Outcomes

Older people with dementia exposed to antipsychotic medication did not have an increased risk of mortality compared to non-exposed people when adjustment was made for age at dementia diagnosis, gender or baseline co-morbidity (Model 1) or after adjusting for age at dementia diagnosis, gender, baseline co-morbidity and exposure to anxiolytic and/or hypnotic medication (Model 2) (Table 2). However, PERR analysis showed a greater likelihood of increased venous thromboembolism (DVT and PE), stroke and hip fracture, but not acute cardiac event, in those receiving antipsychotic medication (Table 3).

**Table 2:** Adjusted mortality hazard ratios by exposure to antipsychotic medication

Time to	Exposed to antipsychotic medication			Not exposed to antipsychotic medication			Adjusted HR Model 1 (95% CI)^a^	Adjusted HR Model 2 (95% CI)^b^
	Events	Follow-up years	Rate per 100 person-years (95% CI)	Events	Follow-up years	Rate per 100 person-years (95% CI)		
All older people with all-cause dementia (*n* = 9674)	2342	100.9	23.2 (22.2–24.2)	2777	121.9	22.8 (22.0–23.6)	1.0 (0.96–1.09)	1.06 (0.99–1.13)
Older people with Alzheimer’s disease (*n* = 6996)	1689	74.0	22.8 (22.2–23.9)	2006	90.2	22.2 (21.2–23.2)	1.02 (0.96–1.09)	1.06 (0.99–1.14)
Older people with all-cause dementia known to primary care only (*n* = 5089)	1122	37.7	29.8 (28.1–31.6)	1834	61.0	30.0 (28.7–31.5)	1.0 (0.91–1.10)	1.03 (0.96–1.10)

*CI* Confidence interval,* HR* hazard ratio

^a^Model 1 = adjusted for history of medical co-morbidities as listed in Table 1, age at dementia diagnosis and gender

^b^Model 2 = adjusted for history of medical co-morbidities as listed in Table 1, age at dementia diagnosis, gender, and exposure to anxiolytic and/or hypnotic medication

**Table 3:** Adverse event hazard ratio and adjusted prior event rate ratios by exposure to antipsychotic medication

Adverse event	Exposed to antipsychotic	Not exposed to antipsychotic	Hazard ratio	PERR (95% CI)
**Acute cardiac event**				
All-cause dementia				
Before				
*N* per 1000	5.9 (22/3721)	7.1 (42/5908)	0.83	
95% CI	3.9–9.0	5.2–9.6		
After				
*N* per 1000	12.4 (46/3720)	15.1 (89/5908)	0.82	0.98 (1.0–1.0)
95% CI	9.2–16.6	12.2–18.6		
Alzheimer’s				
Before				
*N* per 1000	5.2 (14/2690)	9.6 (41/4270)	0.54	
95% CI	3.1–8.8	7.0–13.0		
After				
*N* per 1000	11.1 (30/2680)	12.4 (53/4260)	0.89	1.65* (1.59–1.86)
95% CI	7.8–16.0	9.5–16.2		
Known to primary care only				
Before				
*N* per 1000	5.5 (9/1642)	8.5 (29/3419)	0.64	
95% CI	2.9–10.5	5.8–12.2		
After				
*N* per 1000	14.1 (23/1634)	12.9 (44/3408)	1.08	1.68* (1.05–1.78)
5% CI	9.3-21.1	9.6-17.3		
**Venous thromboembolism**				
All-cause dementia				
Before				
*N* per 1000	6.4 (24/3717)	6.7 (40/5909)	0.96	
95% CI	4.3–9.6	5.0–9.2		
After				
*N* per 1000	13.0 (48/3705)	6.9 (41/5914)	1.88	1.95* (1.83–2.0)
95% CI	9.8–17.2	5.1–9.4		
Alzheimer’s				
Before				
*N* per 1000	7.0 (19/2684)	6.5 (28/4276)	1.0	
95% CI	4.5-11.0	5.0-9.2		
After				
*N* per 1000	14.2 (38/2674)	7.7 (33/4274)	1.8	1.80* (1.67–1.89)
95% CI	10.3-19.5	5.4-10.8		
Known to primary care only				
Before				
*N* per 1000	6.7 (11/1639)	9.6 (33/3416)	0.7	
95% CI	0.3–12.1	5.0–9.2		
After				
*N* per 1000	14.1 (23/1632)	7.6 (26/3422)	1.85	2.66* (2.41–2.78)
95% CI	9.3–21.2	5.2–11.1		
**Stroke**				
All-cause dementia				
Before				
*N* per 1000	35.0 (128/3652)	37.8 (219/5789)	0.92	
95% CI	29.4–41.6	33.1–43.1		
After				
*N* per 1000	65.8 (236/3587)	50.8 (292/5749)	1.3	1.41* (1.40–1.46)
95% CI	57.9–74.7	45.2–57.0		
Alzheimer’s				
Before				
*N* per 1000	25.6 (68/2653)	34.0 (143/4207)	0.75	
95% CI	20.2–32.4	28.8–40.0		
After				
*N* per 1000	61.1 (159/2601)	39.6 (166/4190)	1.54	2.06* (1.97–2.13)
95% CI	52.3–71.4	34.0–46.1		
Known to primary care only				
Before				
*N* per 1000	41.9 (67/1599)	50.2 (167/3327)	0.83	
95% CI	32.9–53.2	43.1–58.4		
After				
*N* per 1000	70.2 (111/1581)	48.6 (162/3330)	1.44	1.73* (1.66–1.75)
95% CI	58.2–84.5	41.7–56.7		
**Hip fracture**				
All-cause dementia				
Before				
*N* per 1000	24.4 (90/3679)	24.5 (143/5842)	1.0	
95% CI	19.9-30.1	20.8-28.8		
After				
*N* per 1000	55 (200/3617)	34.2 (199/5817)	1.61	1.62* (1.59–1.65)
95% CI	47.9–63.2	29.8-39.3		
Alzheimer’s				
Before				
*N* per 1000	27.1 (72/2653)	25.3 (107/4226)	1.07	
95% CI	21.5–34.2	20.9–30.6		
After				
*N* per 1000	59.0 (154/2608)	33.4 (141/4213)	1.76	1.65* (1.61–1.71)
95% CI	50.4–69.1	28.3–39.4		
Known to primary care only				
Before				
*N* per 1000	25.2 (41/1623)	22.4 (76/3390)	1.13	
95% CI	18.5–34.3	17.9–28.1		
After				
*N* per 1000	57.1 (91/1593)	32.3 (109/3369)	1.76	1.57* (1.52–1.65)
95% CI	46.5–70.1	26.8–39.0		

* Significantly higher likelihood of acute cardiac event, venous thromboembolism, stroke and hip fracture in those receiving antipsychotic medication

*PERR* Prior event rate ratio

### Secondary Analyses

There were 6996 people with a diagnosis of Alzheimer-type dementia, representing 72% of those within the main dementia cohort. Vascular dementia was the other main dementia subtype (*n* = 2333; 24%), followed by Lewy Body dementia (*n* = 139, 1.4%) and mixed dementia (*n* = 132, 1.4%). There were no significant differences in the proportions of people in different dementia subtype groups receiving antipsychotic medication (Chi-square 2.66(4), two-tailed *P* = 0.62). In both models of adjustment, people with AD exposed to antipsychotic drugs had no higher mortality risk than non-exposed individuals (Table 2). However, PERR analysis showed a greater likelihood of acute cardiac event, venous thromboembolism, stroke and hip fracture in those receiving antipsychotic medication (Table 3).

There were 3735 (39%) people exposed to antipsychotic medication in the main dementia cohort, of whom 1687 received an atypical antipsychotic medication, 1495 receive a conventional antipsychotic drug, 535 were exposed to both an atypical and conventional antipsychotic and 18 were exposed to sulpiride alone. The adjusted mortality HRs indicated no significant differences between those receiving conventional and atypical antipsychotics when people exposed to both were excluded (Model 1: adjusted HR 0.95, 95% CI 0.87–1.03; Model 2: adjusted HR 0.94, 95% CI 0.86–1.03). In this analysis, sulpiride was included as a conventional antipsychotic, but there was also no significant difference if sulpiride was classified as an atypical one. Interestingly, people with dementia receiving atypical antipsychotics had a higher risk of acute cardiac events following drug exposure than those receiving conventional antipsychotics (PERR 3.3, 95% CI 3.0–5.1), although the rates of pre-exposure were lower. Stroke risk was marginally lower in patients receiving atypical compared to conventional antipsychotics (PERR 0.82, 95% CI 0.8–0.9).

When we examined mortality rates for 100-day blocks following the commencement of antipsychotic medication, the rate was highest in the first 100 days—11.5 (95% CI 10.4–12.7) per 100 people per 100 days. This was a significantly higher rate than that for the 301- to 400-day block (8.7, 95% CI 7.6–10.1). The rates for the intervening periods, namely 101–200 days and 201–300 days, were 9.4 (95% CI 8.4–10.7) and 9.3 (95% CI 8.2–10.5), respectively.

### Sensitivity Analyses

When we excluded patients with a known contact with secondary care specialist mental health services for older people (outpatient and community team), the main cohort was reduced substantially from 9674 to 5089 people with dementia. Adjusted mortality HRs (both Model 1 and 2) showed no differences between those exposed and not exposed to antipsychotic medication (Table 2). The PERR analysis showed a raised risk of acute cardiac event, venous thromboembolism, stroke and hip fracture for people with dementia receiving antipsychotics compared to non-exposed patients (Table 3).

When we included people with cancer within 1 year of death in the dementia cohort, the adjusted mortality HR was still non-significant (Model 1: HR 0.97, 95% CI 0.92–1.02).

## Discussion

### Summary of Main Findings

The most important finding in our study was the increased likelihood of venous thromboembolism, stroke and hip fracture in older people living with dementia who were exposed to antipsychotic medication. The findings for people living with AD mirrored those for people living all-cause dementia, but the former had an additional increased risk of an acute cardiac event.

Surprisingly, there was no overall increased risk of death for older people living with dementia exposed to antipsychotic medication compared to non-exposed individuals although the mortality was comparatively high during the first 100 days of treatment with an antipsychotic and subsided thereafter. The absence of increased long-term mortality risk needs to be viewed with caution in view of a number of methodological limitations outlined below, including the absence of data on the duration of treatment. Interestingly, there was no difference in the risk of death between people receiving atypical and conventional antipsychotics.

The main study was conducted on 9674 older people (aged 65 years and over) with dementia in the region of Wales in the UK; this would include people living in their own homes as well as in residential and nursing homes.

### Results in Context

Our finding of a comparatively high mortality in the first 100 days after the initiation of antipsychotic treatment in older people with dementia is consistent with the body of literature describing the short-term increased mortality associated with antipsychotic use in dementia [11, 12, 33]. However, the absence of any overall long-term increased risk of mortality associated with exposure to antipsychotic medication requires a more detailed consideration. The first factor is that other studies which have examined longer-term effects of antipsychotics have similarly found no increased risk [22, 23]; in particular, Lopez et al. [22] showed that it was the presence of BPSD that was associated with the increased risk of mortality. Secondly, our cohort study occurred later than previously published studies, and most of the cohort period is after the publication of the CSM [9] and FDA [10] guidances; this may have resulted in more careful prescribing [34, 35], although the rates of ischaemic heart disease were similar in both exposed and non-exposed groups. Thirdly, our cohort has a relatively high mean age and is population based rather than a selected sample as utilised for some other cohort studies [12, 19]. Additionally, we have high rates of medical co-morbidity—almost double the rate of pre-existing cerebrovascular and significant cardiac disease compared to a large Canadian cohort study [33]. Clearly many of our cohort members would not have satisfied the stringent selection criteria for recruitment into a RCT for the treatment of BPSD in dementia. Also there may be a survival effect, as well as the possible role of improved aftercare compared to previous studies; there are comprehensive community services in Wales (UK) for older people with dementia, including in-reach to care homes [36]. One further explanation for a lack of overall increased mortality associated with antipsychotic use derives from studies that include measures of psychotic symptoms and agitation—when controlling for these factors the risk of mortality was not increased [20, 22], and this could be accounted for by the use of restraint in people not receiving medication [20]. However, there are also a number of methodological limitations that could account for the absence of increased long-term mortality associated with antipsychotics. In particular, the lack of duration of treatment with antipsychotics is an important consideration, as some people may have received a short-term prescription only. This could well be the case for an episode of delirium in the context of dementia. The finding that mortality was relatively high in the first 100 days following initiation of treatment and then subsided could be explained by a short duration of treatment. Secondly, stratification into groups of exposed and non-exposed to antipsychotic medication, respectively, for the Cox’s proportional hazard mortality analysis occurred following the diagnosis of dementia and was not time dependent (unlike for the PERR adjustment method); consequently, some people living with dementia for that particular analysis may be included in the exposed group despite having a significant period of non-exposure.

Besides examining mortality, other research into adverse outcomes in antipsychotic use in older people with dementia has focused on falls and stroke [13, 37]. Our findings of both an increased risk of hip fracture and stroke are consistent with the results of these previous studies. Our study utilised both hospital discharge data and primary care records to identify cerebrovascular adverse events, reducing the likelihood of more minor events in the community (such as transient ischaemic attack) or more terminal events managed in a care home setting being missed.

The risk of acute cardiac events as a consequence of antipsychotic use in older people with dementia has not been previously examined in detail [5, 6, 8], but there is an expectation of increased in risk based on drug trial data and the findings of electrocardiogram abnormalities in younger populations [38]. We had high levels of pre-existing ischaemic heart disease in our dementia cohort and found an increased risk of acute cardiac event in people living with AD who were exposed to antipsychotics, those living with dementia who received antipsychotics and were known only to primary care and those receiving atypical antipsychotic drugs compared to those receiving conventional ones. However, events were relatively few compared to other outcomes, in particular pre-treatment in those exposed to antipsychotic medication (especially atypicals). The higher risk associated with atypical compared to conventional antipsychotic medication may be explained by clinicians selecting one drug over another in ‘at risk’ patients; when prior events influence the probability of drug use, then bias may occur when using the PERR method [30].

An increased risk of venous thromboembolism associated with antipsychotic medication has previously been reported in an UK population-based nested case–control study, with older people at particular risk [39], and also in older people with dementia in a German nested case–control study [40]. Our study supports this finding in older people with dementia; inactivity-related venous stasis may not be the only mechanism involved—enhanced platelet aggregation and raised anticardiolipin antibodies may also be important factors [41].

Conventional antipsychotic agents have generally been found to be associated with a greater risk of mortality compared to newer atypical drugs [12, 17–19, 33, 42–45], but there are some exceptions [20, 22]. We found no difference between conventional and atypical antipsychotic drugs for mortality or any other adverse outcomes regardless of how sulpiride was categorised. This could be explained by some of the reasons previously outlined; in particular, the fact that our cohort is more recent. Studies based on Medicare and Medicaid primarily compared atypical drugs with haloperidol; the latter had often been prescribed in relatively high dosages for this frail client group [18, 42]. Other studies have failed to adequately consider the potential confounding effect of prescribing relatively high-dose typical antipsychotics for patients receiving palliative cancer care [19, 33, 43].

### Strengths and Limitations

This is the first large, long-term UK population based study of adverse outcomes associated with exposure to antipsychotics in older people with dementia. In utilising the SAIL databank we were able to assess baseline co-morbidity and identify adverse outcomes from both hospital in-patient episodes and primary care records, thereby reducing the possibility of missing adverse events. Additionally, we are able to include all dementia subtypes and not restrict our study to AD. Although the detection of dementia rate in Wales in 2015 was 43.4%, this estimated rate is based on figures utilising the basic Read codes used in the NHS QOF data [46]. Our study pre-dates the use of these codes for QOF in Wales, and one of the strengths of the study is the broader use of diagnostic codes to identify dementia. The dementia Read codes used in our study are similar to those used in research based upon the Clinical Practice Research Datalink in England; they have been found to have positive predictive value of 80–90% [47] and have high levels of agreement with Hospital Episode Statistics data [48]. Our case identification is therefore sufficiently reliable for a study of this nature, particularly as we were primarily interested in the effects of antipsychotic prescribing in patients known to have dementia in the primary care setting. Additionally, our cohort is generally from a later period than comparable studies—2003–2011 and predominantly after publication of the CSM and FDA guidances. Other strengths are our broad use of diagnostic codes, ability to control for effects of other psychotropic use and our careful exclusion criteria. Older people with pre-existing dementia are particularly vulnerable to experiencing concurrent delirium when physically unwell, and the lack of data on co-morbid delirium could be relevant for the Cox’s mortality HR analyses. Unfortunately, delirium is poorly recorded in general practice [49], and so it is difficult to control for this factor. However, we chose the adjusted PERR method for non-fatal adverse outcomes as it can reduce bias as a consequence of unmeasured confounding [30, 50]. Additionally, as we were examining antipsychotic prescribing in general practice and subsequent serious adverse outcomes in relation to conditions that frequently lead to hospitalisation, it is unlikely that co-morbid delirium at the time of index prescribing significantly influenced our findings.

There are limitations to the data available within the SAIL databank, namely the lack of information on individual drug frequency and the duration of prescription. The influence of these two factors has been discussed previously, as well as the limitations of the Cox proportional hazard analysis for mortality risk. Another deficiency is the absence of prescribing data for secondary care. However, in our sensitivity analyses we excluded patients known to older peoples’ mental health services, and the findings remained relatively consistent. There remains the possibility that a small number of people may have had a hospital in-patient episode when they were exposed to an antipsychotic for a short period, and this was not continued on discharge from hospital, or an antipsychotic could have been prescribed for BPSD before the dementia diagnosis was formally noted in the patient’s records. Another limitation associated with the use of linked routine data is the inability to consider the influence of degree of functional and cognitive impairment or the presence and severity of BPSD.

## Conclusion

Although there are only limited effects on behavioural and psychological symptoms with antipsychotic medications in older people with dementia, they continue to be used despite concerns over their safety [5]. Guidelines stress the importance of managing contributing medical co-morbidity, as well as the use of environmental, psychological and behavioural treatment strategies [2]. Although evidence for non-pharmacological interventions is increasing [51, 52], the availability and implementation of these interventions remain restricted by resources, and additionally the evidence for their efficacy still requires further evaluation [18].

Our study shows that there is a clear increased risk of adverse medical outcomes, in particular venous thromboembolism (DVT and PE), stroke and hip fracture in older people living with dementia who are exposed to antipsychotic medications. This risk supports recommendations restricting non-emergency antipsychotic medication use in people with dementia to those with psychosis or severe aggression when other non-pharmacological treatment strategies have failed [52–54].

### Electronic supplementary material

Below is the link to the electronic supplementary material.

Supplementary material 1 (DOCX 167 kb)

## Appendix 1

See Table 4.

**Table 4:** Read codes for dementia

Senile dementia	E00..
Senile/pre-senile dementia	E00..
Uncomplicated senile dementia	E000.
Pre-senile dementia	E001.
Uncomplicated pre-senile dementia	E0010
Pre-senile dementia with delirium	E0011
Pre-senile dementia with paranoia	E0012
Pre-senile dementia with depression	E0013
Pre-senile dementia NOS	E001z
Senile dementia with depressive or paranoid features	E002.
Senile dementia with paranoia	E0020
Senile dementia with depression	E0021
Senile dementia with depressive or paranoid features	E002z
Senile dementia with delirium	E003.
Arteriosclerotic dementia	E004.
Multi-infarct dementia	E004.
Uncomplicated arteriosclerotic dementia	E0040
Arteriosclerotic dementia with delirium	E0041
Arteriosclerotic dementia with paranoia	E0042
Arteriosclerotic dementia with depression	E0043
Arteriosclerotic dementia NOS	E004z
Other alcoholic dementia	E012.
Chronic alcoholic brain syndrome	E0120
Dementia in conditions EC	E041.
[X]Dementia in Alzheimer’s disease	Eu00.
[X]Dementia in Alzheimer’s disease with early onset	Eu000
[X]Pre-senile dementia, Alzheimer’s type	Eu000
[X]Primary degenerative dementia, Alzheimer’s type, pre-senile onset	Eu000
[X]Dementia in Alzheimer’s disease with late onset	Eu001
[X]Primary degenerative dementia of Alzheimer’s type, senile onset	Eu001
[X]Senile dementia, Alzheimer’s type	Eu001
[X]Dementia in Alzheimer’s disease, atypical or mixed type	Eu002
[X]Alzheimer’s dementia unspecified	Eu00z
[X]Dementia in Alzheimer’s disease, unspecified	Eu00z
[X]Arteriosclerotic dementia	Eu01.
[X]Vascular dementia	Eu01.
[X]Vascular dementia of acute onset	Eu010
[X]Multi-infarct dementia	Eu011
[X]Predominantly cortical dementia	Eu011
[X]Sub-cortical vascular dementia	Eu012
[X]Mixed cortical and sub-cortical vascular dementia	Eu013
[X]Other vascular dementia	Eu01y
[X]Vascular dementia, unspecified	Eu01z
[X]Dementia in other diseases classified elsewhere	Eu02.
[X]Dementia in Picks disease	Eu020
[X]Dementia in Creutzfeldt-Jacob disease	Eu021
[X]Dementia in Huntington’s disease	Eu022
[X]dementia in Parkinson’s disease	Eu023
[X]dementia in HIV disease (this may be a sensitive code)	Eu024
[X]Lewy body dementia	Eu025
[X]Dementia in other specified diseases classified elsewhere	Eu02y
[X]Primary degenerative dementia NOS	Eu02z
[X]Senile dementia NOS	Eu02z
[X]Senile dementia, depressed or paranoid type	Eu02z
[X]Unspecified dementia	Eu02z
[X]Delirium superimposed on dementia	Eu041
Alzheimer’s disease	F110.
Alzheimer’s disease with early onset	F1100
Alzheimer’s disease with late onset	F1101
Pick’s disease	F111.
Senile degeneration of brain	F112.
Lewy body disease	F116.
Cerebral degeneration in other disease	F11x
Cerebral degeneration due to alcohol	F11x0
Cerebral degeneration due to beriberi	F11x1
Cerebral degeneration due to cerebrovascular disease	F11x2
Cerebral degeneration due to myxodema	F11x5
Cerebral degeneration due to Vitamin B12 deficiency	F11x6
Cerebral degeneration due to Jacob-Creutzfeldt disease	F11x7
Cerebral degeneration due to multifocal leucoencephalopathy	F11x8
Cerebral degeneration due to Parkinson’s disease	F11x9
Cerebral degeneration due to other disease NOS	F11xz
[X]Other Alzheimer’s disease	Fyu30
