# Correction to: Risk of Adverse Outcomes for Older People with Dementia Prescribed Antipsychotic Medication: A Population Based e-Cohort Study

**DOI:** 10.1007/s40120-018-0093-0

**Published:** 2018-03-09

**Authors:** Michael Dennis, Laura Shine, Ann John, Amanda Marchant, Joanna McGregor, Ronan A. Lyons, Sinead Brophy

**Affiliations:** 1 Farr Institute of Health Informatics Research, Swansea University Medical School, Swansea, Wales UK; 2 Cwm Taf Health Board, Port Talbot, Wales UK

## Correction to: Neurol Ther (2017) 6:57–77 10.1007/s40120-016-0060-6

This article was originally published under a Creative Commons Attribution-NonCommercial 4.0 International License (CC BY-NC 4.0), but has now been made available under a Creative Commons Attribution 4.0 International License (http://creativecommons.org/licenses/by/4.0/), which permits unrestricted use, distribution, and reproduction in any medium, provided you give appropriate credit to the original author(s) and the source, provide a link to the Creative Commons license, and indicate if changes were made. The PDF and HTML versions of the paper have been modified accordingly.

