# Supplementary material for: Risk of Adverse Outcomes for Older People with Dementia Prescribed Antipsychotic Medication: A Population Based e-Cohort Study
Source: Neurol Ther. 2017 Jan 4;6(1):57–77. doi: 10.1007/s40120-016-0060-6 (PMC5447553; doi:10.1007/s40120-016-0060-6)
Supplement: Supplementary file 1 — Supplementary material 1 (DOCX 167 kb) [file 40120_2016_60_MOESM1_ESM.docx]

**Risk of adverse outcomes for older people with dementia prescribed antipsychotic medication**

**Supplementary Tables of**

**Read and ICD-10 codes**

**.**

Contents

[Table S 1 List of Read codes used to identify psychosis as an exclusion in SAIL 3](#_Toc393804828)

[Table S 2 List of Read codes used to identify and exclude cancer in SAIL 7](#_Toc393804829)

[Table S 3 List of ICD-10 codes used to identify and exclude cancer in SAIL 39](#_Toc393804830)

[Table S 4 List of Read codes used to identify cerebrovascular disease as a covariate in SAIL 40](#_Toc393804831)

[Table S 5 List of Read codes used to identify hip fracture as a covariate in SAIL 44](#_Toc393804832)

[Table S 6 List of Read codes used to identify ischaemic heart disease as a covariate in SAIL 46](#_Toc393804833)

[Table S 7 List of Read codes used to identify epilepsy as a covariate in SAIL 50](#_Toc393804834)

[Table S 8 List of Read codes used to identify parkinsonism as a covariate in SAIL 52](#_Toc393804835)

[Table S 9 List of Read codes used to identify atrial fibrillation as a covariate in SAIL 53](#_Toc393804836)

[Table S10 List of Read codes used to identify venous thromboembolic event as a covariate in SAIL 54](#_Toc393804837)

[Table S11 List of Read codes used to identify diabetes as a covariate in SAIL 56](#_Toc393804838)

[Table S12 List of Read codes used to identify cerebrovascular disease as an outcome in SAIL 62](#_Toc393804839)

[Table S13 List of ICD-10 codes used to identify cerebrovascular disease as an outcome in SAIL 64](#_Toc393804840)

[Table S14 List of Read codes used to identify hip fracture as an outcome in SAIL 66](#_Toc393804841)

[Table S15 List of ICD-10 codes used to identify hip fracture as an outcome in SAIL 68](#_Toc393804842)

[Table S16 List of Read codes used to identify acute cardiac events as an outcome in SAIL 69](#_Toc393804843)

[Table S17 List of ICD-10 codes used to identify acute cardiac events as an outcome in SAIL 71](#_Toc393804844)

[Table S18 List of Read codes used to identify DVT/PE as an outcome in SAIL 72](#_Toc393804845)

[Table S19 List of ICD-10 codes used to identify DVT/PE as an outcome in SAIL 73](#_Toc393804846)

[Table S20 List of Read codes used to identify antipsychotic prescriptions in SAIL 74](#_Toc393804847)

[Table S21 List of Read codes used to identify prescription of hypnotics in SAIL 85](#_Toc393804848)

[Table S22 List of Read codes used to identify prescription of anxiolytics in SAIL 88](#_Toc393804849)

[Table S23 List of Read codes used to identify prescription of benzodiazepines in SAIL 92](#_Toc393804850)

# Table S 1 List of Read codes used to identify psychosis as an exclusion in SAIL

| **Read Code** | **Text Description** |
| --- | --- |
| 1464 . | H/O: schizophrenia |
| E10.. | Schizophrenic disorders |
| E100. | Simple schizophrenia |
| E1000 | Unspecified schizophrenia |
| E1001 | Subchronic schizophrenia |
| E1002 | Chronic schizophrenic |
| E1003 | Acute exacerbation of subchronic schizophrenia |
| E1004 | Acute exacerbation of chronic schizophrenia |
| E1005 | Schizophrenia in remission |
| E100z | Simple schizophrenia NOS |
| E101. | Hebephrenic schizophrenia |
| E1010 | Unspecified hebephrenic schizophrenia |
| E1011 | Subchronic hebephrenic schizophrenia |
| E1012 | Chronic hebephrenic schizophrenia |
| E1013 | Acute exacerbation of subchronic hebephrenic schizophrenia |
| E1014 | Acute exacerbation of chronic hebephrenic schizophrenia |
| E1015 | Hebephrenic schizophrenia in remission |
| E101z | Hebephrenic schizophrenia NOS |
| E102. | Catatonic schizophrenia |
| E1020 | Unspecified catatonic schizophrenia |
| E1021 | Subchronic catatonic schizophrenia |
| E1022 | Chronic catatonic schizophrenia |
| E1023 | Acute exacerbation of subchronic catatonic schizophrenia |
| E1024 | Acute exacerbation of chronic catatonic schizophrenia |
| E1025 | Catatonic schizophrenia in remission |
| E102z | Catatonic schizophrenia NOS |
| E103. | Paranoid schizophrenia |
| E1030 | Unspecified paranoid schizophrenia |
| E1031 | Subchronic paranoid schizophrenia |
| E1032 | Chronic paranoid schizophrenia |
| E1033 | Acute exacerbation of subchronic paranoid schizophrenia |
| E1034 | Acute exacerbation of chronic paranoid schizophrenia |
| E1035 | Paranoid schizophrenia in remission |
| E103z | Paranoid schizophrenia NOS |
| E104. | Acute schizophrenic episode |
| E105. | Latent schizophrenia |
| E1050 | Unspecified latent schizophrenia |
| E1051 | Subchronic latent schizophrenia |
| E1052 | Chronic latent schizophrenia |
| E1053 | Acute exacerbation of subchronic latent schizophrenia |
| E1054 | Acute exacerbation of chronic latent schizophrenia |
| E1055 | Latent schizophrenia in remission |
| E105z | Latent schizophrenia NOS |
| E106. | Residual schizophrenia |
| E107. | Schizo-affective schizophrenia |
| E1070 | Unspecified schizo-affective schizophrenia |
| E1071 | Subchronic schizo-affective schizophrenia |
| E1072 | Chronic schizo-affective schizophrenia |
| E1073 | Acute exacerbation of subchronic schizo-affective schizophrenia |
| E1074 | Acute exacerbation of chronic schizo-affective schizophrenia |
| E1075 | Schizo-affective schizophrenia in remission |
| E107z | Schizo-affective schizophrenia NOS |
| E10y. | Other schizophrenia |
| E10y0 | Atypical schizophrenia |
| E10y1 | Coenesthopathic schizophrenia |
| E10yz | other schizophrenia |
| E10z. | Schizophrenia NOS |
| ZV110 | [V]Personal history of schizophrenia |
| Eu2.. | [X]Schizophrenia, schizotypal and delusional disorders |
| Eu20. | [X]Schizophrenia |
| Eu200 | [X]Paranoid schizophrenia |
| Eu201 | [X]Hebephrenic schizophrenia |
| Eu202 | [X]Catatonic schizophrenia |
| Eu203 | [X]Undifferentiated schizophrenia |
| Eu204 | [X]Post-schizophrenic depression |
| Eu205 | [X]Residual schizophrenia |
| Eu206 | [X]Simple schizophrenia |
| Eu20y | [X]Other schizophrenia |
| Eu20z | [X]Schizophrenia, unspecified |
| Eu22. | [X]Persistent delusional disorders |
| Eu220 | [X]Delusional disorder |
| Eu221 | [X]Delusional misidentification syndrome |
| Eu222 | [X]Cotard syndrome |
| Eu22y | [X]Other persistent delusional disorders |
| Eu22z | [X]Persistent delusional disorder, unspecified |
| Eu25. | [X]Schizoaffective disorders |
| Eu250 | [X]Schizoaffective disorder, manic type |
| Eu251 | [X]Schizoaffective disorder, depressive type |
| Eu252 | [X]Schizoaffective disorder, mixed type |
| Eu25y | [X]Other schizoaffective disorders |
| Eu25z | [X]Schizoaffective disorder, unspecified |
| E11.. | Bipolar psychoses |
| E114. | Bipolar affective disorder, currently manic |
| E1140 | Bipolar affective disorder, currently manic, unspecified |
| E1141 | Bipolar affective disorder, currently manic, mild |
| E1142 | Bipolar affective disorder, currently manic, moderate |
| E1143 | Bipolar affective disorder, currently manic, severe, without mention of psychosis |
| E1144 | Bipolar affective disorder, currently manic, severe, with psychosis |
| E1145 | Bipolar affective disorder, currently manic, in partial or unspecified remission |
| E1146 | Bipolar affective disorder, currently manic, in full remission |
| E114z | Bipolar affective disorder, currently manic, NOS |
| E115. | Bipolar affective disorder, currently depressed |
| E1150 | Bipolar affective disorder, currently depressed, unspecified |
| E1151 | Bipolar affective disorder, currently depressed, mild |
| E1152 | Bipolar affective disorder, currently depressed, moderate |
| E1153 | Bipolar affective disorder, currently depressed, severe, without mention of psychosis |
| E1154 | Bipolar affective disorder, currently depressed, severe, with psychosis |
| E1155 | Bipolar affective disorder, currently depressed, in partial or unspecified remission |
| E1156 | Bipolar affective disorder, currently depressed, in full remission |
| E115z | Bipolar affective disorder, currently depressed, NOS |
| E116. | Mixed bipolar affective disorder |
| E1160 | Mixed bipolar affective disorder, unspecified |
| E1161 | Mixed bipolar affective disorder, mild |
| E1162 | Mixed bipolar affective disorder, moderate |
| E1163 | Mixed bipolar affective disorder, severe, without mention of psychosis |
| E1164 | Mixed bipolar affective disorder, severe, with psychosis |
| E1165 | Mixed bipolar affective disorder, in partial or unspecified remission |
| E1166 | Mixed bipolar affective disorder, in full remission |
| E116z | Mixed bipolar affective disorder, NOS |
| E117. | Unspecified bipolar affective disorder |
| E1170 | Unspecified bipolar affective disorder, unspecified |
| E1171 | Unspecified bipolar affective disorder, mild |
| E1172 | Unspecified bipolar affective disorder, moderate |
| E1173 | Unspecified bipolar affective disorder, severe, without mention of psychosis |
| E1174 | Unspecified bipolar affective disorder, severe, with psychosis |
| E1175 | Unspecified bipolar affective disorder, in partial or unspecified remission |
| E1176 | Unspecified bipolar affective disorder, in full remission |
| E117z | Unspecified bipolar affective disorder, NOS |
| E11y. | Other and unspecified manic-depressive psychoses |
| E11y0 | Unspecified manic-depressive psychoses |
| E11y1 | Atypical manic disorder |
| E11y3 | Other mixed manic-depressive psychoses |
| E11yz | Other and unspecified manic-depressive psychoses NOS |
| Eu31. | [X]Bipolar affective disorder |
| Eu310 | [X]Bipolar affective disorder, current episode hypomanic |
| Eu311 | [X]Bipolar affective disorder, current episode manic without psychotic symptoms |
| Eu312 | [X]Bipolar affective disorder, current episode manic with psychotic symptoms |
| Eu313 | [X]Bipolar affective disorder, current episode mild or moderate depression |
| Eu314 | [X]Bipolar affective disorder, current episode severe depression without psychotic symptoms |
| Eu315 | [X]Bipolar affective disorder, current episode severe depression with psychotic symptoms |
| Eu316 | [X]Bipolar affective disorder, current episode mixed |
| Eu317 | [X]Bipolar affective disorder, currently in remission |
| Eu31y | [X]Other bipolar affective disorders |
| Eu31z | [X]Bipolar affective disorder, unspecified |
| 212V. | Bipolar affective disorder resolved |
| Eu30. | [X]Bipolar disorder, single manic episode |
| Eu300 | [X]Hypomania |
| Eu301 | [X]Mania without psychotic symptoms |
| Eu302 | [X]Mania with psychotic symptoms |
| Eu30y | [X]Other manic episodes |
| Eu30z | [X]Manic episode, unspecified |
| 212T. | Psychosis, schizophrenia and bipolar affective disorder resolved |

# Table S 2 List of Read codes used to identify and exclude cancer in SAIL

| **Read Code** | **Text description** |
| --- | --- |
| B072. | Malignant neoplasm of lateral wall of nasopharynx |
| 209 | MYELOFIBROSIS |
| 1538A | MALIGNANT NEOPLASM LARGE INTESTINE |
| 1619A | NEOPLASM MALIGNANT LARYNX |
| 1723C | CARCINOMA NOSE EXTERNAL |
| 1929M | MENINGIOMA BRAIN MALIGNANT |
| 2102M | TUMOUR MIXED PAROTID |
| 2104D | ODONTOMA |
| B.... | Cancers |
| B.... | Neoplasms |
| B0... | Carcinoma of lip, oral cavity and pharynx |
| B0... | Malignant neoplasm of lip, oral cavity and pharynx |
| B00.. | Malignant neoplasm of lip |
| B00.. | Carcinoma of lip |
| B000. | Malignant neoplasm of upper lip, vermilion border |
| B0000 | Malignant neoplasm of upper lip, external |
| B0001 | Malignant neoplasm of upper lip, lipstick area |
| B000z | Malignant neoplasm of upper lip, vermilion border NOS |
| B001. | Malignant neoplasm of lower lip, vermilion border |
| B0010 | Malignant neoplasm of lower lip, external |
| B0011 | Malignant neoplasm of lower lip, lipstick area |
| B001z | Malignant neoplasm of lower lip, vermilion border NOS |
| B002. | Malignant neoplasm of upper lip, inner aspect |
| B0020 | Malignant neoplasm of upper lip, buccal aspect |
| B0021 | Malignant neoplasm of upper lip, frenulum |
| B0022 | Malignant neoplasm of upper lip, mucosa |
| B0023 | Malignant neoplasm of upper lip, oral aspect |
| B002z | Malignant neoplasm of upper lip, inner aspect NOS |
| B003. | Malignant neoplasm of lower lip, inner aspect |
| B0030 | Malignant neoplasm of lower lip, buccal aspect |
| B0031 | Malignant neoplasm of lower lip, frenulum |
| B0032 | Malignant neoplasm of lower lip, mucosa |
| B0033 | Malignant neoplasm of lower lip, oral aspect |
| B003z | Malignant neoplasm of lower lip, inner aspect NOS |
| B004. | Malignant neoplasm of lip unspecified, inner aspect |
| B0040 | Malignant neoplasm of lip unspecified, buccal aspect |
| B0041 | Malignant neoplasm of lip unspecified, frenulum |
| B0042 | Malignant neoplasm of lip unspecified, mucosa |
| B0043 | Malignant neoplasm of lip, oral aspect |
| B004z | Malignant neoplasm of lip, inner aspect NOS |
| B005. | Malignant neoplasm of commissure of lip |
| B006. | Malignant neoplasm of overlapping lesion of lip |
| B007. | Malignant neoplasm of lip, unspecified |
| B00y. | Malignant neoplasm of other sites of lip |
| B00z. | Malignant neoplasm of vermilion border of lip unspecified |
| B00z0 | Malignant neoplasm of lip, unspecified, external |
| B00z1 | Malignant neoplasm of lip, unspecified, lipstick area |
| B00zz | Malignant neoplasm of lip, vermilion border NOS |
| B01.. | Malignant neoplasm of tongue |
| B010. | Malignant neoplasm of base of tongue |
| B010. | Malignant neoplasm of posterior third of tongue |
| B0100 | Malignant neoplasm of base of tongue dorsal surface |
| B010z | Malignant neoplasm of fixed part of tongue NOS |
| B011. | Malignant neoplasm of dorsal surface of tongue |
| B0110 | Malignant neoplasm of anterior 2/3 of tongue dorsal surface |
| B0111 | Malignant neoplasm of midline of tongue |
| B011z | Malignant neoplasm of dorsum of tongue NOS |
| B012. | Malignant neoplasm of tongue, tip and lateral border |
| B013. | Malignant neoplasm of ventral surface of tongue |
| B0130 | Malignant neoplasm of anterior 2/3 of tongue ventral surface |
| B0131 | Malignant neoplasm of frenulum linguae |
| B013z | Malignant neoplasm of ventral tongue surface NOS |
| B014. | Malignant neoplasm of anterior 2/3 of tongue unspecified |
| B015. | Malignant neoplasm of tongue, junctional zone |
| B016. | Malignant neoplasm of lingual tonsil |
| B017. | Malignant overlapping lesion of tongue |
| B01y. | Malignant neoplasm of other sites of tongue |
| B01z. | Malignant neoplasm of tongue NOS |
| B02.. | Malignant neoplasm of major salivary glands |
| B020. | Malignant neoplasm of parotid gland |
| B021. | Malignant neoplasm of submandibular gland |
| B022. | Malignant neoplasm of sublingual gland |
| B023. | Malignant neoplasm, overlapping lesion of major saliv gland |
| B02y. | Malignant neoplasm of other major salivary glands |
| B02z. | Malignant neoplasm of major salivary gland NOS |
| B03.. | Malignant neoplasm of gum |
| B030. | Malignant neoplasm of upper gum |
| B031. | Malignant neoplasm of lower gum |
| B03y. | Malignant neoplasm of other sites of gum |
| B03z. | Malignant neoplasm of gum NOS |
| B04.. | Malignant neoplasm of floor of mouth |
| B040. | Malignant neoplasm of anterior portion of floor of mouth |
| B041. | Malignant neoplasm of lateral portion of floor of mouth |
| B042. | Malignant neoplasm, overlapping lesion of floor of mouth |
| B04y. | Malignant neoplasm of other sites of floor of mouth |
| B04z. | Malignant neoplasm of floor of mouth NOS |
| B05.. | Malignant neoplasm of other and unspecified parts of mouth |
| B050. | Malignant neoplasm of buccal mucosa |
| B050. | Malignant neoplasm of cheek mucosa |
| B051. | Malignant neoplasm of vestibule of mouth |
| B0510 | Malignant neoplasm of upper buccal sulcus |
| B0511 | Malignant neoplasm of lower buccal sulcus |
| B0512 | Malignant neoplasm of upper labial sulcus |
| B0513 | Malignant neoplasm of lower labial sulcus |
| B051z | Malignant neoplasm of vestibule of mouth NOS |
| B052. | Malignant neoplasm of hard palate |
| B053. | Malignant neoplasm of soft palate |
| B054. | Malignant neoplasm of uvula |
| B055. | Malignant neoplasm of palate unspecified |
| B0550 | Malignant neoplasm of junction of hard and soft palate |
| B0551 | Malignant neoplasm of roof of mouth |
| B055z | Malignant neoplasm of palate NOS |
| B056. | Malignant neoplasm of retromolar area |
| B057. | Overlapping lesion of other and unspecified parts of mouth |
| B05y. | Malignant neoplasm of other specified mouth parts |
| B05z. | Malignant neoplasm of mouth NOS |
| B05z0 | Kaposi's sarcoma of palate |
| B06.. | Malignant neoplasm of oropharynx |
| B060. | Malignant neoplasm of tonsil |
| B0600 | Malignant neoplasm of faucial tonsil |
| B0601 | Malignant neoplasm of palatine tonsil |
| B0602 | Malignant neoplasm of overlapping lesion of tonsil |
| B060z | Malignant neoplasm tonsil NOS |
| B061. | Malignant neoplasm of tonsillar fossa |
| B062. | Malignant neoplasm of tonsillar pillar |
| B0620 | Malignant neoplasm of faucial pillar |
| B0621 | Malignant neoplasm of glossopalatine fold |
| B0622 | Malignant neoplasm of palatoglossal arch |
| B0623 | Malignant neoplasm of palatopharyngeal arch |
| B062z | Malignant neoplasm of tonsillar fossa NOS |
| B063. | Malignant neoplasm of vallecula |
| B064. | Malignant neoplasm of anterior epiglottis |
| B0640 | Malignant neoplasm of epiglottis, free border |
| B0641 | Malignant neoplasm of glossoepiglottic fold |
| B064z | Malignant neoplasm of anterior epiglottis NOS |
| B065. | Malignant neoplasm of junctional region of epiglottis |
| B066. | Malignant neoplasm of lateral wall of oropharynx |
| B067. | Malignant neoplasm of posterior wall of oropharynx |
| B06y. | Malignant neoplasm of oropharynx, other specified sites |
| B06y0 | Malignant neoplasm of branchial cleft |
| B06yz | Malignant neoplasm of other specified site of oropharynx NOS |
| B06z. | Malignant neoplasm of oropharynx NOS |
| B07.. | Malignant neoplasm of nasopharynx |
| B070. | Malignant neoplasm of roof of nasopharynx |
| B071. | Malignant neoplasm of posterior wall of nasopharynx |
| B0710 | Malignant neoplasm of adenoid |
| B0711 | Malignant neoplasm of pharyngeal tonsil |
| B071z | Malignant neoplasm of posterior wall of nasopharynx NOS |
| B0720 | Malignant neoplasm of pharyngeal recess |
| B0721 | Malignant neoplasm of opening of auditory tube |
| B072z | Malignant neoplasm of lateral wall of nasopharynx NOS |
| B073. | Malignant neoplasm of anterior wall of nasopharynx |
| B0730 | Malignant neoplasm of floor of nasopharynx |
| B0731 | Malignant neoplasm of nasopharyngeal soft palate surface |
| B0732 | Malignant neoplasm posterior margin nasal septum and choanae |
| B073z | Malignant neoplasm of anterior wall of nasopharynx NOS |
| B074. | Malignant neoplasm, overlapping lesion of nasopharynx |
| B07y. | Malignant neoplasm of other specified site of nasopharynx |
| B07z. | Malignant neoplasm of nasopharynx NOS |
| B08.. | Malignant neoplasm of hypopharynx |
| B080. | Malignant neoplasm of postcricoid region |
| B081. | Malignant neoplasm of pyriform sinus |
| B082. | Malignant neoplasm aryepiglottic fold, hypopharyngeal aspect |
| B083. | Malignant neoplasm of posterior pharynx |
| B084. | Malignant neoplasm, overlapping lesion of hypopharynx |
| B08y. | Malignant neoplasm of other specified hypopharyngeal site |
| B08z. | Malignant neoplasm of hypopharynx NOS |
| B0z.. | Malig neop other/ill-defined sites lip, oral cavity, pharynx |
| B0z0. | Malignant neoplasm of pharynx unspecified |
| B0z1. | Malignant neoplasm of Waldeyer's ring |
| B0z2. | Malignant neoplasm of laryngopharynx |
| B0zy. | Malignant neoplasm of other sites lip, oral cavity, pharynx |
| B0zz. | Malignant neoplasm of lip, oral cavity and pharynx NOS |
| B1... | Carcinoma of digestive organs and peritoneum |
| B1... | Malignant neoplasm of digestive organs and peritoneum |
| B10.. | Malignant neoplasm of oesophagus |
| B100. | Malignant neoplasm of cervical oesophagus |
| B101. | Malignant neoplasm of thoracic oesophagus |
| B102. | Malignant neoplasm of abdominal oesophagus |
| B103. | Malignant neoplasm of upper third of oesophagus |
| B104. | Malignant neoplasm of middle third of oesophagus |
| B105. | Malignant neoplasm of lower third of oesophagus |
| B106. | Malignant neoplasm, overlapping lesion of oesophagus |
| B10y. | Malignant neoplasm of other specified part of oesophagus |
| B10z. | Malignant neoplasm of oesophagus NOS |
| B10z. | Oesophageal cancer |
| B11.. | Malignant neoplasm of stomach |
| B11.. | Gastric neoplasm |
| B110. | Malignant neoplasm of cardia of stomach |
| B1100 | Malignant neoplasm of cardiac orifice of stomach |
| B1101 | Malignant neoplasm of cardio-oesophageal junction of stomach |
| B1101 | Malignant neoplasm of gastro-oesophageal junction |
| B110z | Malignant neoplasm of cardia of stomach NOS |
| B111. | Malignant neoplasm of pylorus of stomach |
| B1110 | Malignant neoplasm of prepylorus of stomach |
| B1111 | Malignant neoplasm of pyloric canal of stomach |
| B111z | Malignant neoplasm of pylorus of stomach NOS |
| B112. | Malignant neoplasm of pyloric antrum of stomach |
| B113. | Malignant neoplasm of fundus of stomach |
| B114. | Malignant neoplasm of body of stomach |
| B115. | Malignant neoplasm of lesser curve of stomach unspecified |
| B116. | Malignant neoplasm of greater curve of stomach unspecified |
| B117. | Malignant neoplasm, overlapping lesion of stomach |
| B11y. | Malignant neoplasm of other specified site of stomach |
| B11y0 | Malignant neoplasm of anterior wall of stomach NEC |
| B11y1 | Malignant neoplasm of posterior wall of stomach NEC |
| B11yz | Malignant neoplasm of other specified site of stomach NOS |
| B11z. | Malignant neoplasm of stomach NOS |
| B12.. | Malignant neoplasm of small intestine and duodenum |
| B120. | Malignant neoplasm of duodenum |
| B121. | Malignant neoplasm of jejunum |
| B122. | Malignant neoplasm of ileum |
| B123. | Malignant neoplasm of Meckel's diverticulum |
| B124. | Malignant neoplasm, overlapping lesion of small intestine |
| B12y. | Malignant neoplasm of other specified site small intestine |
| B12z. | Malignant neoplasm of small intestine NOS |
| B13.. | Malignant neoplasm of colon |
| B130. | Malignant neoplasm of hepatic flexure of colon |
| B131. | Malignant neoplasm of transverse colon |
| B132. | Malignant neoplasm of descending colon |
| B133. | Malignant neoplasm of sigmoid colon |
| B134. | Carcinoma of caecum |
| B134. | Malignant neoplasm of caecum |
| B135. | Malignant neoplasm of appendix |
| B136. | Malignant neoplasm of ascending colon |
| B137. | Malignant neoplasm of splenic flexure of colon |
| B138. | Malignant neoplasm, overlapping lesion of colon |
| B13y. | Malignant neoplasm of other specified sites of colon |
| B13z. | Malignant neoplasm of colon NOS |
| B13z. | Colonic cancer |
| B14.. | Malignant neoplasm of rectum, rectosigmoid junction and anus |
| B140. | Malignant neoplasm of rectosigmoid junction |
| B141. | Rectal carcinoma |
| B141. | Malignant neoplasm of rectum |
| B141. | Carcinoma of rectum |
| B142. | Anal carcinoma |
| B142. | Malignant neoplasm of anal canal |
| B1420 | Malignant neoplasm of cloacogenic zone |
| B143. | Malignant neoplasm of anus unspecified |
| B14y. | Malig neop other site rectum, rectosigmoid junction and anus |
| B14z. | Malignant neoplasm rectum,rectosigmoid junction and anus NOS |
| B15.. | Malignant neoplasm of liver and intrahepatic bile ducts |
| B150. | Primary malignant neoplasm of liver |
| B1500 | Primary carcinoma of liver |
| B1501 | Hepatoblastoma of liver |
| B1502 | Primary angiosarcoma of liver |
| B1503 | Hepatocellular carcinoma |
| B150z | Primary malignant neoplasm of liver NOS |
| B151. | Malignant neoplasm of intrahepatic bile ducts |
| B1510 | Malignant neoplasm of interlobular bile ducts |
| B1511 | Malignant neoplasm of interlobular biliary canals |
| B1512 | Malignant neoplasm of intrahepatic biliary passages |
| B1513 | Malignant neoplasm of intrahepatic canaliculi |
| B1514 | Malignant neoplasm of intrahepatic gall duct |
| B151z | Malignant neoplasm of intrahepatic bile ducts NOS |
| B152. | Malignant neoplasm of liver unspecified |
| B15z. | Malignant neoplasm of liver and intrahepatic bile ducts NOS |
| B16.. | Malignant neoplasm gallbladder and extrahepatic bile ducts |
| B160. | Carcinoma gallbladder |
| B160. | Malignant neoplasm of gallbladder |
| B161. | Malignant neoplasm of extrahepatic bile ducts |
| B1610 | Malignant neoplasm of cystic duct |
| B1611 | Malignant neoplasm of hepatic duct |
| B1612 | Malignant neoplasm of common bile duct |
| B1612 | Carcinoma common bile duct |
| B1613 | Malignant neoplasm of sphincter of Oddi |
| B161z | Malignant neoplasm of extrahepatic bile ducts NOS |
| B162. | Malignant neoplasm of ampulla of Vater |
| B163. | Malignant neoplasm, overlapping lesion of biliary tract |
| B16y. | Malignant neoplasm other gallbladder/extrahepatic bile duct |
| B16z. | Malignant neoplasm gallbladder/extrahepatic bile ducts NOS |
| B17.. | Malignant neoplasm of pancreas |
| B170. | Malignant neoplasm of head of pancreas |
| B171. | Malignant neoplasm of body of pancreas |
| B172. | Malignant neoplasm of tail of pancreas |
| B173. | Malignant neoplasm of pancreatic duct |
| B174. | Malignant neoplasm of Islets of Langerhans |
| B175. | Malignant neoplasm, overlapping lesion of pancreas |
| B17y. | Malignant neoplasm of other specified sites of pancreas |
| B17y0 | Malignant neoplasm of ectopic pancreatic tissue |
| B17yz | Malignant neoplasm of specified site of pancreas NOS |
| B17z. | Malignant neoplasm of pancreas NOS |
| B18.. | Malignant neoplasm of retroperitoneum and peritoneum |
| B180. | Malignant neoplasm of retroperitoneum |
| B1800 | Malignant neoplasm of periadrenal tissue |
| B1801 | Malignant neoplasm of perinephric tissue |
| B1802 | Malignant neoplasm of retrocaecal tissue |
| B180z | Malignant neoplasm of retroperitoneum NOS |
| B181. | Mesothelioma of peritoneum |
| B182. | Overlapping malign lesion of retroperitoneum and peritoneum |
| B18y. | Malignant neoplasm of specified parts of peritoneum |
| B18y0 | Malignant neoplasm of mesocolon |
| B18y1 | Malignant neoplasm of mesocaecum |
| B18y2 | Malignant neoplasm of mesorectum |
| B18y3 | Malignant neoplasm of omentum |
| B18y4 | Malignant neoplasm of parietal peritoneum |
| B18y5 | Malignant neoplasm of pelvic peritoneum |
| B18y6 | Malignant neoplasm of the pouch of Douglas |
| B18y7 | Malignant neoplasm of mesentery |
| B18yz | Malignant neoplasm of specified parts of peritoneum NOS |
| B18z. | Malignant neoplasm of retroperitoneum and peritoneum NOS |
| B1z.. | Malig neop oth/ill-defined sites digestive tract/peritoneum |
| B1z0. | Malignant neoplasm of intestinal tract, part unspecified |
| B1z0. | Cancer of bowel |
| B1z1. | Malignant neoplasm of spleen NEC |
| B1z10 | Angiosarcoma of spleen |
| B1z11 | Fibrosarcoma of spleen |
| B1z1z | Malignant neoplasm of spleen NOS |
| B1z2. | Malignant neoplasm, overlapping lesion of digestive system |
| B1zy. | Malignant neoplasm other spec digestive tract and peritoneum |
| B1zz. | Malignant neoplasm of digestive tract and peritoneum NOS |
| B2... | Carcinoma of respiratory tract and intrathoracic organs |
| B2... | Malig neop of respiratory tract and intrathoracic organs |
| B20.. | Malig neop nasal cavities, middle ear and accessory sinuses |
| B200. | Malignant neoplasm of nasal cavities |
| B2000 | Malignant neoplasm of cartilage of nose |
| B2001 | Malignant neoplasm of nasal conchae |
| B2002 | Malignant neoplasm of septum of nose |
| B2003 | Malignant neoplasm of vestibule of nose |
| B200z | Malignant neoplasm of nasal cavities NOS |
| B201. | Malig neop auditory tube, middle ear and mastoid air cells |
| B2010 | Malignant neoplasm of auditory (Eustachian) tube |
| B2011 | Malignant neoplasm of tympanic cavity |
| B2012 | Malignant neoplasm of tympanic antrum |
| B2013 | Malignant neoplasm of mastoid air cells |
| B201z | Malig neop auditory tube, middle ear, mastoid air cells NOS |
| B202. | Malignant neoplasm of maxillary sinus |
| B203. | Malignant neoplasm of ethmoid sinus |
| B204. | Malignant neoplasm of frontal sinus |
| B205. | Malignant neoplasm of sphenoidal sinus |
| B206. | Malignant neoplasm, overlapping lesion of accessory sinuses |
| B20y. | Malig neop other site nasal cavity, middle ear and sinuses |
| B20z. | Malignant neoplasm of accessory sinus NOS |
| B21.. | Malignant neoplasm of larynx |
| B210. | Malignant neoplasm of glottis |
| B211. | Malignant neoplasm of supraglottis |
| B212. | Malignant neoplasm of subglottis |
| B213. | Malignant neoplasm of laryngeal cartilage |
| B2130 | Malignant neoplasm of arytenoid cartilage |
| B2131 | Malignant neoplasm of cricoid cartilage |
| B2132 | Malignant neoplasm of cuneiform cartilage |
| B2133 | Malignant neoplasm of thyroid cartilage |
| B213z | Malignant neoplasm of laryngeal cartilage NOS |
| B214. | Malignant neoplasm, overlapping lesion of larynx |
| B215. | Malignant neoplasm of epiglottis NOS |
| B21y. | Malignant neoplasm of larynx, other specified site |
| B21z. | Malignant neoplasm of larynx NOS |
| B22.. | Malignant neoplasm of trachea, bronchus and lung |
| B220. | Malignant neoplasm of trachea |
| B2200 | Malignant neoplasm of cartilage of trachea |
| B2201 | Malignant neoplasm of mucosa of trachea |
| B220z | Malignant neoplasm of trachea NOS |
| B221. | Malignant neoplasm of main bronchus |
| B2210 | Malignant neoplasm of carina of bronchus |
| B2211 | Malignant neoplasm of hilus of lung |
| B221z | Malignant neoplasm of main bronchus NOS |
| B222. | Malignant neoplasm of upper lobe, bronchus or lung |
| B222. | Pancoast's syndrome |
| B2220 | Malignant neoplasm of upper lobe bronchus |
| B2221 | Malignant neoplasm of upper lobe of lung |
| B222z | Malignant neoplasm of upper lobe, bronchus or lung NOS |
| B223. | Malignant neoplasm of middle lobe, bronchus or lung |
| B2230 | Malignant neoplasm of middle lobe bronchus |
| B2231 | Malignant neoplasm of middle lobe of lung |
| B223z | Malignant neoplasm of middle lobe, bronchus or lung NOS |
| B224. | Malignant neoplasm of lower lobe, bronchus or lung |
| B2240 | Malignant neoplasm of lower lobe bronchus |
| B2241 | Malignant neoplasm of lower lobe of lung |
| B224z | Malignant neoplasm of lower lobe, bronchus or lung NOS |
| B225. | Malignant neoplasm of overlapping lesion of bronchus & lung |
| B226. | Mesothelioma |
| B22y. | Malignant neoplasm of other sites of bronchus or lung |
| B22z. | Lung cancer |
| B22z. | Malignant neoplasm of bronchus or lung NOS |
| B23.. | Malignant neoplasm of pleura |
| B230. | Malignant neoplasm of parietal pleura |
| B231. | Malignant neoplasm of visceral pleura |
| B232. | Mesothelioma of pleura |
| B23y. | Malignant neoplasm of other specified pleura |
| B23z. | Malignant neoplasm of pleura NOS |
| B24.. | Malignant neoplasm of thymus, heart and mediastinum |
| B240. | Malignant neoplasm of thymus |
| B241. | Malignant neoplasm of heart |
| B2410 | Malignant neoplasm of endocardium |
| B2411 | Malignant neoplasm of epicardium |
| B2412 | Malignant neoplasm of myocardium |
| B2413 | Malignant neoplasm of pericardium |
| B2414 | Mesothelioma of pericardium |
| B241z | Malignant neoplasm of heart NOS |
| B242. | Malignant neoplasm of anterior mediastinum |
| B243. | Malignant neoplasm of posterior mediastinum |
| B24X. | Malignant neoplasm of mediastinum, part unspecified |
| B24y. | Malig neop of other site of heart, thymus and mediastinum |
| B24z. | Malignant neoplasm of heart, thymus and mediastinum NOS |
| B25.. | Malig neo, overlapping lesion of heart, mediastinum & pleura |
| B26.. | Malignant neoplasm, overlap lesion of resp & intrathor orgs |
| B2z.. | Malig neop other/ill-defined sites resp/intrathoracic organs |
| B2z0. | Malig neop of upper respiratory tract, part unspecified |
| B2zy. | Malignant neoplasm of other site of respiratory tract |
| B2zz. | Malignant neoplasm of respiratory tract NOS |
| B3... | Carcinoma of bone, connective tissue, skin and breast |
| B3... | Malig neop of bone, connective tissue, skin and breast |
| B3... | Sarcoma of bone and connective tissue |
| B30.. | Chondroma |
| B30.. | Osteoma |
| B30.. | Malignant neoplasm of bone and articular cartilage |
| B300. | Malignant neoplasm of bones of skull and face |
| B3000 | Malignant neoplasm of ethmoid bone |
| B3001 | Malignant neoplasm of frontal bone |
| B3002 | Malignant neoplasm of malar bone |
| B3003 | Malignant neoplasm of nasal bone |
| B3004 | Malignant neoplasm of occipital bone |
| B3005 | Malignant neoplasm of orbital bone |
| B3006 | Malignant neoplasm of parietal bone |
| B3007 | Malignant neoplasm of sphenoid bone |
| B3008 | Malignant neoplasm of temporal bone |
| B3009 | Malignant neoplasm of zygomatic bone |
| B300A | Malignant neoplasm of maxilla |
| B300C | Malignant neoplasm of vomer |
| B300z | Malignant neoplasm of bones of skull and face NOS |
| B301. | Malignant neoplasm of mandible |
| B302. | Malignant neoplasm of vertebral column |
| B3020 | Malignant neoplasm of cervical vertebra |
| B3021 | Malignant neoplasm of thoracic vertebra |
| B3022 | Malignant neoplasm of lumbar vertebra |
| B302z | Malignant neoplasm of vertebral column NOS |
| B303. | Malignant neoplasm of ribs, sternum and clavicle |
| B3030 | Malignant neoplasm of rib |
| B3031 | Malignant neoplasm of sternum |
| B3032 | Malignant neoplasm of clavicle |
| B3033 | Malignant neoplasm of costal cartilage |
| B3034 | Malignant neoplasm of costo-vertebral joint |
| B3035 | Malignant neoplasm of xiphoid process |
| B303z | Malignant neoplasm of rib, sternum and clavicle NOS |
| B304. | Malignant neoplasm of scapula and long bones of upper arm |
| B3040 | Malignant neoplasm of scapula |
| B3041 | Malignant neoplasm of acromion |
| B3042 | Malignant neoplasm of humerus |
| B3043 | Malignant neoplasm of radius |
| B3044 | Malignant neoplasm of ulna |
| B304z | Malig neop of scapula and long bones of upper arm NOS |
| B305. | Malignant neoplasm of carpal bones |
| B305. | Malignant neoplasm of metacarpal bones |
| B305. | Malignant neoplasm of hand bones |
| B3050 | Malignant neoplasm of carpal bone - scaphoid |
| B3051 | Malignant neoplasm of carpal bone - lunate |
| B3052 | Malignant neoplasm of carpal bone - triquetrum |
| B3053 | Malignant neoplasm of carpal bone - pisiform |
| B3054 | Malignant neoplasm of carpal bone - trapezium |
| B3055 | Malignant neoplasm of carpal bone - trapezoid |
| B3056 | Malignant neoplasm of carpal bone - capitate |
| B3057 | Malignant neoplasm of carpal bone - hamate |
| B3058 | Malignant neoplasm of first metacarpal bone |
| B3059 | Malignant neoplasm of second metacarpal bone |
| B305A | Malignant neoplasm of third metacarpal bone |
| B305C | Malignant neoplasm of fifth metacarpal bone |
| B305D | Malignant neoplasm of phalanges of hand |
| B305z | Malignant neoplasm of hand bones NOS |
| B306. | Malignant neoplasm of pelvic bones, sacrum and coccyx |
| B3060 | Malignant neoplasm of ilium |
| B3061 | Malignant neoplasm of ischium |
| B3062 | Malignant neoplasm of pubis |
| B3063 | Malignant neoplasm of sacral vertebra |
| B3064 | Malignant neoplasm of coccygeal vertebra |
| B3065 | Malignant sacral teratoma |
| B306z | Malignant neoplasm of pelvis, sacrum or coccyx NOS |
| B307. | Malignant neoplasm of long bones of leg |
| B3070 | Malignant neoplasm of femur |
| B3071 | Malignant neoplasm of fibula |
| B3072 | Malignant neoplasm of tibia |
| B307z | Malignant neoplasm of long bones of leg NOS |
| B308. | Malignant neoplasm of short bones of leg |
| B308. | Malignant neoplasm of metatarsal bones of foot |
| B3080 | Malignant neoplasm of patella |
| B3081 | Malignant neoplasm of talus |
| B3082 | Malignant neoplasm of calcaneum |
| B3083 | Malignant neoplasm of medial cuneiform |
| B3084 | Malignant neoplasm of intermediate cuneiform |
| B3085 | Malignant neoplasm of lateral cuneiform |
| B3086 | Malignant neoplasm of cuboid |
| B3087 | Malignant neoplasm of navicular |
| B3088 | Malignant neoplasm of first metatarsal bone |
| B3089 | Malignant neoplasm of second metatarsal bone |
| B308A | Malignant neoplasm of third metatarsal bone |
| B308C | Malignant neoplasm of fifth metatarsal bone |
| B308D | Malignant neoplasm of phalanges of foot |
| B308z | Malignant neoplasm of short bones of leg NOS |
| B309. | Malignant neoplasm, overlap les bone and artic cart of limbs |
| B30W. | Malignant neoplasm/overlap lesion/bone??? cartilage |
| B30X. | Malignant neoplasm/bones??? cartilage/limb,unspfd |
| B30z. | Malignant neoplasm of bone and articular cartilage NOS |
| B30z0 | Osteosarcoma |
| B31.. | Malignant neoplasm of connective and other soft tissue |
| B310. | Malig neop of connective and soft tissue head, face and neck |
| B3100 | Malignant neoplasm of soft tissue of head |
| B3101 | Malignant neoplasm of soft tissue of face |
| B3102 | Malignant neoplasm of soft tissue of neck |
| B3103 | Malignant neoplasm of cartilage of ear |
| B3104 | Malignant neoplasm of tarsus of eyelid |
| B3105 | Malignant neoplasm soft tissues of cervical spine |
| B310z | Malig neop connective and soft tissue head, face, neck NOS |
| B311. | Malig neop connective and soft tissue upper limb/shoulder |
| B3110 | Malignant neoplasm of connective and soft tissue of shoulder |
| B3111 | Malignant neoplasm of connective and soft tissue, upper arm |
| B3112 | Malignant neoplasm of connective and soft tissue of fore-arm |
| B3113 | Malignant neoplasm of connective and soft tissue of hand |
| B3114 | Malignant neoplasm of connective and soft tissue of finger |
| B3115 | Malignant neoplasm of connective and soft tissue of thumb |
| B311z | Malig neop connective soft tissue upper limb/shoulder NOS |
| B312. | Malig neop of connective and soft tissue of hip and leg |
| B3120 | Malignant neoplasm of connective and soft tissue of hip |
| B3121 | Malig neop of connective and soft tissue thigh and upper leg |
| B3122 | Malig neop connective and soft tissue of popliteal space |
| B3123 | Malig neop of connective and soft tissue of lower leg |
| B3124 | Malignant neoplasm of connective and soft tissue of foot |
| B3125 | Malignant neoplasm of connective and soft tissue of toe |
| B3126 | Malig neop of connective and soft tissue of great toe |
| B312z | Malig neop connective and soft tissue hip and leg NOS |
| B313. | Malignant neoplasm of connective and soft tissue of thorax |
| B3130 | Malignant neoplasm of connective and soft tissue of axilla |
| B3131 | Malignant neoplasm of diaphragm |
| B3132 | Malignant neoplasm of great vessels |
| B3133 | Malig neoplasm of connective and soft tissues of thor spine |
| B313z | Malig neop of connective and soft tissue of thorax NOS |
| B314. | Malignant neoplasm of connective and soft tissue of abdomen |
| B3140 | Malig neop of connective and soft tissue of abdominal wall |
| B3141 | Malig neoplasm of connective and soft tissues of lumb spine |
| B314z | Malig neop of connective and soft tissue of abdomen NOS |
| B315. | Malignant neoplasm of connective and soft tissue of pelvis |
| B3150 | Malignant neoplasm of connective and soft tissue of buttock |
| B3151 | Malig neop of connective and soft tissue of inguinal region |
| B3152 | Malignant neoplasm of connective and soft tissue of perineum |
| B3153 | Malig neopl of connective and soft tissue - sacrum or coccyx |
| B315z | Malig neop of connective and soft tissue of pelvis NOS |
| B316. | Malig neop of connective and soft tissue trunk unspecified |
| B317. | Malignant neoplasm, overlap lesion connective & soft tissue |
| B31y. | Malig neop connective and soft tissue other specified site |
| B31z. | Malignant neoplasm of connective and soft tissue, site NOS |
| B31z0 | Kaposi's sarcoma of soft tissue |
| B32.. | Malignant melanoma of skin |
| B320. | Malignant melanoma of lip |
| B321. | Malignant melanoma of eyelid including canthus |
| B322. | Malignant melanoma of ear and external auricular canal |
| B3220 | Malignant melanoma of auricle (ear) |
| B3221 | Malignant melanoma of external auditory meatus |
| B322z | Malignant melanoma of ear and external auricular canal NOS |
| B323. | Malignant melanoma of other and unspecified parts of face |
| B3230 | Malignant melanoma of external surface of cheek |
| B3231 | Malignant melanoma of chin |
| B3232 | Malignant melanoma of eyebrow |
| B3233 | Malignant melanoma of forehead |
| B3234 | Malignant melanoma of external surface of nose |
| B3235 | Malignant melanoma of temple |
| B323z | Malignant melanoma of face NOS |
| B324. | Malignant melanoma of scalp and neck |
| B3240 | Malignant melanoma of scalp |
| B3241 | Malignant melanoma of neck |
| B324z | Malignant melanoma of scalp and neck NOS |
| B325. | Malignant melanoma of trunk (excluding scrotum) |
| B3250 | Malignant melanoma of axilla |
| B3251 | Malignant melanoma of breast |
| B3252 | Malignant melanoma of buttock |
| B3253 | Malignant melanoma of groin |
| B3254 | Malignant melanoma of perianal skin |
| B3255 | Malignant melanoma of perineum |
| B3256 | Malignant melanoma of umbilicus |
| B3257 | Malignant melanoma of back |
| B3258 | Malignant melanoma of chest wall |
| B325z | Malignant melanoma of trunk, excluding scrotum, NOS |
| B326. | Malignant melanoma of upper limb and shoulder |
| B3260 | Malignant melanoma of shoulder |
| B3261 | Malignant melanoma of upper arm |
| B3262 | Malignant melanoma of fore-arm |
| B3263 | Malignant melanoma of hand |
| B3264 | Malignant melanoma of finger |
| B3265 | Malignant melanoma of thumb |
| B326z | Malignant melanoma of upper limb or shoulder NOS |
| B327. | Malignant melanoma of lower limb and hip |
| B3270 | Malignant melanoma of hip |
| B3271 | Malignant melanoma of thigh |
| B3272 | Malignant melanoma of knee |
| B3273 | Malignant melanoma of popliteal fossa area |
| B3274 | Malignant melanoma of lower leg |
| B3275 | Malignant melanoma of ankle |
| B3276 | Malignant melanoma of heel |
| B3277 | Malignant melanoma of foot |
| B3278 | Malignant melanoma of toe |
| B3279 | Malignant melanoma of great toe |
| B327z | Malignant melanoma of lower limb or hip NOS |
| B32y. | Malignant melanoma of other specified skin site |
| B32y0 | Overlapping malignant melanoma of skin |
| B32z. | Malignant melanoma of skin NOS |
| B33.. | Epithelioma |
| B33.. | Malignant neoplasm of sweat gland |
| B33.. | Malignant neoplasm of sebaceous gland |
| B33.. | Other malignant neoplasm of skin |
| B330. | Malignant neoplasm of skin of lip |
| B331. | Malignant neoplasm of eyelid including canthus |
| B3310 | Malignant neoplasm of canthus |
| B3311 | Malignant neoplasm of upper eyelid |
| B3312 | Malignant neoplasm of lower eyelid |
| B332. | Malignant neoplasm skin of ear and external auricular canal |
| B3320 | Malignant neoplasm of skin of auricle (ear) |
| B3321 | Malignant neoplasm of skin of external auditory meatus |
| B3322 | Malignant neoplasm of pinna NEC |
| B332z | Malig neop skin of ear and external auricular canal NOS |
| B333. | Malignant neoplasm skin of other and unspecified parts face |
| B3330 | Malignant neoplasm of skin of cheek, external |
| B3331 | Malignant neoplasm of skin of chin |
| B3332 | Malignant neoplasm of skin of eyebrow |
| B3333 | Malignant neoplasm of skin of forehead |
| B3334 | Malignant neoplasm of skin of nose (external) |
| B3335 | Malignant neoplasm of skin of temple |
| B333z | Malignant neoplasm skin other and unspec part of face NOS |
| B334. | Malignant neoplasm of scalp and skin of neck |
| B3340 | Malignant neoplasm of scalp |
| B3341 | Malignant neoplasm of skin of neck |
| B334z | Malignant neoplasm of scalp or skin of neck NOS |
| B335. | Malignant neoplasm of skin of trunk, excluding scrotum |
| B3350 | Malignant neoplasm of skin of axillary fold |
| B3351 | Malignant neoplasm of skin of chest, excluding breast |
| B3352 | Malignant neoplasm of skin of breast |
| B3353 | Malignant neoplasm of skin of abdominal wall |
| B3354 | Malignant neoplasm of skin of umbilicus |
| B3355 | Malignant neoplasm of skin of groin |
| B3356 | Malignant neoplasm of skin of perineum |
| B3357 | Malignant neoplasm of skin of back |
| B3358 | Malignant neoplasm of skin of buttock |
| B3359 | Malignant neoplasm of perianal skin |
| B335A | Malignant neoplasm of skin of scapular region |
| B335z | Malignant neoplasm of skin of trunk, excluding scrotum, NOS |
| B336. | Malignant neoplasm of skin of upper limb and shoulder |
| B3360 | Malignant neoplasm of skin of shoulder |
| B3361 | Malignant neoplasm of skin of upper arm |
| B3362 | Malignant neoplasm of skin of fore-arm |
| B3363 | Malignant neoplasm of skin of hand |
| B3364 | Malignant neoplasm of skin of finger |
| B3365 | Malignant neoplasm of skin of thumb |
| B336z | Malignant neoplasm of skin of upper limb or shoulder NOS |
| B337. | Malignant neoplasm of skin of lower limb and hip |
| B3370 | Malignant neoplasm of skin of hip |
| B3371 | Malignant neoplasm of skin of thigh |
| B3372 | Malignant neoplasm of skin of knee |
| B3373 | Malignant neoplasm of skin of popliteal fossa area |
| B3374 | Malignant neoplasm of skin of lower leg |
| B3375 | Malignant neoplasm of skin of ankle |
| B3376 | Malignant neoplasm of skin of heel |
| B3377 | Malignant neoplasm of skin of foot |
| B3378 | Malignant neoplasm of skin of toe |
| B3379 | Malignant neoplasm of skin of great toe |
| B337z | Malignant neoplasm of skin of lower limb or hip NOS |
| B339. | Dermatofibrosarcoma protuberans |
| B33X. | Malignant neoplasm overlapping lesion of skin |
| B33y. | Malignant neoplasm of other specified skin sites |
| B33z. | Malignant neoplasm of skin NOS |
| B33z0 | Kaposi's sarcoma of skin |
| B34.. | Malignant neoplasm of female breast |
| B34.. | Ca female breast |
| B340. | Malignant neoplasm of nipple and areola of female breast |
| B3400 | Malignant neoplasm of nipple of female breast |
| B3401 | Malignant neoplasm of areola of female breast |
| B340z | Malignant neoplasm of nipple or areola of female breast NOS |
| B341. | Malignant neoplasm of central part of female breast |
| B342. | Malignant neoplasm of upper-inner quadrant of female breast |
| B343. | Malignant neoplasm of lower-inner quadrant of female breast |
| B344. | Malignant neoplasm of upper-outer quadrant of female breast |
| B345. | Malignant neoplasm of lower-outer quadrant of female breast |
| B346. | Malignant neoplasm of axillary tail of female breast |
| B347. | Malignant neoplasm, overlapping lesion of breast |
| B34y. | Malignant neoplasm of other site of female breast |
| B34y0 | Malignant neoplasm of ectopic site of female breast |
| B34yz | Malignant neoplasm of other site of female breast NOS |
| B34z. | Malignant neoplasm of female breast NOS |
| B35.. | Malignant neoplasm of male breast |
| B350. | Malignant neoplasm of nipple and areola of male breast |
| B3500 | Malignant neoplasm of nipple of male breast |
| B3501 | Malignant neoplasm of areola of male breast |
| B350z | Malignant neoplasm of nipple or areola of male breast NOS |
| B35z. | Malignant neoplasm of other site of male breast |
| B35z0 | Malignant neoplasm of ectopic site of male breast |
| B35zz | Malignant neoplasm of male breast NOS |
| B3y.. | Malig neop of bone, connective tissue, skin and breast OS |
| B3z.. | Malig neop of bone, connective tissue, skin and breast NOS |
| B4... | Malignant neoplasm of genitourinary organ |
| B4... | Carcinoma of genitourinary organ |
| B40.. | Malignant neoplasm of uterus, part unspecified |
| B41.. | Malignant neoplasm of cervix uteri |
| B41.. | Cervical carcinoma (uterus) |
| B410. | Malignant neoplasm of endocervix |
| B4100 | Malignant neoplasm of endocervical canal |
| B4101 | Malignant neoplasm of endocervical gland |
| B410z | Malignant neoplasm of endocervix NOS |
| B411. | Malignant neoplasm of exocervix |
| B412. | Malignant neoplasm, overlapping lesion of cervix uteri |
| B41y. | Malignant neoplasm of other site of cervix |
| B41y0 | Malignant neoplasm of cervical stump |
| B41y1 | Malignant neoplasm of squamocolumnar junction of cervix |
| B41yz | Malignant neoplasm of other site of cervix NOS |
| B41z. | Malignant neoplasm of cervix uteri NOS |
| B42.. | Malignant neoplasm of placenta |
| B420. | Choriocarcinoma |
| B43.. | Malignant neoplasm of body of uterus |
| B430. | Malignant neoplasm of corpus uteri, excluding isthmus |
| B4300 | Malignant neoplasm of cornu of corpus uteri |
| B4301 | Malignant neoplasm of fundus of corpus uteri |
| B4302 | Malignant neoplasm of endometrium |
| B4302 | Malignant neoplasm of endometrium of corpus uteri |
| B4303 | Malignant neoplasm of myometrium of corpus uteri |
| B430z | Malignant neoplasm of corpus uteri NOS |
| B431. | Malignant neoplasm of isthmus of uterine body |
| B4310 | Malignant neoplasm of lower uterine segment |
| B431z | Malignant neoplasm of isthmus of uterine body NOS |
| B432. | Malignant neoplasm of overlapping lesion of corpus uteri |
| B43y. | Malignant neoplasm of other site of uterine body |
| B43z. | Malignant neoplasm of body of uterus NOS |
| B44.. | Malignant neoplasm of ovary and other uterine adnexa |
| B440. | Malignant neoplasm of ovary |
| B440. | Cancer of ovary |
| B441. | Malignant neoplasm of fallopian tube |
| B442. | Malignant neoplasm of broad ligament |
| B443. | Malignant neoplasm of parametrium |
| B444. | Malignant neoplasm of round ligament |
| B44y. | Malignant neoplasm of other site of uterine adnexa |
| B44z. | Malignant neoplasm of uterine adnexa NOS |
| B45.. | Malig neop of other and unspecified female genital organs |
| B450. | Malignant neoplasm of vagina |
| B4500 | Malignant neoplasm of Gartner's duct |
| B4501 | Malignant neoplasm of vaginal vault |
| B450z | Malignant neoplasm of vagina NOS |
| B451. | Malignant neoplasm of labia majora |
| B4510 | Malignant neoplasm of greater vestibular (Bartholin's) gland |
| B451z | Malignant neoplasm of labia majora NOS |
| B452. | Malignant neoplasm of labia minora |
| B453. | Malignant neoplasm of clitoris |
| B454. | Malignant neoplasm of vulva unspecified |
| B454. | Primary vulval cancer |
| B45X. | Malignant neoplasm/overlapping lesion/feml genital organs |
| B45y. | Malignant neoplasm of other specified female genital organ |
| B45y0 | Malignant neoplasm of overlapping lesion of vulva |
| B45z. | Malignant neoplasm of female genital organ NOS |
| B46.. | Malignant neoplasm of prostate |
| B47.. | Malignant neoplasm of testis |
| B470. | Malignant neoplasm of undescended testis |
| B4700 | Malignant neoplasm of ectopic testis |
| B4701 | Malignant neoplasm of retained testis |
| B4702 | Seminoma of undescended testis |
| B4703 | Teratoma of undescended testis |
| B470z | Malignant neoplasm of undescended testis NOS |
| B471. | Malignant neoplasm of descended testis |
| B4710 | Seminoma of descended testis |
| B4711 | Teratoma of descended testis |
| B47z. | Malignant neoplasm of testis NOS |
| B47z. | Teratoma of testis |
| B47z. | Seminoma of testis |
| B48.. | Malignant neoplasm of penis and other male genital organs |
| B480. | Malignant neoplasm of prepuce (foreskin) |
| B481. | Malignant neoplasm of glans penis |
| B482. | Malignant neoplasm of body of penis |
| B483. | Malignant neoplasm of penis, part unspecified |
| B484. | Malignant neoplasm of epididymis |
| B485. | Malignant neoplasm of spermatic cord |
| B486. | Malignant neoplasm of scrotum |
| B487. | Malignant neoplasm, overlapping lesion of penis |
| B48y. | Malignant neoplasm of other male genital organ |
| B48y0 | Malignant neoplasm of seminal vesicle |
| B48y1 | Malignant neoplasm of tunica vaginalis |
| B48y2 | Malignant neoplasm, overlapping lesion male genital orgs |
| B48yz | Malignant neoplasm of other male genital organ NOS |
| B48z. | Malignant neoplasm of penis and other male genital organ NOS |
| B49.. | Malignant neoplasm of urinary bladder |
| B490. | Malignant neoplasm of trigone of urinary bladder |
| B491. | Malignant neoplasm of dome of urinary bladder |
| B492. | Malignant neoplasm of lateral wall of urinary bladder |
| B493. | Malignant neoplasm of anterior wall of urinary bladder |
| B494. | Malignant neoplasm of posterior wall of urinary bladder |
| B495. | Malignant neoplasm of bladder neck |
| B496. | Malignant neoplasm of ureteric orifice |
| B497. | Malignant neoplasm of urachus |
| B49y. | Malignant neoplasm of other site of urinary bladder |
| B49y0 | Malignant neoplasm, overlapping lesion of bladder |
| B49z. | Malignant neoplasm of urinary bladder NOS |
| B4A.. | Malig neop of kidney and other unspecified urinary organs |
| B4A.. | Renal malignant neoplasm |
| B4A0. | Malignant neoplasm of kidney parenchyma |
| B4A00 | Hypernephroma |
| B4A1. | Malignant neoplasm of renal pelvis |
| B4A10 | Malignant neoplasm of renal calyces |
| B4A11 | Malignant neoplasm of ureteropelvic junction |
| B4A1z | Malignant neoplasm of renal pelvis NOS |
| B4A2. | Malignant neoplasm of ureter |
| B4A3. | Malignant neoplasm of urethra |
| B4A4. | Malignant neoplasm of paraurethral glands |
| B4Ay. | Malignant neoplasm of other urinary organs |
| B4Ay0 | Malignant neoplasm of overlapping lesion of urinary organs |
| B4Az. | Malignant neoplasm of kidney or urinary organs NOS |
| B4y.. | Malignant neoplasm of genitourinary organ OS |
| B4z.. | Malignant neoplasm of genitourinary organ NOS |
| B5... | Malignant neoplasm of other and unspecified sites |
| B5... | Carcinoma of other and unspecified sites |
| B50.. | Malignant neoplasm of eye |
| B500. | Malig neop eyeball excl conjunctiva, cornea, retina, choroid |
| B5000 | Malignant neoplasm of ciliary body |
| B5001 | Malignant neoplasm of iris |
| B5002 | Malignant neoplasm of crystalline lens |
| B5003 | Malignant neoplasm of sclera |
| B500z | Malignant neoplasm of eyeball NOS |
| B501. | Malignant neoplasm of orbit |
| B5010 | Malignant neoplasm of connective tissue of orbit |
| B5011 | Malignant neoplasm of extraocular muscle of orbit |
| B501z | Malignant neoplasm of orbit NOS |
| B502. | Malignant neoplasm of lacrimal gland |
| B503. | Malignant neoplasm of conjunctiva |
| B504. | Malignant neoplasm of cornea |
| B505. | Malignant neoplasm of retina |
| B506. | Malignant neoplasm of choroid |
| B507. | Malignant neoplasm of lacrimal duct |
| B5070 | Malignant neoplasm of lacrimal sac |
| B5071 | Malignant neoplasm of nasolacrimal duct |
| B507z | Malignant neoplasm of lacrimal duct NOS |
| B508. | Malignant neoplasm, overlapping lesion of eye and adnexa |
| B50y. | Malignant neoplasm of other specified site of eye |
| B50z. | Malignant neoplasm of eye NOS |
| B51.. | Malignant neoplasm of brain |
| B51.. | Cerebral tumour - malignant |
| B510. | Malignant neoplasm cerebrum (excluding lobes and ventricles) |
| B5100 | Malignant neoplasm of basal ganglia |
| B5101 | Malignant neoplasm of cerebral cortex |
| B5102 | Malignant neoplasm of corpus striatum |
| B5103 | Malignant neoplasm of globus pallidus |
| B5104 | Malignant neoplasm of hypothalamus |
| B5105 | Malignant neoplasm of thalamus |
| B510z | Malignant neoplasm of cerebrum NOS |
| B511. | Malignant neoplasm of frontal lobe |
| B512. | Malignant neoplasm of temporal lobe |
| B5120 | Malignant neoplasm of hippocampus |
| B5121 | Malignant neoplasm of uncus |
| B512z | Malignant neoplasm of temporal lobe NOS |
| B513. | Malignant neoplasm of parietal lobe |
| B514. | Malignant neoplasm of occipital lobe |
| B515. | Malignant neoplasm of cerebral ventricles |
| B5150 | Malignant neoplasm of choroid plexus |
| B5151 | Malignant neoplasm of floor of cerebral ventricle |
| B515z | Malignant neoplasm of cerebral ventricle NOS |
| B516. | Malignant neoplasm of cerebellum |
| B517. | Malignant neoplasm of brain stem |
| B5170 | Malignant neoplasm of cerebral peduncle |
| B5171 | Malignant neoplasm of medulla oblongata |
| B5172 | Malignant neoplasm of midbrain |
| B5173 | Malignant neoplasm of pons |
| B517z | Malignant neoplasm of brain stem NOS |
| B51y. | Malignant neoplasm of other parts of brain |
| B51y0 | Malignant neoplasm of corpus callosum |
| B51y1 | Malignant neoplasm of tapetum |
| B51y2 | Malignant neoplasm, overlapping lesion of brain |
| B51yz | Malignant neoplasm of other part of brain NOS |
| B51z. | Malignant neoplasm of brain NOS |
| B52.. | Malig neop of other and unspecified parts of nervous system |
| B520. | Malignant neoplasm of cranial nerves |
| B5200 | Malignant neoplasm of olfactory bulb |
| B5201 | Malignant neoplasm of optic nerve |
| B5202 | Malignant neoplasm of acoustic nerve |
| B520z | Malignant neoplasm of cranial nerves NOS |
| B521. | Malignant neoplasm of cerebral meninges |
| B5210 | Malignant neoplasm of cerebral dura mater |
| B5211 | Malignant neoplasm of cerebral arachnoid mater |
| B5212 | Malignant neoplasm of cerebral pia mater |
| B521z | Malignant neoplasm of cerebral meninges NOS |
| B522. | Malignant neoplasm of spinal cord |
| B523. | Malignant neoplasm of spinal meninges |
| B5230 | Malignant neoplasm of spinal dura mater |
| B5231 | Malignant neoplasm of spinal arachnoid mater |
| B5232 | Malignant neoplasm of spinal pia mater |
| B523z | Malignant neoplasm of spinal meninges NOS |
| B524. | Malig neopl peripheral nerves and autonomic nervous system |
| B5240 | Malignant neoplasm of peripheral nerves of head, face & neck |
| B5241 | Malignant neoplasm of peripheral nerve,upp limb,incl should |
| B5242 | Malignant neoplasm of peripheral nerve of low limb, incl hip |
| B5243 | Malignant neoplasm of peripheral nerve of thorax |
| B5244 | Malignant neoplasm of peripheral nerve of abdomen |
| B5245 | Malignant neoplasm of peripheral nerve of pelvis |
| B5246 | Malignant neoplasm,overlap lesion periph nerve & auton ns |
| B524W | Mal neoplasm/periph nerves??? nervous system,unspc |
| B524X | Malignant neoplasm/peripheral nerves of trunk,unspecified |
| B525. | Malignant neoplasm of cauda equina |
| B52W. | Malig neopl, overlap lesion brain & other part of CNS |
| B52X. | Malignant neoplasm of meninges, unspecified |
| B52y. | Malignant neoplasm of other specified part of nervous system |
| B52z. | Malignant neoplasm of nervous system NOS |
| B53.. | Malignant neoplasm of thyroid gland |
| B54.. | Malig neop of other endocrine glands and related structures |
| B540. | Phaeochromocytoma |
| B540. | Malignant neoplasm of adrenal gland |
| B5400 | Malignant neoplasm of adrenal cortex |
| B5401 | Malignant neoplasm of adrenal medulla |
| B540z | Malignant neoplasm of adrenal gland NOS |
| B541. | Malignant neoplasm of parathyroid gland |
| B542. | Malignant neoplasm pituitary gland and craniopharyngeal duct |
| B5420 | Malignant neoplasm of pituitary gland |
| B5421 | Malignant neoplasm of craniopharyngeal duct |
| B542z | Malig neop pituitary gland or craniopharyngeal duct NOS |
| B543. | Malignant neoplasm of pineal gland |
| B544. | Malignant neoplasm of carotid body |
| B545. | Malignant neoplasm of aortic body and other paraganglia |
| B5450 | Malignant neoplasm of glomus jugulare |
| B5451 | Malignant neoplasm of aortic body |
| B5452 | Malignant neoplasm of coccygeal body |
| B545z | Malignant neoplasm of aortic body or paraganglia NOS |
| B54X. | Malignant neoplasm-pluriglandular involvement,unspecified |
| B54y. | Malignant neoplasm of other specified endocrine gland |
| B54z. | Malig neop of endocrine gland or related structure NOS |
| B55.. | Malignant neoplasm of other and ill-defined sites |
| B550. | Malignant neoplasm of head, neck and face |
| B5500 | Malignant neoplasm of head NOS |
| B5501 | Malignant neoplasm of cheek NOS |
| B5502 | Malignant neoplasm of nose NOS |
| B5503 | Malignant neoplasm of jaw NOS |
| B5504 | Malignant neoplasm of neck NOS |
| B5505 | Malignant neoplasm of supraclavicular fossa NOS |
| B550z | Malignant neoplasm of head, neck and face NOS |
| B551. | Malignant neoplasm of thorax |
| B5510 | Malignant neoplasm of axilla NOS |
| B5511 | Malignant neoplasm of chest wall NOS |
| B5512 | Malignant neoplasm of intrathoracic site NOS |
| B551z | Malignant neoplasm of thorax NOS |
| B552. | Malignant neoplasm of abdomen |
| B553. | Malignant neoplasm of pelvis |
| B5530 | Malignant neoplasm of inguinal region NOS |
| B5531 | Malignant neoplasm of presacral region |
| B5532 | Malignant neoplasm of sacrococcygeal region |
| B553z | Malignant neoplasm of pelvis NOS |
| B554. | Malignant neoplasm of upper limb NOS |
| B555. | Malignant neoplasm of lower limb NOS |
| B55y. | Malignant neoplasm of other specified sites |
| B55y0 | Malignant neoplasm of back NOS |
| B55y1 | Malignant neoplasm of trunk NOS |
| B55y2 | Malignant neoplasm of flank NOS |
| B55yz | Malignant neoplasm of specified site NOS |
| B55z. | Malignant neoplasm of other and ill defined site NOS |
| B59zX | Kaposi's sarcoma, unspecified |
| B6... | Malignant neoplasm of lymphatic and haemopoietic tissue |
| B6... | Malignant neoplasm of histiocytic tissue |
| B60.. | Lymphosarcoma and reticulosarcoma |
| B600. | Reticulosarcoma |
| B6000 | Reticulosarcoma of unspecified site |
| B6001 | Reticulosarcoma of lymph nodes of head, face and neck |
| B6002 | Reticulosarcoma of intrathoracic lymph nodes |
| B6003 | Reticulosarcoma of intra-abdominal lymph nodes |
| B6004 | Reticulosarcoma of lymph nodes of axilla and upper limb |
| B6005 | Reticulosarcoma of lymph nodes of inguinal region and leg |
| B6006 | Reticulosarcoma of intrapelvic lymph nodes |
| B6007 | Reticulosarcoma of spleen |
| B6008 | Reticulosarcoma of lymph nodes of multiple sites |
| B600z | Reticulosarcoma NOS |
| B601. | Lymphosarcoma |
| B6010 | Lymphosarcoma of unspecified site |
| B6011 | Lymphosarcoma of lymph nodes of head, face and neck |
| B6012 | Lymphosarcoma of intrathoracic lymph nodes |
| B6013 | Lymphosarcoma of intra-abdominal lymph nodes |
| B6014 | Lymphosarcoma of lymph nodes of axilla and upper limb |
| B6015 | Lymphosarcoma of lymph nodes of inguinal region and leg |
| B6016 | Lymphosarcoma of intrapelvic lymph nodes |
| B6017 | Lymphosarcoma of spleen |
| B6018 | Lymphosarcoma of lymph nodes of multiple sites |
| B601z | Lymphosarcoma NOS |
| B602. | Burkitt's lymphoma |
| B6020 | Burkitt's lymphoma of unspecified site |
| B6021 | Burkitt's lymphoma of lymph nodes of head, face and neck |
| B6022 | Burkitt's lymphoma of intrathoracic lymph nodes |
| B6023 | Burkitt's lymphoma of intra-abdominal lymph nodes |
| B6024 | Burkitt's lymphoma of lymph nodes of axilla and upper limb |
| B6025 | Burkitt's lymphoma of lymph nodes of inguinal region and leg |
| B6026 | Burkitt's lymphoma of intrapelvic lymph nodes |
| B6027 | Burkitt's lymphoma of spleen |
| B6028 | Burkitt's lymphoma of lymph nodes of multiple sites |
| B602z | Burkitt's lymphoma NOS |
| B60y. | Other specified reticulosarcoma or lymphosarcoma |
| B60z. | Reticulosarcoma or lymphosarcoma NOS |
| B61.. | Hodgkin's disease |
| B610. | Hodgkin's paragranuloma |
| B6100 | Hodgkin's paragranuloma of unspecified site |
| B6101 | Hodgkin's paragranuloma of lymph nodes of head, face, neck |
| B6102 | Hodgkin's paragranuloma of intrathoracic lymph nodes |
| B6103 | Hodgkin's paragranuloma of intra-abdominal lymph nodes |
| B6104 | Hodgkin's paragranuloma of lymph nodes of axilla and arm |
| B6105 | Hodgkin's paragranuloma lymph nodes inguinal region and leg |
| B6106 | Hodgkin's paragranuloma of intrapelvic lymph nodes |
| B6107 | Hodgkin's paragranuloma of spleen |
| B6108 | Hodgkin's paragranuloma of lymph nodes of multiple sites |
| B610z | Hodgkin's paragranuloma NOS |
| B611. | Hodgkin's granuloma |
| B6110 | Hodgkin's granuloma of unspecified site |
| B6111 | Hodgkin's granuloma of lymph nodes of head, face and neck |
| B6112 | Hodgkin's granuloma of intrathoracic lymph nodes |
| B6113 | Hodgkin's granuloma of intra-abdominal lymph nodes |
| B6114 | Hodgkin's granuloma of lymph nodes of axilla and upper limb |
| B6115 | Hodgkin's granuloma lymph nodes of inguinal region and leg |
| B6116 | Hodgkin's granuloma of intrapelvic lymph nodes |
| B6117 | Hodgkin's granuloma of spleen |
| B6118 | Hodgkin's granuloma of lymph nodes of multiple sites |
| B611z | Hodgkin's granuloma NOS |
| B612. | Hodgkin's sarcoma |
| B6120 | Hodgkin's sarcoma of unspecified site |
| B6121 | Hodgkin's sarcoma of lymph nodes of head, face and neck |
| B6122 | Hodgkin's sarcoma of intrathoracic lymph nodes |
| B6123 | Hodgkin's sarcoma of intra-abdominal lymph nodes |
| B6124 | Hodgkin's sarcoma of lymph nodes of axilla and upper limb |
| B6125 | Hodgkin's sarcoma of lymph nodes of inguinal region and leg |
| B6126 | Hodgkin's sarcoma of intrapelvic lymph nodes |
| B6127 | Hodgkin's sarcoma of spleen |
| B6128 | Hodgkin's sarcoma of lymph nodes of multiple sites |
| B612z | Hodgkin's sarcoma NOS |
| B613. | Hodgkin's disease, lymphocytic-histiocytic predominance |
| B6130 | Hodgkin's, lymphocytic-histiocytic predominance unspec site |
| B6131 | Hodgkin's, lymphocytic-histiocytic pred of head, face, neck |
| B6132 | Hodgkin's, lymphocytic-histiocytic pred intrathoracic nodes |
| B6133 | Hodgkin's, lymphocytic-histiocytic pred intra-abdominal node |
| B6134 | Hodgkin's, lymphocytic-histiocytic pred axilla and arm |
| B6135 | Hodgkin's, lymphocytic-histiocytic pred inguinal and leg |
| B6136 | Hodgkin's, lymphocytic-histiocytic pred intrapelvic nodes |
| B6137 | Hodgkin's, lymphocytic-histiocytic predominance of spleen |
| B6138 | Hodgkin's, lymphocytic-histiocytic pred of multiple sites |
| B613z | Hodgkin's, lymphocytic-histiocytic predominance NOS |
| B614. | Hodgkin's disease, nodular sclerosis |
| B6140 | Hodgkin's disease, nodular sclerosis of unspecified site |
| B6141 | Hodgkin's nodular sclerosis of head, face and neck |
| B6142 | Hodgkin's nodular sclerosis of intrathoracic lymph nodes |
| B6143 | Hodgkin's nodular sclerosis of intra-abdominal lymph nodes |
| B6144 | Hodgkin's nodular sclerosis of lymph nodes of axilla and arm |
| B6145 | Hodgkin's nodular sclerosis of inguinal region and leg |
| B6146 | Hodgkin's nodular sclerosis of intrapelvic lymph nodes |
| B6147 | Hodgkin's disease, nodular sclerosis of spleen |
| B6148 | Hodgkin's nodular sclerosis of lymph nodes of multiple sites |
| B614z | Hodgkin's disease, nodular sclerosis NOS |
| B615. | Hodgkin's disease, mixed cellularity |
| B6150 | Hodgkin's disease, mixed cellularity of unspecified site |
| B6151 | Hodgkin's mixed cellularity of lymph nodes head, face, neck |
| B6152 | Hodgkin's mixed cellularity of intrathoracic lymph nodes |
| B6153 | Hodgkin's mixed cellularity of intra-abdominal lymph nodes |
| B6154 | Hodgkin's mixed cellularity of lymph nodes of axilla and arm |
| B6155 | Hodgkin's mixed cellularity of lymph nodes inguinal and leg |
| B6156 | Hodgkin's mixed cellularity of intrapelvic lymph nodes |
| B6157 | Hodgkin's disease, mixed cellularity of spleen |
| B6158 | Hodgkin's mixed cellularity of lymph nodes of multiple sites |
| B615z | Hodgkin's disease, mixed cellularity NOS |
| B616. | Hodgkin's disease, lymphocytic depletion |
| B6160 | Hodgkin's lymphocytic depletion of unspecified site |
| B6161 | Hodgkin's lymphocytic depletion of head, face and neck |
| B6162 | Hodgkin's lymphocytic depletion of intrathoracic lymph nodes |
| B6163 | Hodgkin's lymphocytic depletion intra-abdominal lymph nodes |
| B6164 | Hodgkin's lymphocytic depletion lymph nodes axilla and arm |
| B6165 | Hodgkin's lymphocytic depletion lymph nodes inguinal and leg |
| B6166 | Hodgkin's lymphocytic depletion of intrapelvic lymph nodes |
| B6167 | Hodgkin's disease, lymphocytic depletion of spleen |
| B6168 | Hodgkin's lymphocytic depletion lymph nodes multiple sites |
| B616z | Hodgkin's disease, lymphocytic depletion NOS |
| B61z. | Hodgkin's disease NOS |
| B61z0 | Hodgkin's disease NOS, unspecified site |
| B61z1 | Hodgkin's disease NOS of lymph nodes of head, face and neck |
| B61z2 | Hodgkin's disease NOS of intrathoracic lymph nodes |
| B61z3 | Hodgkin's disease NOS of intra-abdominal lymph nodes |
| B61z4 | Hodgkin's disease NOS of lymph nodes of axilla and arm |
| B61z5 | Hodgkin's disease NOS of lymph nodes inguinal region and leg |
| B61z6 | Hodgkin's disease NOS of intrapelvic lymph nodes |
| B61z7 | Hodgkin's disease NOS of spleen |
| B61z8 | Hodgkin's disease NOS of lymph nodes of multiple sites |
| B61zz | Hodgkin's disease NOS |
| B62.. | Other malignant neoplasm of lymphoid and histiocytic tissue |
| B620. | Reticulosarcoma - follicular or nodular |
| B620. | Nodular lymphoma (Brill - Symmers disease) |
| B6200 | Nodular lymphoma of unspecified site |
| B6201 | Nodular lymphoma of lymph nodes of head, face and neck |
| B6202 | Nodular lymphoma of intrathoracic lymph nodes |
| B6203 | Nodular lymphoma of intra-abdominal lymph nodes |
| B6204 | Nodular lymphoma of lymph nodes of axilla and upper limb |
| B6205 | Nodular lymphoma of lymph nodes of inguinal region and leg |
| B6206 | Nodular lymphoma of intrapelvic lymph nodes |
| B6207 | Nodular lymphoma of spleen |
| B6208 | Nodular lymphoma of lymph nodes of multiple sites |
| B620z | Nodular lymphoma NOS |
| B621. | Mycosis fungoides |
| B6210 | Mycosis fungoides of unspecified site |
| B6211 | Mycosis fungoides of the lymph nodes of head, face and neck |
| B6212 | Mycosis fungoides of intrathoracic lymph nodes |
| B6213 | Mycosis fungoides of intra-abdominal lymph nodes |
| B6214 | Mycosis fungoides of lymph nodes of axilla and upper limb |
| B6215 | Mycosis fungoides of lymph nodes of inguinal region and leg |
| B6216 | Mycosis fungoides of intrapelvic lymph nodes |
| B6217 | Mycosis fungoides of spleen |
| B6218 | Mycosis fungoides of lymph nodes of multiple sites |
| B621z | Mycosis fungoides NOS |
| B622. | Sezary's disease |
| B6220 | Sezary's disease of unspecified site |
| B6221 | Sezary's disease of lymph nodes of head, face and neck |
| B6222 | Sezary's disease of intrathoracic lymph nodes |
| B6223 | Sezary's disease of intra-abdominal lymph nodes |
| B6224 | Sezary's disease of lymph nodes of axilla and upper limb |
| B6225 | Sezary's disease of lymph nodes of inguinal region and leg |
| B6226 | Sezary's disease of intrapelvic lymph nodes |
| B6227 | Sezary's disease of spleen |
| B6228 | Sezary's disease of lymph nodes of multiple sites |
| B622z | Sezary's disease NOS |
| B623. | Malignant histiocytosis |
| B6230 | Malignant histiocytosis of unspecified site |
| B6231 | Malignant histiocytosis of lymph nodes head, face and neck |
| B6232 | Malignant histiocytosis of intrathoracic lymph nodes |
| B6233 | Malignant histiocytosis of intra-abdominal lymph nodes |
| B6234 | Malignant histiocytosis of lymph nodes of axilla and arm |
| B6235 | Malignant histiocytosis of lymph nodes inguinal and leg |
| B6236 | Malignant histiocytosis of intrapelvic lymph nodes |
| B6237 | Malignant histiocytosis of spleen |
| B6238 | Malignant histiocytosis of lymph nodes of multiple sites |
| B623z | Malignant histiocytosis NOS |
| B624. | Leukaemic reticuloendotheliosis |
| B624. | Leukaemic reticuloendotheliosis |
| B6240 | Leukaemic reticuloendotheliosis of unspecified sites |
| B6241 | Leukaemic reticuloend of lymph nodes of head, face and neck |
| B6242 | Leukaemic reticuloendotheliosis of intrathoracic lymph nodes |
| B6243 | Leukaemic reticuloend of intra-abdominal lymph nodes |
| B6244 | Leukaemic reticuloend of lymph nodes of axilla and arm |
| B6245 | Leukaemic reticuloend of lymph nodes inguinal region and leg |
| B6246 | Leukaemic reticuloendotheliosis of intrapelvic lymph nodes |
| B6247 | Leukaemic reticuloendotheliosis of spleen |
| B6248 | Leukaemic reticuloend of lymph nodes of multiple sites |
| B624z | Leukaemic reticuloendotheliosis NOS |
| B625. | Letterer-Siwe disease |
| B625. | Histiocytosis X (acute, progressive) |
| B6250 | Letterer-Siwe disease of unspecified sites |
| B6251 | Letterer-Siwe disease of lymph nodes of head, face and neck |
| B6252 | Letterer-Siwe disease of intrathoracic lymph nodes |
| B6253 | Letterer-Siwe disease of intra-abdominal lymph nodes |
| B6254 | Letterer-Siwe disease of lymph nodes of axilla and arm |
| B6255 | Letterer-Siwe disease of lymph nodes inguinal region and leg |
| B6256 | Letterer-Siwe disease of intrapelvic lymph nodes |
| B6257 | Letterer-Siwe disease of spleen |
| B6258 | Letterer-Siwe disease of lymph nodes of multiple sites |
| B625z | Letterer-Siwe disease NOS |
| B626. | Malignant mast cell tumours |
| B6260 | Mast cell malignancy of unspecified site |
| B6261 | Mast cell malignancy of lymph nodes of head, face and neck |
| B6262 | Mast cell malignancy of intrathoracic lymph nodes |
| B6263 | Mast cell malignancy of intra-abdominal lymph nodes |
| B6264 | Mast cell malignancy of lymph nodes of axilla and upper limb |
| B6265 | Mast cell malignancy of lymph nodes inguinal region and leg |
| B6266 | Mast cell malignancy of intrapelvic lymph nodes |
| B6267 | Mast cell malignancy of spleen |
| B6268 | Mast cell malignancy of lymph nodes of multiple sites |
| B626z | Malignant mast cell tumour NOS |
| B627. | Non - Hodgkin's lymphoma |
| B6270 | Follicular non-Hodgkin's small cleaved cell lymphoma |
| B6271 | Follicular non-Hodg mixed sml cleavd & lge cell lymphoma |
| B6272 | Follicular non-Hodgkin's large cell lymphoma |
| B6273 | Diffuse non-Hodgkin's small cell (diffuse) lymphoma |
| B6274 | Diffuse non-Hodgkin's small cleaved cell (diffuse) lymphoma |
| B6275 | Diffuse non-Hodgkin mixed sml & lge cell (diffuse) lymphoma |
| B6276 | Diffuse non-Hodgkin's immunoblastic (diffuse) lymphoma |
| B6277 | Diffuse non-Hodgkin's lymphoblastic (diffuse) lymphoma |
| B6278 | Diffuse non-Hodgkin's lymphoma undifferentiated (diffuse) |
| B627C | Follicular lymphoma NOS |
| B627C | Follicular non-Hodgkin's lymphoma |
| B627D | Diffuse non-Hodgkin's centroblastic lymphoma |
| B627W | Unspecified B-cell non-Hodgkin's lymphoma |
| B627X | Diffuse non-Hodgkin's lymphoma, unspecified |
| B62x. | Malignant lymphoma otherwise specified |
| B62x0 | T-zone lymphoma |
| B62x1 | Lymphoepithelioid lymphoma |
| B62x2 | Peripheral T-cell lymphoma |
| B62x3 | Malignant reticuloendotheliosis |
| B62x4 | Malignant reticulosis |
| B62x5 | Malignant immunoproliferative small intestinal disease |
| B62x6 | True histiocytic lymphoma |
| B62xX | Oth and unspecif peripheral & cutaneous T-cell lymphomas |
| B62y. | Malignant lymphoma NOS |
| B62y0 | Malignant lymphoma NOS of unspecified site |
| B62y1 | Malignant lymphoma NOS of lymph nodes of head, face and neck |
| B62y2 | Malignant lymphoma NOS of intrathoracic lymph nodes |
| B62y3 | Malignant lymphoma NOS of intra-abdominal lymph nodes |
| B62y4 | Malignant lymphoma NOS of lymph nodes of axilla and arm |
| B62y5 | Malignant lymphoma NOS of lymph node inguinal region and leg |
| B62y6 | Malignant lymphoma NOS of intrapelvic lymph nodes |
| B62y7 | Malignant lymphoma NOS of spleen |
| B62y8 | Malignant lymphoma NOS of lymph nodes of multiple sites |
| B62yz | Malignant lymphoma NOS |
| B62z. | Malignant neoplasms of lymphoid and histiocytic tissue NOS |
| B62z0 | Unspec malig neop lymphoid/histiocytic of unspecified site |
| B62z1 | Unspec malig neop lymphoid/histiocytic lymph node head/neck |
| B62z2 | Unspec malig neop lymphoid/histiocytic of intrathoracic node |
| B62z3 | Unspec malig neop lymphoid/histiocytic intra-abdominal nodes |
| B62z4 | Unspec malig neop lymphoid/histiocytic lymph node axilla/arm |
| B62z5 | Unspec malig neop lymphoid/histiocytic nodes inguinal/leg |
| B62z6 | Unspec malig neop lymphoid/histiocytic of intrapelvic nodes |
| B62z7 | Unspec malig neop lymphoid/histiocytic of spleen |
| B62z8 | Unspec malig neop lymphoid/histiocytic of multiple sites |
| B62zz | Lymphoid and histiocytic malignancy NOS |
| B62zz | Immunoproliferative neoplasm |
| B63.. | Multiple myeloma and immunoproliferative neoplasms |
| B630. | Multiple myeloma |
| B630. | Myelomatosis |
| B630. | Kahler's disease |
| B6300 | Malignant plasma cell neoplasm, extramedullary plasmacytoma |
| B6301 | Solitary myeloma |
| B6302 | Plasmacytoma NOS |
| B6303 | Lambda light chain myeloma |
| B631. | Plasma cell leukaemia |
| B63y. | Other immunoproliferative neoplasms |
| B63z. | Immunoproliferative neoplasm or myeloma NOS |
| B64.. | Lymphoid leukaemia |
| B64.. | Lymphatic leukaemia |
| B640. | Acute lymphoid leukaemia |
| B641. | Chronic lymphoid leukaemia |
| B641. | Chronic lymphatic leukaemia |
| B642. | Subacute lymphoid leukaemia |
| B64y. | Other lymphoid leukaemia |
| B64y0 | Aleukaemic lymphoid leukaemia |
| B64y1 | Prolymphocytic leukaemia |
| B64y2 | Adult T-cell leukaemia |
| B64yz | Other lymphoid leukaemia NOS |
| B64z. | Lymphoid leukaemia NOS |
| B65.. | Myeloid leukaemia |
| B650. | Acute myeloid leukaemia |
| B651. | Chronic granulocytic leukaemia |
| B651. | Chronic myeloid leukaemia |
| B6510 | Chronic eosinophilic leukaemia |
| B6512 | Chronic neutrophilic leukaemia |
| B651z | Chronic myeloid leukaemia NOS |
| B652. | Subacute myeloid leukaemia |
| B653. | Myeloid sarcoma |
| B6530 | Chloroma |
| B6531 | Granulocytic sarcoma |
| B653z | Myeloid sarcoma NOS |
| B65y. | Other myeloid leukaemia |
| B65y0 | Aleukaemic myeloid leukaemia |
| B65y1 | Acute promyelocytic leukaemia |
| B65yz | Other myeloid leukaemia NOS |
| B65z. | Myeloid leukaemia NOS |
| B66.. | Monocytic leukaemia |
| B66.. | Monoblastic leukaemia |
| B66.. | Histiocytic leukaemia |
| B660. | Acute monocytic leukaemia |
| B661. | Chronic monocytic leukaemia |
| B662. | Subacute monocytic leukaemia |
| B66y. | Other monocytic leukaemia |
| B66y0 | Aleukaemic monocytic leukaemia |
| B66yz | Other monocytic leukaemia NOS |
| B66z. | Monocytic leukaemia NOS |
| B67.. | Other specified leukaemia |
| B670. | Di Guglielmo's disease |
| B670. | Acute erythraemia and erythroleukaemia |
| B671. | Heilmeyer - Schoner disease |
| B671. | Chronic erythraemia |
| B672. | Megakaryocytic leukaemia |
| B672. | Thrombocytic leukaemia |
| B673. | Mast cell leukaemia |
| B674. | Acute panmyelosis |
| B675. | Acute myelofibrosis |
| B67y. | Other and unspecified leukaemia |
| B67y0 | Lymphosarcoma cell leukaemia |
| B67yz | Other and unspecified leukaemia NOS |
| B67z. | Other specified leukaemia NOS |
| B68.. | Leukaemia of unspecified cell type |
| B680. | Acute leukaemia NOS |
| B681. | Chronic leukaemia NOS |
| B682. | Subacute leukaemia NOS |
| B68y. | Other leukaemia of unspecified cell type |
| B68z. | Leukaemia NOS |
| B69.. | Myelomonocytic leukaemia |
| B690. | Acute myelomonocytic leukaemia |
| B691. | Chronic myelomonocytic leukaemia |
| B692. | Subacute myelomonocytic leukaemia |
| B6y.. | Malignant neoplasm lymphatic or haematopoietic tissue OS |
| B6y0. | Myeloproliferative disease |
| B6y0. | Myeloproliferative disorder |
| B6y1. | Myelosclerosis with myeloid metaplasia |
| B6y1. | Megakaryocytic myelosclerosis |
| B6z.. | Malignant neoplasm lymphatic or haematopoietic tissue NOS |
| B6z0. | Kaposi's sarcoma of lymph nodes |
| By... | Neoplasms otherwise specified |
| Byu.. | [X]Additional neoplasm classification terms |
| Byu0. | [X]Malignant neoplasm of lip, oral cavity and pharynx |
| Byu1. | [X]Malignant neoplasm of digestive organs |
| Byu10 | [X]Other sarcomas of the liver |
| Byu11 | [X]Other specified carcinomas of liver |
| Byu12 | [X]Malignant neoplasm of intestinal tract, part unspecified |
| Byu13 | [X]Malignant neoplasm/ill-defined sites within digestive system |
| Byu2. | [X]Malignant neoplasm of respiratory and intrathoracic organs |
| Byu20 | [X]Malignant neoplasm of bronchus or lung, unspecified |
| Byu21 | [X]Malignant neoplasm/overlap lesion/heart, mediastinum |
| Byu22 | [X]Malignant neoplasm/upper respiratory tract, part unspecified |
| Byu23 | [X]Malignant neoplasm overlapping les/resp???? organs |
| Byu24 | [X]Malignant neoplasm/ill-defined sites within resp system |
| Byu25 | [X]Malignant neoplasm of mediastinum, part unspecified |
| Byu3. | [X]Malignant neoplasm of bone and articular cartilage |
| Byu30 | [X]Malignant neoplasm/overlap lesion/bone??? cartilage/limb |
| Byu31 | [X]Malignant neoplasm/bones??? cartilage/limb, unspecified |
| Byu32 | [X]Malignant neoplasm/overlap lesion/bone??? cartilage |
| Byu33 | [X]Malignant neoplasm/bone??? cartilage, unspecified |
| Byu4. | [X]Melanoma and other malignant neoplasms of skin |
| Byu40 | [X]Malignant melanoma of other???? parts of face |
| Byu41 | [X]Malignant melanoma of skin, unspecified |
| Byu42 | [X]Other malignant neoplasm/skin of other??? parts of face |
| Byu43 | [X]Malignant neoplasm of skin, unspecified |
| Byu5. | [X]Malignant neoplasm of mesothelial and soft tissue |
| Byu50 | [X]Mesothelioma of lung |
| Byu50 | [X]Mesothelioma of other sites |
| Byu51 | [X]Mesothelioma, unspecified |
| Byu52 | [X]Kaposi's sarcoma of multiple organs |
| Byu53 | [X]Kaposi's sarcoma, unspecified |
| Byu54 | [X]Malignant neoplasm/peripheral nerves of trunk, unspecified |
| Byu55 | [X]Malignant neoplasm/overlap lesion nervous system |
| Byu56 | [X]Malignant neoplasm/peripheral nervous system, unspecified |
| Byu57 | [X]Malignant neoplasm of peritoneum, unspecified |
| Byu58 | [X]Mal neoplasm/connective? tissue of trunk, unspecified |
| Byu59 | [X]Malignant neoplasm/connective soft tissue, unspecified |
| Byu5A | [X]Malignant neoplasm overlapping lesion of skin |
| Byu6. | [X]Malignant neoplasm of breast |
| Byu7. | [X]Malignant neoplasm of female genital organs |
| Byu70 | [X]Malignant neoplasm of uterine adnexa, unspecified |
| Byu71 | [X]Malignant neoplasm/other specified female genital organs |
| Byu72 | [X]Malignant neoplasm/overlapping lesion/female genital organs |
| Byu73 | [X]Malignant neoplasm of female genital organ, unspecified |
| Byu8. | [X]Malignant neoplasm of male genital organs |
| Byu80 | [X]Malignant neoplasm/other specified male genital organs |
| Byu81 | [X]Malignant neoplasm/overlapping lesion/male genital organs |
| Byu82 | [X]Malignant neoplasm of male genital organ, unspecified |
| Byu9. | [X]Malignant neoplasm of urinary tract |
| Byu90 | [X]Malignant neoplasm of urinary organ, unspecified |
| ByuA. | [X]Malignant neoplasm of eye, brain and other parts of cent |
| ByuA0 | [X]Malignant neoplasm/other and unspecified cranial nerves |
| ByuA1 | [X]Malignant neoplasm/central nervous system, unspecified |
| ByuA2 | [X]Malignant neoplasm of meninges, unspecified |
| ByuA3 | [X]Malignant neoplasm, overlap lesion brain & other part of CNS |
| ByuC0 | [X]Malignant neoplasm of other specified sites |
| ByuD. | [X]Malignant neoplasms of lymphoid, haematopoietic and rela |
| ByuD0 | [X]Other Hodgkin's disease |
| ByuD1 | [X]Other types of follicular non-Hodgkin's lymphoma |
| ByuD2 | [X]Other types of diffuse non-Hodgkin's lymphoma |
| ByuD3 | [X]Other specified types of non-Hodgkin's lymphoma |
| ByuD4 | [X]Other malignant immunoproliferative diseases |
| ByuD5 | [X]Other lymphoid leukaemia |
| ByuD6 | [X]Other myeloid leukaemia |
| ByuD7 | [X]Other monocytic leukaemia |
| ByuD8 | [X]Other specified leukaemias |
| ByuD9 | [X]Other leukaemia of unspecified cell type |
| ByuDA | [X]Other specified malignant neoplasm/lymphoid,haematopoietic? tissue |
| ByuDC | [X]Diffuse non-Hodgkin's lymphoma, unspecified |
| ByuDD | [X]Other and unspecified peripheral & cutaneous T-cell lymphomas |
| ByuDE | [X]Unspecified B-cell non-Hodgkin's lymphoma |
| ByuDF | [X]Non-Hodgkin's lymphoma, unspecified type |
| ByuDF | [X]Non-Hodgkin's lymphoma NOS |
| ByuE. | [X]Malignant neoplasms/independent (primary) multiple sites |
| ByuE0 | [X]Malignant neoplasms/independent(primary)multiple sites |
| Bz... | Neoplasms NOS |
| ZV10. | [V]Personal history of malignant neoplasm |
| ZV100 | [V]Personal history of malignant neoplasm of stomach |
| ZV100 | [V]Personal history of malignant neoplasm of gastrointestinal tract |
| ZV100 | [V]Personal history of malignant neoplasm of anus |
| ZV100 | [V]Personal history of malignant neoplasm of gastrointestinal tract |
| ZV100 | [V]Personal history of malignant neoplasm of oesophagus |
| ZV100 | [V]Personal history of malignant neoplasm of rectum |
| ZV100 | [V]Personal history of malignant neoplasm of tongue |
| ZV100 | [V]Personal history of malignant neoplasm of large intestine |
| ZV100 | [V]Personal history of malignant neoplasm of intestine |
| ZV100 | [V]Personal history of malignant neoplasm of liver |
| ZV101 | [V]Personal history of malignant neoplasm of lung |
| ZV101 | [V]Personal history of malignant neoplasm of bronchus |
| ZV101 | [V]Personal history of malignant neoplasm of trachea/bronchus/lung |
| ZV101 | [V]Personal history of malignant neoplasm of trachea |
| ZV102 | [V]Personal history of malignant neoplasm of nose |
| ZV102 | [V]Personal history of malignant neoplasm other intrathoracic organ |
| ZV102 | [V]Personal history of malignant neoplasm of larynx |
| ZV102 | [V]Personal history of malignant neoplasm - accessory sinus |
| ZV102 | [V]Personal history of malignant neoplasm of middle ear |
| ZV103 | [V]Personal history of malignant neoplasm of breast |
| ZV104 | [V]Personal history of malignant neoplasm of genital organ |
| ZV104 | [V]Personal history of malignant neoplasm of ovary |
| ZV104 | [V]Personal history of malignant neoplasm of cervix uteri |
| ZV104 | [V]Personal history of malignant neoplasm of testis |
| ZV104 | [V]Personal history of malignant neoplasm of genital organ |
| ZV104 | [V]Personal history of malignant neoplasm of uterine body |
| ZV104 | [V]Personal history malignant neoplasm - male genital organ |
| ZV104 | [V]Personal history of malignant neoplasm of prostate |
| ZV105 | [V]Personal history of malignant neoplasm of bladder |
| ZV105 | [V]Personal history of malignant neoplasm of kidney |
| ZV105 | [V]Personal history of malignant neoplasm of urinary organ |
| ZV105 | [V]Personal history of malignant neoplasm of kidney |
| ZV106 | [V]Personal history of leukaemia |
| ZV106 | [V]Personal history of myeloid leukaemia |
| ZV106 | [V]Personal history of monocytic leukaemia |
| ZV106 | [V]Personal history of lymphoid leukaemia |
| ZV107 | [V]Personal history of other haematopoietic neoplasm |
| ZV107 | [V]Personal history of reticulosarcoma |
| ZV107 | [V]Personal history other lymphatic/haematopoietic neoplasm |
| ZV107 | [V]Personal history of lymphosarcoma |
| ZV107 | [V]Personal history of Hodgkin's disease |
| ZV10y | [V]Personal history of malignant neoplasm of bone |
| ZV10y | [V]Personal history of other specified malignant neoplasm |
| ZV10y | [V]Personal history of malignant neoplasm of skin |
| ZV10y | [V]Personal history of malignant neoplasm of tongue |
| ZV10y | [V]Personal history of malignant neoplasm of eye |
| ZV10y | [V]Personal history of malignant neoplasm of brain |
| ZV10y | [V]Personal history of malignant neoplasm of thyroid |
| ZV10z | [V]Personal history of unspecified malignant neoplasm |

# Table S 3 List of ICD-10 codes used to identify and exclude cancer in SAIL

| **ICD-10 Code** | **Text description** |
| --- | --- |
| [C00-C75](http://apps.who.int/classifications/icd10/browse/2010/en#/C00-C14) | Malignant neoplasms, stated or presumed to be primary, of specified sites, except of lymphoid, haematopoietic and related tissue |
| C76 | Malignant neoplasm of other and ill-defined sites |
| C81 | Hodgkin lymphoma |
| C82 | Follicular lymphoma |
| C83 | Non-follicular lymphoma |
| C84 | Mature T/NK-cell lymphomas |
| C85 | Other and unspecified types of non-Hodgkin lymphoma |
| C883 | Immunoproliferative small intestinal disease |
| C887 | Other malignant immunoproliferative diseases |
| C889 | Malignant immunoproliferative disease, unspecified |
| C900 | Multiple myeloma |
| C901 | Plasma cell leukaemia |
| C91 | Lymphoid leukaemia |
| C92 | Myeloid leukaemia |
| C93 | Monocytic leukaemia |
| C940 | Acute erythroid leukaemia |
| C941 | Chronic erythraemia |
| C942 | Acute megakaryoblastic leukaemia |
| C943 | Mast cell leukaemia |
| C945 | Acute myelofibrosis |
| C947 | Other specified leukemias |
| C95 | Leukaemia of unspecified cell type |
| C96 | Other and unspecified malignant neoplasms of lymphoid, haematopoietic and related tissue |
| C80 | [Malignant neoplasm without specification of site](http://en.wikipedia.org/wiki/Malignant_neoplasm) |
| C880 | Waldenström's macroglobulinaemia |
| C881 | Alpha heavy chain disease |
| C882 | Gamma heavy chain disease |
| C902 | [Plasmacytoma, extramedullary](http://en.wikipedia.org/wiki/Plasmacytoma) |
| C944 | [Acute panmyelosis](http://en.wikipedia.org/wiki/Panmyelosis) |
| C97X | Malignant neoplasms of independent (primary) multiple sites |

# Table S 4 List of Read codes used to identify cerebrovascular disease as a covariate in SAIL

| **Read Code** | **Text Description** |
| --- | --- |
| 1477 . | H/O cerebrovascular disease |
| 14A7. | H/O CVA/Stroke |
| 14AB. | H/O TIA |
| 14AK. | H/O stroke in the last year |
| ZV125 | [V]Personal history of stroke |
| 7P242 | Delivery of rehabilitation for stroke |
| G6... | Cerebrovascular disease |
| G63.. | precerebral artery occulsion |
| G64.. | cerebral artery occulsion |
| G630. | basilar artery occulsion |
| G631. | carotid artery occulsion |
| G632. | vertebral artery occulsion |
| G633. | multiple and bilateral precerebral artery occulsion |
| G63y. | other precerebral artery occulsion |
| G63y0 | cerebral infarction due to thrombosis of precerebral arteries |
| G63y1 | cerebral infarction due to embolism of precerebral arteries |
| G64.. | Cerebral artery occulsion |
| G640. | cerebral thrombosis |
| G6400 | cerebral infarction due to thrombosis of cerebral arteries |
| G641. | cerebral embolism |
| G6410 | Cerebral infarction due to embolism of cerebral arteries |
| G64z. | Cerebral infarction |
| G64z0 | brainstem infarction |
| G64z1 | Wallenberg syndrome |
| G64z2 | Left sided cerebral infarction |
| G64z3 | Right sided cerebral infarction |
| G64z4 | infarction of the basal ganglia |
| G65.. | Transient cerebral ischaemia |
| G650. | Basilar artery syndrome |
| G651. | vertebral artery syndrome |
| G6510 | vertebro-basilar artery syndrome |
| G654. | multiple and bilateral precerebral artery syndrome |
| G65y. | other transient cerebral ischaemia |
| G65z. | Transient cerebral ischaemia NOS |
| G65z0 | impending cerebral ischaemia |
| G65z1 | intermittent cerebral ischaemia |
| G65zz | Transient cerebral ischaemia NOS |
| G66.. | Stroke or cerebrovascular accident unspecified |
| G660. | middle cerebral artery syndrome |
| G661. | anterior cerebral artery syndrome |
| G662. | posterior cerebral artery syndrome |
| G663. | brainstem stroke syndrome |
| G664. | cerebeller stroke syndrome |
| G665. | pure motor lacunar syndrome |
| G666. | pure sensory lacunar syndrome |
| G667. | Left sided CVA |
| G668. | Right sided CVA |
| G669. | cerebral paly , not congenital or infantile but acute |
| G67.. | other cerebrovasculare disease |
| G670. | cerebral atherosclerosis |
| G671. | Generalised ischaemic cerebrovascular disease NOS |
| G6710 | acute cerebrovascular insufficiency NOS |
| G6711 | chronic cerebrovascular ischaemia |
| G671z | generalised ischaemic CVD |
| G6W.. | Cerebral infarction due to unspecified occlusion or stenosis of precerebral arteries |
| G6X.. | Cerebral infarction due to unspecified occlusion or stenosis of cerebral arteries |
| G6y.. | Other specified cerebrovascular disease |
| G6z.. | Cerebrovascular disease NOS |
| G61.. | Stroke due to intracerebral haemorrhage |
| G610. | Cortical haemorrhage |
| G611. | Internal capsule haemorrhage |
| G612. | Basal nucleus haemorrhage |
| G613. | Cerebellar haemorrhage |
| G614. | Pontine haemorrhage |
| G615. | Bulbar haemorrhage |
| G616. | External capsule haemorrhage |
| G617. | Intracerebral haemorrhage, intraventricular |
| G618. | Intracerebral haemorrhage, multiple localized |
| G61X. | Intracerebral haemorrhage in hemisphere, unspecified |
| G61X0 | Left sided intracerebral haemorrhage, unspecified |
| G61X1 | Right sided intracerebral haemorrhage, unspecified |
| G68X. | Sequelae of stroke, not specified as haemorrhage or infarction |
| 8HHM. | Referral to multidisciplinary stroke function improvement service |
| G60.. | Subarachnoid haemorrhage |
| G600. | Ruptured berry aneurysm |
| G601. | Subarachnoid haemorrhage from carotid siphon and bifurcation |
| G602. | Subarachnoid haemorrhage from middle cerebral artery |
| G603. | Subarachnoid haemorrhage from anterior communicating artery |
| G604. | Subarachnoid haemorrhage from posterior communicating artery |
| G605. | Subarachnoid haemorrhage from basilar artery |
| G606. | Subarachnoid haemorrhage from vertebral artery |
| G60X. | Subarachnoid haemorrhage from intracranial artery, unspecified |
| G60z. | Subarachnoid haemorrhage NOS |
| G68.. | Late effects of cerebrovascular disease |
| G680. | Sequelae of subarachnoid haemorrhage |
| G681. | Sequelae of intracerebral haemorrhage |
| G682. | Sequelae of other nontraumatic intracranial haemorrhage |
| G683. | Sequelae of cerebral infarction |
| G68W. | Sequelae of other and unspecified cerebrovascular diseases |
| G68X. | Sequelae of stroke, not specified as haemorrhage or infarction |
| G6W.. | Cerebral infarction due to unspecified occlusion or stenosis of precerebral arteries |
| G6X.. | Cerebral infarction due to unspecified occlusion or stenosis of cerebral arteries |
| G6y.. | Other specified cerebrovascular disease |
| G6z.. | Cerebrovascular disease NOS |
| G677. | Occlusion and stenosis of cerebral arteries, not resulting in cerebral infarction |
| G6770 | Occlusion and stenosis of middle cerebral artery |
| G6771 | Occlusion and stenosis of anterior cerebral artery |
| G6772 | Occlusion and stenosis of posterior cerebral artery |
| G6773 | Occlusion and stenosis of cerebellar arteries |
| G6774 | Occlusion and stenosis of multiple and bilateral cerebral arteries |
| G678. | Cerebral autosomal dominant arteriopathy with subcortical infarcts and leukoencephalopathy |
| G679. | Small vessel cerebrovascular disease |
| G67y. | Other cerebrovascular disease OS |
| G67z. | Other cerebrovascular disease NOS |
| G61z. | Intracerebral haemorrhage NOS |
| G63z. | Precerebral artery occlusion NOS |
| 70043 | Evacuation of intracerebral haematoma NEC |
| Gyu6. | [X]Cerebrovascular diseases |
| Gyu67 | [X]Other specified cerebrovascular diseases |
| Gyu6D | [X]Sequelae/other  unspecified cerebrovascular diseases |
| G67y. | Other cerebrovascular disease OS |
| G67z. | Other cerebrovascular disease NOS |
| G68W. | Sequelae/other  unspecified cerebrovascular diseases |
| G680. | Sequelae of subarachnoid haemorrhage |
| G681. | Sequelae of intracerebral haemorrhage |
| 14A7. | H/O CVA/stroke |
| 662M. | Stroke monitoring |
| F11x2 | Cerebral degeneration due to cerebrovascular disease |
| G68.. | Late effects of cerebrovascular disease |
| G62z. | Intracranial haemorrhage NOS |
| Gyu60 | [X]Subarachnoid haemorrhage from other intracranial arteries |
| Gyu61 | [X]Other subarachnoid haemorrhage |
| Gyu62 | [X]Other intracerebral haemorrhage |
| Gyu65 | [X]Occlusion and stenosis of other precerebral arteries |
| Gyu66 | [X]Occlusion and stenosis of other cerebral arteries |
| Gyu6F | [X]Intracerebral haemorrhage in hemisphere, unspecified |

# Table S 5 List of Read codes used to identify hip fracture as a covariate in SAIL

| **Read Code** | **Text Description** |
| --- | --- |
| S30.. | hip fracture |
| S300. | Closed fracture proximal femur, transcervical |
| S3000 | Closed fracture proximal femur, intracapsular section, unspecified |
| S3001 | Closed fracture proximal femur, transepiphyseal |
| S3002 | Closed fracture proximal femur, midcervical section |
| S3003 | Closed fracture proximal femur, basicervical |
| S3004 | closed fractured head of femur |
| S3005 | Closed fracture proximal femur, subcapital, Garden grade unspecified |
| S3006 | Closed fracture proximal femur, subcapital, Garden grade I |
| S3007 | Closed fracture proximal femur, subcapital, Garden grade II |
| S3008 | Closed fracture proximal femur, subcapital, Garden grade III |
| S3009 | Closed fracture proximal femur, subcapital, Garden grade IV |
| S300A | Closed fracture of femur, upper epiphysis |
| S300y | Closed fracture proximal femur, other transcervical |
| S300z | Closed fracture proximal femur, transcervical, not otherwise specified |
| S301. | Open fracture proximal femur, transcervical |
| S3010 | Open fracture proximal femur, intracapsular section, unspecified |
| S3011 | Open fracture proximal femur, transepiphyseal |
| S3012 | Open fracture proximal femur, midcervical section |
| S3013 | Open fracture proximal femur, basicervical |
| S3014 | Open fracture head, femur |
| S3015 | Open fracture proximal femur,subcapital, Garden grade unspec |
| S3016 | Open fracture proximal femur,subcapital, Garden grade I |
| S3017 | Open fracture proximal femur,subcapital, Garden grade II |
| S3018 | Open fracture proximal femur,subcapital, Garden grade III |
| S3019 | Open fracture proximal femur,subcapital, Garden grade IV |
| S301A | Open fracture of femur, upper epiphysis |
| S301y | Open fracture proximal femur, other transcervical |
| S301z | Open fracture proximal femur, transcervical, not otherwise specified |
| S302. | Closed fracture of proximal femur, pertrochanteric |
| S3020 | Closed fracture of proximal femur, trochanteric section, unspecified |
| S3021 | Closed fracture proximal femur, intertrochanteric, two part |
| S3022 | Closed fracture proximal femur, subtrochanteric |
| S3023 | Closed fracture proximal femur, intertrochanteric, comminuted |
| S3024 | Closed fracture of femur, intertrochanteric |
| S302z | Closed fracture of proximal femur, pertrochanteric section, not otherwise specified |
| S303. | Open fracture of proximal femur, pertrochanteric |
| S3030 | Open fracture of proximal femur, trochanteric section, unspecified |
| S3031 | Open fracture proximal femur, intertrochanteric, two part |
| S3032 | Open fracture proximal femur, subtrochanteric |
| S3033 | Open fracture proximal femur, intertrochanteric, comminuted |
| S3034 | Open fracture of femur, intertrochanteric |
| S303z | Open fracture of proximal femur, pertrochanteric, not otherwise specified |
| S304. | Pertrochanteric fracture |
| S305. | Subtrochanteric fracture |
| S30w. | Closed fracture of unspecified proximal femur |
| S30x. | Open fracture of unspecified proximal femur |
| S30y. | Closed fracture of neck of femur NOS |
| S30z. | Open fracture of neck of femur NOS |
| S30y. | Hip fracture NOS |
| 14G7. | H/O: hip fracture |
| 7K1L4 | Closed reduction of fracture of hip |
| S4E.. | Fracture-dislocation or subluxation hip |
| S4E0. | Closed fracture-dislocation, hip joint |
| S4E1. | Open fracture-dislocation, hip joint |
| S4E2. | Closed fracture-subluxation, hip joint |
| S4E3. | Open fracture-subluxation, hip joint |

# Table S 6 List of Read codes used to identify ischaemic heart disease as a covariate in SAIL

| **Read Code** | **Text Description** |
| --- | --- |
| 14A3. | H/O: myocardial infarct <60 |
| 14A4. | H/O: myocardial infarct >60 |
| 14A5. | H/O: angina pectoris |
| 14AH. | H/O: Myocardial infarction in last year |
| 14AJ. | H/O: Angina in last year |
| 14AL. | H/O: Treatment for ischaemic heart disease |
| 14AT. | History of myocardial infarction |
| 792.. | Coronary artery operations |
| 7920 . | Saphenous vein graft replacement of coronary artery |
| 79200 | Saphenous vein graft replacement of one coronary artery |
| 79201 | Saphenous vein graft replacement of two coronary arteries |
| 79202 | Saphenous vein graft replacement of three coronary arteries |
| 79203 | Saphenous vein graft replacement of four+ coronary arteries |
| 7920y | Other specified saphenous vein graft replacement of coronary artery |
| 7920z | Saphenous vein graft replacement coronary artery NOS |
| 7921 . | Other autograft replacement of coronary artery |
| 79210 | Autograft replacement of one coronary artery NEC |
| 79211 | Autograft replacement of two coronary arteries NEC |
| 29212 | Autograft replacement of three coronary arteries NEC |
| 79213 | Autograft replacement of four or more coronary arteries NEC |
| 7921y | Other specified other autograft replacement of coronary artery |
| 7921z | Other autograft replacement of coronary artery NOS |
| 7922 . | Allograft replacement of coronary artery |
| 79220 | Allograft replacement of one coronary artery |
| 79221 | Allograft replacement of two coronary arteries |
| 79222 | Allograft replacement of three coronary arteries |
| 79223 | Allograft replacement of four or more coronary arteries |
| 7922y | Other specified allograft replacement of coronary artery |
| 7922z | Allograft replacement of coronary artery NOS |
| 7923 . | Prosthetic replacement of coronary artery |
| 79230 | Prosthetic replacement of one coronary artery |
| 79231 | Prosthetic replacement of two coronary arteries |
| 79232 | Prosthetic replacement of three coronary artery |
| 79233 | Prosthetic replacement of four or more coronary artery |
| 7923y | Other specified prosthetic replacement of coronary artery |
| 7923z | Prosthetic replacement of coronary artery NOS |
| 7924 . | Revision of bypass for coronary artery |
| 79240 | Revision of bypass for one coronary artery |
| 79241 | Revision of bypass for two coronary arteries |
| 79242 | Revision of bypass for three coronary arteries |
| 79243 | Revision of bypass for four or more coronary arteries |
| 79244 | Revision of connection of thoracic artery to coronary artery |
| 79245 | Revision of implantation of thoracic artery into heart |
| 7924y | Other specified revision of bypass for coronary artery |
| 7924z | Revision of bypass for coronary artery NOS |
| 7925 . | Connection of mammary artery to coronary artery |
| 7926 . | Connection of other thoracic artery to coronary artery |
| 7927 . | Other open operations on coronary artery |
| 7928 . | Transluminal balloon angioplasty of coronary artery |
| 7929 . | Other therapeutic transluminal operations on coronary artery |
| 792C. | Other replacement of coronary artery |
| 792D. | Other bypass of coronary artery |
| 793G. | Percutaneous transluminal balloon angioplasty and stenting of coronary artery |
| G3... | Ischaemic heart disease |
| G30.. | Acute myocardial infarction |
| G300. | Acute anterolateral infarction |
| G31.. | Other acute and subacute ischaemic heart disease |
| G310. | Postmyocardial infarction syndrome |
| G311. | Preinfarction syndrome |
| G3110 | Myocardial infarction aborted |
| G3111 | unstable angina |
| G3112 | angina at rest |
| G3113 | refractory angina |
| G3114 | worsening angina |
| G3115 | acute coronary syndrome |
| G311z | preinfarction synrdrome NOS |
| G312. | Coronary thrombosis not resulting in myocardial infarction |
| G31y. | Other acute and subacute ischaemic heart disease |
| G31y0 | Acute coronary insufficiency |
| G31y1 | microinfarction of heart |
| G31y2 | Subendocardial ischaemia |
| G31y3 | Transient myocardial ischaemia |
| G31yz | Other acute and subacute ischaemic heart disease NOS |
| G32.. | old myocardial infarction |
| G33.. | Angina pectoris |
| G330. | Angina decubitus |
| G3300 | Nocturnal angina |
| G330z | Angina decubitus NOS |
| G331. | Prinzmetal's angina |
| G332. | Coronary artery spasm |
| G33z. | Angina pectoris NOS |
| G33z0 | Status anginosus |
| G33z1 | Stenocardia |
| G33z2 | Syncope anginosa |
| G33z3 | Angina on effort |
| G33z4 | ischaemic chest pain |
| G33z5 | post infarct angina |
| G33z6 | new onset angina |
| G33z7 | Stable angina |
| G33zz | Angina pectoris NOS |
| G34.. | Other chronic ischaemic heart disease |
| G340. | Coronary atherosclerosis |
| G3400 | single coronary vessel disease |
| G3401 | double coronary vessel disease |
| G342. | Atherosclerotic cardiovascular disease |
| G343. | Ischaemic cardiomyopathy |
| G344. | silent myocardial ischaemia |
| G34y. | Other specified chronic ischaemic heart disease |
| G34y0 | Chronic coronary insufficiency |
| G34y1 | Chronic myocardial ischaemia |
| G34yz | Other specified chronic ischaemic heart disease NOS |
| G34z. | Other chronic ischaemic heart disease NOS |
| G34z0 | Asymptomatic coronary heart disease |
| G35.. | subsequent myocardial infarction |
| G350. | Subsequent myocardial infarction of anterior wall |
| G351. | subsequent myocardial infarction of inferior wall |
| G353. | subsequent myocardial infarction of other sites |
| G35X. | subsequent myocardial infarction of unspecified site |
| G36.. | Certain current complications following acute myocardial infarction |
| G360. | Haemopericardium as current complication following acute myocardial infarction |
| G361. | Atrial septal defect as current complication following acute myocardial infarction |
| G362. | Ventricular septal defect as current complication following acute myocardial infarction |
| G363. | Rupture of cardiac wall without haemopericardium as current complication following acute myocardial infarction |
| G364. | Rupture of chordae tendinae as current complication following acute myocardial infarction |
| G365. | Rupture of papillary muscle as current complication following acute myocardial infarction |
| G366. | Thrombosis of atrium, auricular appendage, and ventricle as current complications following acute myocardial infarction |
| G37.. | Cardiac syndrome X |
| G38.. | Postoperative myocardial infarction |
| G380. | Postoperative transmural myocardial infarction of anterior wall |
| G381. | Postoperative transmural myocardial infarction of inferior wall |
| G382. | Postoperative transmural myocardial infarction of other sites |
| G383. | Postoperative transmural myocardial infarction of unspecified site |
| G384. | Postoperative subendocardial myocardial infarction |
| G38z. | Postoperative myocardial infarction, unspecified |
| G3y.. | other specified ischaemic heart disease |
| G3z.. | ischaemic heart disease NOS |
| G301. | Other specified anterior myocardial infarction |
| G3010 | Acute anteroapical infarction |
| G3011 | Acute anteroseptal infarction |
| G301z | Anterior myocardial infarction NOS |
| G302. | Acute inferolateral infarction |
| G303. | Acute inferoposterior infarction |
| G304. | Posterior myocardial infarction NOS |
| G305. | Lateral myocardial infarction NOS |
| G306. | True posterior myocardial infarction |
| G306. | True posterior myocardial infarction |
| G307. | Acute subendocardial infarction |
| G3070 | Acute non-Q wave infarction |
| G3071 | Acute non-ST segment elevation myocardial infarction |
| G308. | Inferior myocardial infarction NOS |
| G309. | Acute Q-wave infarct |
| G30B. | Acute posterolateral myocardial infarction |
| G30X. | Acute transmural myocardial infarction of unspecified site |
| G30X0 | Acute ST segment elevation myocardial infarction |
| G30y. | Other acute myocardial infarction |
| G30y0 | Acute atrial infarction |
| G30y1 | Acute papillary muscle infarction |
| G30y2 | Acute septal infarction |
| G30yz | Other acute myocardial infarction NOS |
| G30z. | Acute myocardial infarction NOS |

# Table S 7 List of Read codes used to identify epilepsy as a covariate in SAIL

| **Read Code** | **Text Description** |
| --- | --- |
| F25.. | Epilepsy |
| F250. | Generalised nonconvulsive epilepsy |
| F2500 | Petit mal (minor) epilepsy |
| F2501 | Pykno-epilepsy |
| F2502 | Epileptic seizures - atonic |
| F2503 | Epileptic seizures - akinetic |
| F2504 | Juvenile absence epilepsy |
| F2505 | Lennox-Gastaut syndrome |
| F250y | Other specified generalised nonconvulsive epilepsy |
| F250z | Generalised nonconvulsive epilepsy NOS |
| F251. | Generalised convulsive epilepsy |
| F2510 | Grand mal (major) epilepsy |
| F2512 | Epileptic seizures - clonic |
| F2514 | epileptic seizures - tonic |
| F2513 | epileptic seizure myoclonic |
| F2515 | tonic clonic epilepsy |
| F2516 | Grand mal seizure |
| F251y | Other specified generalised convulsive epilepsy |
| F251z | Generalised convulsive epilepsy NOS |
| F252. | petit mal status |
| F253. | grand mal status |
| F254. | Partial epilepsy with impairment of consciousness |
| F2540 | Temporal lobe epilepsy |
| F2541 | Psychomotor epilepsy |
| F2542 | Psychosensory epilepsy |
| F2543 | Limbic system epilepsy |
| F2544 | epileptic automatism |
| F2545 | complex partial epileptic seizures |
| F254z | Partial epilepsy with impairment of consciousness NOS |
| F255. | Partial epilepsy without mention of impairment of consciousness |
| F2550 | Jacksonian, focal or motor epilepsy |
| F2551 | Sensory induced epilepsy |
| F2552 | Somatosensory epilepsy |
| F2553 | Visceral reflex epilepsy |
| F2554 | Visual reflex epilepsy |
| F2555 | Unilateral epilepsy |
| F2556 | simple partial epileptic seizure |
| F255y | Partial epilepsy without mention of impairment of consciousness OS |
| F255z | Partial epilepsy without mention of impairment of consciousness NOS |
| F25X. | Status epilepticus, unspecified |
| F25z. | Epilepsy NOS |
| 1473 . | history of epilepsy |
| 1O30 . | epilepsy confirmed |
| 1B1W. | Transient epileptic amnesia |
| F25H. | Generalised seizure |
| F25y. | Other forms of epilepsy |
| F25y1 | Gelastic epilepsy |
| F25y3 | Complex partial status epilepticus |
| F25yz | Other forms of epilepsy NOS |
| F25z. | Fit (in known epileptic) NOS |
| Fyu50 | [X]Other generalised epilepsy +epileptic syndrome |
| Fyu51 | [X]Other epilepsy |
| Fyu52 | [X]Other status epilepticus |
| Fyu59 | [X]Status epilepticus, unspecified |
| Eu05y | [X]Epileptic psychosis NOS |
| Eu060 | [X]Limbic epilepsy personality |
| Eu803 | [X]Acquired aphasia + epilepsy |
| F1321 | Progressive myoclonic epilepsy |
| 6674 | Epilepsy associated problems |
| 6677 | Epilepsy drug side effects |
| 6678 | Epilepsy treatment changed |
| 6679 | Epilepsy treatment started |
| 667A. | Epilepsy treatment stopped |
| 667B. | Nocturnal epilepsy |
| 667C. | Epilepsy control good |
| 667D. | Epilepsy control poor |
| 667E. | Epilepsy care arrangement |
| 667G. | Epilepsy restricts employment |
| 667H. | Epilepsy prevents employment |
| 667J. | Epilepsy impairs education |
| 667K. | Epilepsy limits activities |
| 667L. | Epilepsy does not limit activity |
| 667M. | Epilepsy management plan given |
| 667N. | Epilepsy severity |
| 667X. | No epilepsy drug side effects |
| 667Z. | Epilepsy monitoring NOS |
| 8BIF. | Epilepsy medication review |
| 1O30. | Epilepsy confirmed |
| 667.. | Epilepsy monitoring |

# Table S 8 List of Read codes used to identify parkinsonism as a covariate in SAIL

| **Read Code** | **Text Description** |
| --- | --- |
| F124. | Vascular parkinsonism |
| A94y1 | Syphilitic parkinsonism |
| F121. | Drug induced parkinsonism |
| F12.. | Parkinson's disease |
| F120. | Paralysis agitans |
| F123. | Postencephalitic parkinsonism |
| F12W. | Secondary parkinsonism due to other external agents |
| F12X. | Secondary parkinsonism, unspecified |
| F12z. | Parkinson's disease NOS |
| F13z. | Other and unspecified extrapyramidal diseases and abnormal movement disorders |
| F13zz | Extrapyramidal disease and abnormal movement disorder NOS |
| F11x9 | Cerebral degeneration in Parkinson's disease |
| 147F. | History of Parkinson's disease |

# Table S 9 List of Read codes used to identify atrial fibrillation as a covariate in SAIL

| **Read Code** | **Text Description** |
| --- | --- |
| 3272 . | ECG: atrial fibrillation |
| 14AN. | H/O: atrial fibrillation |
| 212R. | Atrial fibrillation resolved |
| G573. | Atrial fibrillation and flutter |
| G5730 | Atrial fibrillation |
| G5731 | Atrial flutter |
| G5732 | Paroxysmal atrial fibrillation |
| G5733 | Non-rheumatic atrial fibrillation |
| G5734 | Permanent atrial fibrillation |
| G5735 | Persistent atrial fibrillation |
| G573z | Atrial fibrillation and flutter NOS |
| 6A9.. | Atrial fibrillation annual review |
| 7936A | Implantation of intravenous pacemaker for atrial fibrillation |
| 3273 . | ECG: atrial flutter |

# Table S10 List of Read codes used to identify venous thromboembolic event as a covariate in SAIL

| **Read Code** | **Text Description** |
| --- | --- |
| SP321 | Thromboembolism after infusion |
| G401. | Pulmonary embolism |
| G4010 | Post operative pulmonary embolus |
| G4011 | Recurrent pulmonary embolism |
| 14A8. | H/O: embolism |
| 14A81 | H/O: Deep Vein Thrombosis |
| G641. | Cerebral embolism |
| G6410 | Cerebral infarction due to embolism of cerebral arteries |
| J420. | Mesenteric embolism |
| J4200 | Embolus of the superior mesenteric artery |
| J4201 | Thrombus of the superior mesenteric artery |
| J4202 | Thrombus of the superior mesenteric veins |
| J4203 | Acute ischaemic colitis |
| J420x | Mesenteric embolus NOS |
| J420y | Mesenteric thrombus NOS |
| J420z | Acute intestinal vascular insufficiency NOS |
| G82z0 | Embolism of vein NOS |
| K1380 | Renal artery embolism |
| K2750 | Corpus cavernosum embolism |
| SP07D | Embolism in vascular graft |
| G74.. | Arterial embolism and thrombosis |
| G740. | Embolism and thrombosis of the abdominal aorta |
| G741. | Embolism and thrombosis of the thoracic aorta |
| G742. | Embolism and thrombosis of an arm or leg artery |
| G7420 | Embolism and thrombosis of the brachial artery |
| G7421 | Embolism and thrombosis of the radial artery |
| G7422 | Embolism and thrombosis of the ulnar artery |
| G7423 | Embolism and thrombosis of the arm artery NOS |
| G7424 | Embolism and thrombosis of the femoral artery |
| G7425 | Embolism and thrombosis of the popliteal artery |
| G7426 | Embolism and thrombosis of the anterior tibial artery |
| G7427 | Embolism and thrombosis of the dorsalis pedis artery |
| G7428 | Embolism and thrombosis of the posterior tibial artery |
| G7429 | Embolism and thrombosis of the leg artery NOS |
| G74y. | Embolism and thrombosis of other specified artery |
| G74y0 | Embolism and/or thrombosis of the common iliac artery |
| G74y1 | Embolism and/or thrombosis of the internal iliac artery |
| G74y2 | Embolism and/or thrombosis of the external iliac artery |
| G74y3 | Embolism and thrombosis of the iliac artery unspecified |
| G74y5 | Embolism and thrombosis of the subclavian artery |
| G74y6 | Embolism and thrombosis of the splenic artery |
| G74y7 | Embolism and thrombosis of the axillary artery |
| G74y8 | Embolism and thrombosis of the coeliac artery |
| G74y9 | Embolism and thrombosis of the hepatic artery |
| G74yz | Embolism and thrombosis of other arteries NOS |
| G74z. | Arterial embolism and thrombosis NOS |
| G81.. | portal vein thrombosis |
| G82. | Other venous embolism and thrombosis |
| G820. | Budd - Chiari syndrome (hepatic vein thrombosis) |
| G821. | Thrombophlebitis migrans |
| G822. | Embolism and thrombosis of the vena cava |
| G8220 | Thrombosis of inferior vena cava |
| G823. | Embolism and thrombosis of the renal vein |
| G824. | Axillary vein thrombosis |
| G82y. | Other embolism and thrombosis |
| G82z. | Embolism and thrombosis NOS |
| G801. | Deep vein thrombosis |
| G8016 | Thrombophlebitis of the femoral vein |
| G8017 | Thrombophlebitis of the popliteal vein |
| G8018 | Thrombophlebitis of the anterior tibial vein |
| G8019 | Thrombophlebitis of the dorsalis pedis vein |
| G801A | Thrombophlebitis of the posterior tibial vein |
| G801B | Deep vein thrombophlebitis of the leg unspecified |
| G801D | Deep vein thrombosis of lower limb |
| G801F | Deep vein thrombosis of peroneal vein |
| G801G | Recurrent deep vein thrombosis |
| G801z | Deep vein phlebitis and thrombophlebitis of the leg NOS |
| SP122 | Post operative deep vein thrombosis |
| 8CMWA | On deep vein thrombosis care pathway |
| ZV128 | [V] Personal history DVT- deep vein thrombosis |

# Table S11 List of Read codes used to identify diabetes as a covariate in SAIL

| **Read Code** | **Text Description** |
| --- | --- |
| 1434 . | H/O: diabetes mellitus |
| 66Ao. | Diabetes type 2 review |
| 66An. | Diabetes type 1 review |
| C10E. | Type 1 diabetes mellitus |
| C10E0 | Type 1 diabetes mellitus with renal complications |
| C10E1 | Type 1 diabetes mellitus with ophthalmic complications |
| C10E2 | Type 1 diabetes mellitus with neurological complications |
| C10E3 | Type 1 diabetes mellitus with multiple complications |
| C10E4 | Unstable type 1 diabetes mellitus |
| C10E5 | Type 1 diabetes mellitus with ulcer |
| C10E6 | Type 1 diabetes mellitus with gangrene |
| C10E7 | Type 1 diabetes mellitus with retinopathy |
| C10E8 | Type 1 diabetes mellitus - poor control |
| C10E9 | Type 1 diabetes mellitus maturity onset |
| C10EA | Type 1 diabetes mellitus without complication |
| C10EB | Type 1 diabetes mellitus with mononeuropathy |
| C10EC | Type 1 diabetes mellitus with polyneuropathy |
| C10ED | Type 1 diabetes mellitus with nephropathy |
| C10EE | Type 1 diabetes mellitus with hypoglycaemic coma |
| C10EF | Type 1 diabetes mellitus with diabetic cataract |
| C10EG | Type 1 diabetes mellitus with peripheral angiopathy |
| C10EH | Type 1 diabetes mellitus with arthropathy |
| C10EJ | Type 1 diabetes mellitus with neuropathic arthropathy |
| C10EK | Type 1 diabetes mellitus with persistent proteinuria |
| C10EL | Type 1 diabetes mellitus with persistent microalbuminuria |
| C10EM | Type 1 diabetes mellitus with ketoacidosis |
| C10EN | Type 1 diabetes mellitus with ketoacidotic coma |
| C10EP | Type 1 diabetes mellitus with exudative maculopathy |
| C10EQ | Type 1 diabetes mellitus with gastroparesis |
| C10ER | Latent autoimmune diabetes mellitus in adult |
| C10F. | Type 2 diabetes mellitus |
| C10F0 | Type 2 diabetes mellitus with renal complications |
| C10F1 | Type 2 diabetes mellitus with ophthalmic complications |
| C10F2 | Type 2 diabetes mellitus with neurological complications |
| C10F3 | Type 2 diabetes mellitus with multiple complications |
| C10F4 | Type 2 diabetes mellitus with ulcer |
| C10F5 | Type 2 diabetes mellitus with gangrene |
| C10F6 | Type 2 diabetes mellitus with retinopathy |
| C10F7 | Type 2 diabetes mellitus - poor control |
| C10F8 | Reaven's syndrome |
| C10F9 | Type 2 diabetes mellitus without complication |
| C10FA | Type 2 diabetes mellitus with mononeuropathy |
| C10FB | Type 2 diabetes mellitus with polyneuropathy |
| C10FC | Type 2 diabetes mellitus with nephropathy |
| C10FD | Type 2 diabetes mellitus with hypoglycaemic coma |
| C10FE | Type 2 diabetes mellitus with diabetic cataract |
| C10FF | Type 2 diabetes mellitus with peripheral angiopathy |
| C10FG | Type 2 diabetes mellitus with arthropathy |
| C10FH | Type 2 diabetes mellitus with neuropathic arthropathy |
| C10FJ | Insulin treated Type 2 diabetes mellitus |
| C10FK | Hyperosmolar non-ketotic state in type 2 diabetes mellitus |
| C10FL | Type 2 diabetes mellitus with persistent proteinuria |
| C10FM | Type 2 diabetes mellitus with persistent microalbuminuria |
| C10FN | Type 2 diabetes mellitus with ketoacidosis |
| C10FP | Type 2 diabetes mellitus with ketoacidotic coma |
| C10FQ | Type 2 diabetes mellitus with exudative maculopathy |
| C10FR | Type 2 diabetes mellitus with gastroparesis |
| C10FS | Maternally inherited diabetes mellitus |
| 8CS0. | Diabetes care plan agreed |
| C10D. | Diabetes mellitus autosomal dominant type 2 |
| 14F4. | H/O: Admission in last year for diabetes foot problem |
| C10.. | Diabetes mellitus |
| C100. | Diabetes mellitus with no mention of complication |
| C1000 | Diabetes mellitus, juvenile type, with no mention of complication |
| C1001 | Diabetes mellitus, adult onset, with no mention of complication |
| C100z | Diabetes mellitus NOS with no mention of complication |
| C101. | Diabetes mellitus with ketoacidosis |
| C1010 | Diabetes mellitus, juvenile type, with ketoacidosis |
| C1010 | Diabetes mellitus, juvenile type, with ketoacidosis |
| C1011 | Diabetes mellitus, adult onset, with ketoacidosis |
| C101y | Other specified diabetes mellitus with ketoacidosis |
| C101z | Diabetes mellitus NOS with ketoacidosis |
| C102. | Diabetes mellitus with hyperosmolar coma |
| C1020 | Diabetes mellitus, juvenile type, with hyperosmolar coma |
| C1021 | Diabetes mellitus, adult onset, with hyperosmolar coma |
| C102z | Diabetes mellitus NOS with hyperosmolar coma |
| C103. | Diabetes mellitus with ketoacidotic coma |
| C1030 | Diabetes mellitus, juvenile type, with ketoacidotic coma |
| C1031 | Diabetes mellitus, adult onset, with ketoacidotic coma |
| C103y | Other specified diabetes mellitus with coma |
| C103z | Diabetes mellitus NOS with ketoacidotic coma |
| C104. | Diabetes mellitus with renal manifestation |
| C1040 | Diabetes mellitus, juvenile type, with renal manifestation |
| C1041 | Diabetes mellitus, adult onset, with renal manifestation |
| C104y | Other specified diabetes mellitus with renal complications |
| C104z | Diabetes mellitis with nephropathy NOS |
| C105. | Diabetes mellitus with ophthalmic manifestation |
| C1050 | Diabetes mellitus, juvenile type, with ophthalmic manifestation |
| C1051 | Diabetes mellitus, adult onset, with ophthalmic manifestation |
| C105y | Other specified diabetes mellitus with ophthalmic complications |
| C105z | Diabetes mellitus NOS with ophthalmic manifestation |
| C106. | Diabetes mellitus with neurological manifestation |
| C1060 | Diabetes mellitus, juvenile type, with neurological manifestation |
| C1061 | Diabetes mellitus, adult onset, with neurological manifestation |
| C106y | Other specified diabetes mellitus with neurological complications |
| C106z | Diabetes mellitus NOS with neurological manifestation |
| C107. | Diabetes mellitus with peripheral circulatory disorder |
| C1070 | Diabetes mellitus, juvenile type, with peripheral circulatory disorder |
| C1071 | Diabetes mellitus, adult onset, with peripheral circulatory disorder |
| C1072 | Diabetes mellitus, adult with gangrene |
| C1073 | IDDM with peripheral circulatory disorder |
| C1074 | NIDDM with peripheral circulatory disorder |
| C107y | Other specified diabetes mellitus with peripheral circulatory complications |
| C107z | Diabetes mellitus NOS with peripheral circulatory disorder |
| C108 | Insulin dependent diabetes mellitus |
| C1080 | Insulin-dependent diabetes mellitus with renal complications |
| C1081 | Insulin-dependent diabetes mellitus with ophthalmic complications |
| C1082 | Insulin-dependent diabetes mellitus with neurological complications |
| C1083 | Insulin dependent diabetes mellitus with multiple complications |
| C1084 | Unstable insulin dependent diabetes mellitus |
| C1085 | Insulin dependent diabetes mellitus with ulcer |
| C1086 | Insulin dependent diabetes mellitus with gangrene |
| C1087 | Insulin dependent diabetes mellitus with retinopathy |
| C1088 | Insulin dependent diabetes mellitus - poor control |
| C1089 | Insulin dependent diabetes maturity onset |
| C108A | Insulin-dependent diabetes without complication |
| C108B | Insulin dependent diabetes mellitus with mononeuropathy |
| C108C | Insulin dependent diabetes mellitus with polyneuropathy |
| C108D | Insulin dependent diabetes mellitus with nephropathy |
| C108E | Insulin dependent diabetes mellitus with hypoglycaemic coma |
| C108F | Insulin dependent diabetes mellitus with diabetic cataract |
| C108G | Insulin dependent diabetes mellitus with peripheral angiopathy |
| C108H | Insulin dependent diabetes mellitus with arthropathy |
| C108J | Insulin dependent diabetes mellitus with neuropathic arthropathy |
| C108y | Other specified diabetes mellitus with multiple complications |
| C108z | Unspecified diabetes mellitus with multiple complications |
| C109. | Non-insulin dependent diabetes mellitus |
| C1090 | Non Insulin-dependent diabetes mellitus with renal complications |
| C1091 | Non-Insulin-dependent diabetes mellitus with ophthalmic complications |
| C1092 | Non Insulin-dependent diabetes mellitus with neurological complications |
| C1093 | Non Insulin dependent diabetes mellitus with multiple complications |
| C1094 | Non Insulin dependent diabetes mellitus with ulcer |
| C1095 | Non Insulin dependent diabetes mellitus with gangrene |
| C1096 | Non Insulin dependent diabetes mellitus with retinopathy |
| C1097 | Non Insulin dependent diabetes mellitus - poor control |
| C1098 | Reaven's syndrome |
| C1099 | Non insulin-dependent diabetes without complication |
| C109A | Non Insulin dependent diabetes mellitus with mononeuropathy |
| C109B | Non Insulin dependent diabetes mellitus with polyneuropathy |
| C109C | Non Insulin dependent diabetes mellitus with nephropathy |
| C109D | Non Insulin dependent diabetes mellitus with hypoglycaemic coma |
| C109E | Non Insulin dependent diabetes mellitus with diabetic cataract |
| C109F | Non Insulin dependent diabetes mellitus with peripheral angiopathy |
| C109G | Non Insulin dependent diabetes mellitus with arthropathy |
| C109H | Non Insulin dependent diabetes mellitus with neuropathic arthropathy |
| C109J | Insulin treated Type 2 diabetes mellitus |
| C108z | Unspecified diabetes mellitus with multiple complications |
| C109K | Hyperosmolar non-ketotic state in type 2 diabetes mellitus |
| C10C. | Diabetes mellitus autosomal dominant |
| C10D. | Diabetes mellitus autosomal dominant type 2 |
| C10G. | Secondary pancreatic diabetes mellitus |
| C10G0 | Secondary pancreatic diabetes mellitus without complication |
| C10A. | Malnutrition-related diabetes mellitus |
| C10A0 | Malnutrition-related diabetes mellitus with coma |
| C10A1 | Malnutrition-related diabetes mellitus with ketoacidosis |
| C10A2 | Malnutrition-related diabetes mellitus with renal complications |
| C10A3 | Malnutrition-related diabetes mellitus with ophthalmic complications |
| C10A4 | Malnutrition-related diabetes mellitus with neurological complications |
| C10A5 | Malnutrition-related diabetes mellitus with peripheral circulatory complications |
| C10A6 | Malnutrition-related diabetes mellitus with multiple complications |
| C10A7 | Malnutrition-related diabetes mellitus with unspecified complications |
| C10AW | Malnutrition-related diabetes mellitus with unspecified complications |
| C10AX | Malnutrition-related diabetes mellitus with other specified complications |
| C10B. | Diabetes mellitus induced by steroids |
| C10B0 | Steroid induced diabetes mellitus without complication |
| C10H. | Diabetes mellitus induced by non-steroid drugs |
| C10H0 | Diabetes mellitus induced by non-steroid drugs without complication |
| C10M. | Lipoatrophic diabetes mellitus |
| C10M0 | Lipoatrophic diabetes mellitus without complication |
| C10N. | Secondary diabetes mellitus |
| C10N0 | Secondary diabetes mellitus without complication |
| C10y. | Diabetes mellitus with other specified manifestation |
| C10y0 | Diabetes mellitus, juvenile type, with other specified manifestation |
| C10y1 | Diabetes mellitus, adult onset, with other specified manifestation |
| C10yy | Other specified diabetes mellitus with other specified complications |
| C10yz | Diabetes mellitus NOS with other specified manifestation |
| C10z. | Diabetes mellitus with unspecified complication |
| C10z0 | Diabetes mellitus, juvenile type, with unspecified complication |
| C10z1 | Diabetes mellitus, adult onset, with unspecified complication |
| C10zy | Other specified diabetes mellitus with unspecified complications |
| C10zz | Diabetes mellitus NOS with unspecified complication |
| F4206 | Non proliferative diabetic retinopathy |
| R0543 | [D]Widespread diabetic foot gangrene |
| M2711 | Neuropathic diabetic ulcer - foot |
| G73y0 | Diabetic peripheral angiopathy |
| F3813 | Diabetic amyotrophy |
| F3y0. | Diabetic mononeuropathy |
| F35z0 | Diabetic mononeuritis NOS |
| M0372 | Cellulitis in diabetic foot |
| M2712 | Mixed diabetic ulcer - foot |
| M2710 | Ischaemic ulcer diabetic foot |
| G73y0 | Diabetic peripheral angiopathy |
| R0542 | [D]Gangrene of toe in diabetic |
| F3450 | Diabetic mononeuritis multiplex |
| 66AJ. | Unstable diabetes |
| 66AJ1 | Brittle diabetes |
| 66AJz | Diabetic - poor control NOS |
| 66A3. | Diabetic on diet only |
| 66A4. | Diabetic on oral treatment |
| 66A5. | Diabetic on insulin |
| 66A8. | Has seen dietician - diabetes |
| 66A9. | Understands diet - diabetes |
| 66AD. | Fundoscopy - diabetic check |
| 66AH. | Diabetic treatment changed |
| 66AH0 | Conversion to insulin |
| 66AH1 | Conversion to insulin in secondary care |
| 66AH2 | Conversion to insulin by diabetes specialist nurse |
| 66AI. | Diabetic - good control |
| 66AK. | Diabetic - cooperative patient |
| 66AL. | Diabetic-uncooperative patient |
| 66AM. | Diabetic - follow-up default |
| 66AN. | Date diabetic treatment start |
| 66AP. | Diabetes: practice programme |
| 66AQ. | Diabetes: shared care programme |
| 66AR. | Diabetes management plan given |
| 66AS. | Diabetic annual review |
| 66AT. | Annual diabetic blood test |
| 66AU. | Diabetes care by hospital only |
| 66AV. | Diabetic on insulin and oral treatment |
| 66AW. | Diabetic foot risk assessment |
| 66AY. | Diabetic diet - good compliance |
| 66AZ. | Diabetic monitoring NOS |
| 66Ab. | Diabetic foot examination |
| 66Ac. | Diabetic peripheral neuropathy screening |
| 66Ai. | Diabetic 6 month review |
| 66Ak. | Diabetic monitoring - lower risk albumin excretion |
| 66Al. | Diabetic monitoring - higher risk albumin excretion |
| 66Am. | Insulin dose changed |
| 66An. | Diabetes type 1 review |
| 66Ao. | Diabetes type 2 review |
| 66Ap. | Insulin treatment initiated |
| 66Aq. | Diabetic foot screen |
| 66As. | Diabetic on subcutaneous treatment |
| 66At. | Diabetic dietary review |
| 66Au. | Diabetic erectile dysfunction review |
| 66Av. | Diabetic assessment of erectile dysfunction |
| 66At0 | Type I diabetic dietary review |
| 66At1 | Type II diabetic dietary review |
| 66Aw. | Insulin dose |
| 8H2J. | Admit diabetic emergency |
| 8A12. | Diabetic crisis monitoring |
| 9N1v. | Seen in diabetic eye clinic |
| 9N1i. | Seen in diabetic foot clinic |
| 8HHy. | Referral to diabetic register |
| 13AC. | Diabetic weight reducing diet |
| 8H3O. | Non-urgent diabetic admission |
| 8HTk. | Referral to diabetic eye clinic |
| 2BBX. | O/E - left eye diabetic maculopathy |
| 8HBH. | Diabetic retinopathy 6 month review |
| 2BBW. | O/E - right eye diabetic maculopathy |
| 8HBG. | Diabetic retinopathy 12 month review |
| 9NND. | Under care of diabetic foot screener |
| 2BBQ. | O/E - left eye background diabetic retinopathy |
| 2BBP. | O/E - right eye background diabetic retinopathy |
| 2BBV. | O/E - left eye proliferative diabetic retinopathy |
| 2BBT. | O/E - right eye proliferative diabetic retinopathy |
| 2BBS. | O/E - left eye preproliferative diabetic retinopathy |
| 2BBR. | O/E - right eye preproliferative diabetic retinopathy |
| F4206 | Non proliferative diabetic retinopathy |

# Table S12 List of Read codes used to identify cerebrovascular disease as an outcome in SAIL

| **Read Code** | **Text Description** |
| --- | --- |
| G63.. | precerebral artery occlusion |
| G64.. | cerebral artery occlusion |
| G630. | basilar artery occlusion |
| G631. | carotid artery occlusion |
| G632. | vertebral artery occlusion |
| G633. | multiple and bilateral precerebral artery occlusion |
| G63y. | other precerebral artery occlusion |
| G63y0 | cerebral infarction due to thrombosis of precerebral arteries |
| G63y1 | cerebral infarction due to embolism of precerebral arteries |
| G64.. | Cerebral artery occlusion |
| G640. | cerebral thrombosis |
| G6400 | cerebral infarction due to thrombosis of cerebral arteries |
| G641. | cerebral embolism |
| G6410 | Cerebral infarction due to embolism of cerebral arteries |
| G64z. | Cerebral infarction |
| G64z0 | brainstem infarction |
| G64z1 | Wallenberg syndrome |
| G64z2 | Left sided cerebral infarction |
| G64z3 | right sided cerebral infarction |
| G64z4 | infarction of the basal ganglia |
| G65.. | Transient cerebral ischaemia |
| G650. | Basilar artery syndrome |
| G651. | vertebral artery syndrome |
| G6510 | vertebro-basilar artery syndrome |
| G654. | multiple and bilateral precerebral artery syndrome |
| G65y. | other transient cerebral ischaemia |
| G65z. | Transient cerebral ischaemia NOS |
| G65z0 | impending cerebral ischaemia |
| G65z1 | intermittent cerebral ischaemia |
| G65zz | Transient cerebral ischaemia NOS |
| G66.. | Stroke or cerebrovascular accident unspecified |
| G660. | middle cerebral artery syndrome |
| G661. | anterior cerebral artery syndrome |
| G662. | posterior cerebral artery syndrome |
| G663. | brainstem stroke syndrome |
| G664. | cerebeller stroke syndrome |
| G665. | pure motor lacunar syndrome |
| G666. | pure sensory lacunar syndrome |
| G667. | Left sided CVA |
| G668. | Right sided CVA |
| G669. | cerebral palsy , not congenital or infantile but acute |
| G6710 | acute cerebro vascular insufficiency NOS |
| G6W.. | Cerebral infarction due to unspecified occlusion or stenosis of precerebral arteries |
| G6X.. | Cerebral infarction due to unspecified occlusion or stenosis of cerebral arteries |
| G61.. | Stroke due to intracerebral haemorrhage |
| G610. | Cortical haemorrhage |
| G611. | Internal capsule haemorrhage |
| G612. | Basal nucleus haemorrhage |
| G613. | Cerebellar haemorrhage |
| G614. | Pontine haemorrhage |
| G615. | Bulbar haemorrhage |
| G616. | External capsule haemorrhage |
| G617. | Intracerebral haemorrhage, intraventricular |
| G618. | Intracerebral haemorrhage, multiple localized |
| G61X. | Intracerebral haemorrhage in hemisphere, unspecified |
| G61X0 | Left sided intracerebral haemorrhage, unspecified |
| G61X1 | Right sided intracerebral haemorrhage, unspecified |
| G60.. | Subarachnoid haemorrhage |
| G600. | Ruptured Berry aneurysm |
| G601. | Subarachnoid haemorrhage from carotid siphon and bifurcation |
| G602. | Subarachnoid haemorrhage from middle cerebral artery |
| G603. | Subarachnoid haemorrhage from anterior communicating artery |
| G604. | Subarachnoid haemorrhage from posterior communicating artery |
| G605. | Subarachnoid haemorrhage from basilar artery |
| G606. | Subarachnoid haemorrhage from vertebral artery |
| G60X. | Subarachnoid haemorrhage from intracranial artery, unspecified |
| G60z. | Subarachnoid haemorrhage NOS |
| G6W.. | Cerebral infarction due to unspecified occlusion or stenosis of precerebral arteries |
| G6X.. | Cerebral infarction due to unspecified occlusion or stenosis of cerebral arteries |
| G6770 | Occlusion and stenosis of middle cerebral artery |
| G6771 | Occlusion and stenosis of anterior cerebral artery |
| G6772 | Occlusion and stenosis of posterior cerebral artery |
| G6773 | Occlusion and stenosis of cerebellar arteries |
| G6774 | Occlusion and stenosis of multiple and bilateral cerebral arteries |
| G61z. | Intracerebral haemorrhage NOS |
| G62z. | Intracranial haemorrhage NOS |
| Gyu60 | [X]Subarachnoid haemorrhage from other intracranial arteries |
| Gyu61 | [X]Other subarachnoid haemorrhage |
| Gyu62 | [X]Other intracerebral haemorrhage |
| Gyu65 | [X]Occlusion and stenosis of other precerebral arteries |
| Gyu66 | [X]Occlusion and stenosis of other cerebral arteries |
| Gyu6F | [X]Intracerebral haemorrhage in hemisphere, unspecified |

# Table S13 List of ICD-10 codes used to identify cerebrovascular disease as an outcome in SAIL

| **ICD-10 Code** | **Description** |
| --- | --- |
| I63.0 | Cerebral infarction due to thrombosis of precerebral arteries |
| I63.1 | Cerebral infarction due to embolism of precerebral arteries |
| I63.2 | Cerebral infarction due to unspecified occlusion or stenosis of precerebral arteries |
| I63.3 | Cerebral infarction due to thrombosis of cerebral arteries |
| I63.4 | Cerebral infarction due to embolism of cerebral arteries |
| I63.5 | Cerebral infarction due to unspecified occlusion or stenosis of cerebral arteries |
| I63.6 | Cerebral infarction due to cerebral venous thrombosis, non-pyogenic |
| I63.8 | Other cerebral infarction |
| I63.9 | Cerebral infarction, unspecified |
| I64 | stroke not specified as haemorrhage or infarction |
| G45.0 | Vertebro-basilar artery syndrome |
| G45.1 | Carotid artery syndrome (hemispheric) |
| G45.2 | Multiple and bilateral precerebral artery syndromes |
| G45.3 | Amaurosis fugax |
| G45.4 | Transient global amnesia |
| G45.8 | Other transient cerebral ischaemic attacks and related syndromes |
| G45.9 | Transient cerebral ischaemic attack, unspecified |
| G46 | Vascular syndromes of brain in cerebrovascular diseases |
| G46.0 | Middle cerebral artery syndrome |
| G46.1 | Anterior cerebral artery syndrome |
| G46.2 | Posterior cerebral artery syndrome |
| G46.3 | Brain stem stroke syndrome |
| G46.4 | Cerebellar stroke syndrome |
| G46.5 | Pure motor lacunar syndrome |
| G46.6 | Pure sensory lacunar syndrome |
| G46.7 | Other lacunar syndrome |
| G46.8 | Other vascular syndromes of brain in cerebrovascular diseases |
| I60 | Subarachnoid haemorrhage |
| I60.0 | Subarachnoid haemorrhage from carotid siphon and bifurcation |
| I60.1 | Subarachnoid haemorrhage from middle cerebral artery |
| I60.2 | Subarachnoid haemorrhage from anterior communicating artery |
| I60.3 | Subarachnoid haemorrhage from posterior communicating artery |
| I60.4 | Subarachnoid haemorrhage from basilar artery |
| I60.5 | Subarachnoid haemorrhage from vertebral artery |
| I60.6 | Subarachnoid haemorrhage from other intracranial arteries |
| I60.7 | Subarachnoid haemorrhage from intracranial artery, unspecified |
| I60.8 | Other subarachnoid haemorrhage |
| I60.9 | Subarachnoid haemorrhage, unspecified |
| I61 | Intracerebral haemorrhage |
| I61.1 | Intracerebral haemorrhage in hemisphere, cortical |
| I61.2 | Intracerebral haemorrhage in hemisphere, unspecified |
| I61.3 | Intracerebral haemorrhage in brain stem |
| I61.4 | Intracerebral haemorrhage in cerebellum |
| I61.5 | Intracerebral haemorrhage, intraventricular |
| I61.6 | Intracerebral haemorrhage, multiple localized |
| I61.8 | Other intracerebral haemorrhage |
| I61.9 | Intracerebral haemorrhage, unspecified |

# Table S14 List of Read codes used to identify hip fracture as an outcome in SAIL

| **Read Code** | **Text Description** |
| --- | --- |
| S30.. | hip fracture |
| S300. | Closed fracture proximal femur, transcervical |
| S3000 | Closed fracture proximal femur, intracapsular section, unspecified |
| S3001 | Closed fracture proximal femur, transepiphyseal |
| S3002 | Closed fracture proximal femur, midcervical section |
| S3003 | Closed fracture proximal femur, basicervical |
| S3004 | closed fractured head of femur |
| S3005 | Closed fracture proximal femur, subcapital, Garden grade unspecified |
| S3006 | Closed fracture proximal femur, subcapital, Garden grade I |
| S3007 | Closed fracture proximal femur, subcapital, Garden grade II |
| S3008 | Closed fracture proximal femur, subcapital, Garden grade III |
| S3009 | Closed fracture proximal femur, subcapital, Garden grade IV |
| S300A | Closed fracture of femur, upper epiphysis |
| S300y | Closed fracture proximal femur, other transcervical |
| S300z | Closed fracture proximal femur, transcervical, not otherwise specified |
| S301. | Open fracture proximal femur, transcervical |
| S3010 | Open fracture proximal femur, intracapsular section, unspecified |
| S3011 | Open fracture proximal femur, transepiphyseal |
| S3012 | Open fracture proximal femur, midcervical section |
| S3013 | Open fracture proximal femur, basicervical |
| S3014 | Open fracture head, femur |
| S3015 | Open fracture proximal femur,subcapital, Garden grade unspecified |
| S3016 | Open fracture proximal femur,subcapital, Garden grade I |
| S3017 | Open fracture proximal femur,subcapital, Garden grade II |
| S3018 | Open fracture proximal femur,subcapital, Garden grade III |
| S3019 | Open fracture proximal femur,subcapital, Garden grade IV |
| S301A | Open fracture of femur, upper epiphysis |
| S301y | Open fracture proximal femur, other transcervical |
| S301z | Open fracture proximal femur, transcervical, not otherwise specified |
| S302. | Closed fracture of proximal femur, pertrochanteric |
| S3020 | Closed fracture of proximal femur, trochanteric section, unspecified |
| S3021 | Closed fracture proximal femur, intertrochanteric, two part |
| S3022 | Closed fracture proximal femur, subtrochanteric |
| S3023 | Closed fracture proximal femur, intertrochanteric, comminuted |
| S3024 | Closed fracture of femur, intertrochanteric |
| S302z | Closed fracture of proximal femur, pertrochanteric section, not otherwise specified |
| S303. | Open fracture of proximal femur, pertrochanteric |
| S3030 | Open fracture of proximal femur, trochanteric section, unspecified |
| S3031 | Open fracture proximal femur, intertrochanteric, two part |
| S3032 | Open fracture proximal femur, subtrochanteric |
| S3033 | Open fracture proximal femur, intertrochanteric, comminuted |
| S3034 | Open fracture of femur, intertrochanteric |
| S303z | Open fracture of proximal femur, pertrochanteric, not otherwise specified |
| S304. | Pertrochanteric fracture |
| S305. | Subtrochanteric fracture |
| S30w. | Closed fracture of unspecified proximal femur |
| S30x. | Open fracture of unspecified proximal femur |
| S30y. | Closed fracture of neck of femur NOS |
| S30z. | Open fracture of neck of femur NOS |
| 7K1L4 | Closed reduction of fracture of hip |
| S4E.. | Fracture-dislocation or subluxation hip |
| S4E0. | Closed fracture-dislocation, hip joint |
| S4E1. | Open fracture-dislocation, hip joint |
| S4E2. | Closed fracture-subluxation, hip joint |
| S4E3. | Open fracture-subluxation, hip joint |

# Table S15 List of ICD-10 codes used to identify hip fracture as an outcome in SAIL

| **ICD-10 Code** | **Text Description** |
| --- | --- |
| S72.0 | Fracture of neck of femur |
| S72.1 | Pertrochanteric fracture |
| S72.2 | Subtrochanteric fracture |

# Table S16 List of Read codes used to identify acute cardiac events as an outcome in SAIL

| **Read Code** | **Text Description** |
| --- | --- |
| G30.. | Acute myocardial infarction |
| G300. | Acute anterolateral infarction |
| G31y1 | microinfarction of heart |
| G31y2 | Subendocardial ischaemia |
| G31y3 | Transient myocardial ischaemia |
| G344. | silent myocardial ischaemia |
| G35.. | subsequent myocardial infarction |
| G350. | Subsequent myocardial infarction of anterior wall |
| G351. | subsequent myocardial infarction of inferior wall |
| G353. | subsequent myocardial infarction of other sites |
| G35X. | subsequent myocardial infarction of unspecified site |
| G36.. | Certain current complications following acute myocardial infarction |
| G360. | Haemopericardium as current complication following acute myocardial infarction |
| G361. | Atrial septal defect as current complication following acute myocardial infarction |
| G362. | Ventricular septal defect as current complication following acute myocardial infarction |
| G363. | Rupture of cardiac wall without haemopericardium as current complication following acute myocardial infarction |
| G364. | Rupture of chordae tendinae as current complication following acute myocardial infarction |
| G365. | Rupture of papillary muscle as current complication following acute myocardial infarction |
| G366. | Thrombosis of atrium, auricular appendage, and ventricle as current complications following acute myocardial infarction |
| G38.. | Postoperative myocardial infarction |
| G380. | Postoperative transmural myocardial infarction of anterior wall |
| G381. | Postoperative transmural myocardial infarction of inferior wall |
| G382. | Postoperative transmural myocardial infarction of other sites |
| G383. | Postoperative transmural myocardial infarction of unspecified site |
| G384. | Postoperative subendocardial myocardial infarction |
| G38z. | Postoperative myocardial infarction, unspecified |
| G301. | Other specified anterior myocardial infarction |
| G3010 | Acute anteroapical infarction |
| G3011 | Acute anteroseptal infarction |
| G301z | Anterior myocardial infarction NOS |
| G302. | Acute inferolateral infarction |
| G303. | Acute inferoposterior infarction |
| G304. | Posterior myocardial infarction NOS |
| G305. | Lateral myocardial infarction NOS |
| G306. | True posterior myocardial infarction |
| G306. | True posterior myocardial infarction |
| G307. | Acute subendocardial infarction |
| G3070 | Acute non-Q wave infarction |
| G3071 | Acute non-ST segment elevation myocardial infarction |
| G308. | Inferior myocardial infarction NOS |
| G309. | Acute Q-wave infarct |
| G30B. | Acute posterolateral myocardial infarction |
| G30X. | Acute transmural myocardial infarction of unspecified site |
| G30X0 | Acute ST segment elevation myocardial infarction |
| G30y. | Other acute myocardial infarction |
| G30y0 | Acute atrial infarction |
| G30y1 | Acute papillary muscle infarction |
| G30y2 | Acute septal infarction |
| G30yz | Other acute myocardial infarction NOS |
| G30z. | Acute myocardial infarction NOS |

# Table S17 List of ICD-10 codes used to identify acute cardiac events as an outcome in SAIL

| **ICD-10 Code** | **Text Description** |
| --- | --- |
| I21.0 | Acute transmural myocardial infarction of anterior wall |
| I21.1 | Acute transmural myocardial infarction of inferior wall |
| I21.2 | Acute transmural myocardial infarction of other sites |
| I21.3 | Acute transmural myocardial infarction of unspecified site |
| I21.4 | Acute subendocardial myocardial infarction |
| I21.9 | Acute myocardial infarction, unspecified |
| I22.0 | Subsequent myocardial infarction of anterior wall |
| I22.1 | Subsequent myocardial infarction of inferior wall |
| I22.8 | Subsequent myocardial infarction of other sites |
| I22.9 | Subsequent myocardial infarction of unspecified site |
| I23.0 | Haemopericardium as current complication following acute myocardial infarction |
| I23.1 | Atrial septal defect as current complication following acute myocardial infarction |
| I23.2 | Ventricular septal defect as current complication following acute myocardial infarction |
| I23.3 | Rupture of cardiac wall without haemopericardium as current complication following acute myocardial infarction |
| I23.4 | Rupture of chordae tendineae as current complication following acute myocardial inf |
| I23.5 | Rupture of papillary muscle as current complication following acute myocardial infarction |
| I23.6 | Thrombosis of atrium, auricular appendage, and ventricle as current complications following acute myocardial infarction |
| I23.8 | Other current complications following acute myocardial infarction |

# Table S18 List of Read codes used to identify DVT/PE as an outcome in SAIL

| **Read Code** | **Text Description** |
| --- | --- |
| G401. | Pulmonary embolism |
| G4010 | Post operative pulmonary embolus |
| G4011 | Recurrent pulmonary embolism |
| G801. | Deep vein thrombosis |
| G8016 | Thrombophlebitis of the femoral vein |
| G8017 | Thrombophlebitis of the popliteal vein |
| G8018 | Thrombophlebitis of the anterior tibial vein |
| G8019 | Thrombophlebitis of the dorsalis pedis vein |
| G801A | Thrombophlebitis of the posterior tibial vein |
| G801B | Deep vein thrombophlebitis of the leg unspecified |
| G801D | Deep vein thrombosis of lower limb |
| G801F | Deep vein thrombosis of peroneal vein |
| G801G | Recurrent deep vein thrombosis |
| G801z | Deep vein phlebitis and thrombophlebitis of the leg NOS |
| SP122 | Post operative deep vein thrombosis |

# Table S19 List of ICD-10 codes used to identify DVT/PE as an outcome in SAIL

| **ICD-10 Code** | **Text Description** |
| --- | --- |
| I26.0 | Pulmonary embolism with mention of acute cor pulmonale |
| I26.9 | Pulmonary embolism without mention of acute cor pulmonale |
| I80. | Phlebitis and thrombophlebitis |
| I80.1 | Phlebitis and thrombophlebitis of femoral vein |
| I80.2 | Phlebitis and thrombophlebitis of other deep vessels of lower extremities |
| I80.3 | Phlebitis and thrombophlebitis of lower extremities, unspecified |
| I80.8 | Phlebitis and thrombophlebitis of other sites |
| I80.9 | Phlebitis and thrombophlebitis of unspecified site |
| I81 | Portal vein thrombosis |
| I82 | Other venous embolism and thrombosis |
| I82.1 | Thrombophlebitis migrans |
| I82.2 | Embolism and thrombosis of vena cava |
| I82.3 | Embolism and thrombosis of renal vein |
| I82.8 | Embolism and thrombosis of other specified veins |
| I82.9 | Embolism and thrombosis of unspecified vein |

# Table S20 List of Read codes used to identify antipsychotic prescriptions in SAIL

| **Subtype** | **Read Code** | **Text Description** |
| --- | --- | --- |
| **All** | d4… | ANTIPSYCHOTIC DRUGS |
| **Sulpiride** | d4f.. | SULPIRIDE |
|  | d4f1. | DOLMATIL 200mg tablets |
|  | d4f2. | *SULPITIL 200mg tablets x28CP |
|  | d4f3. | *SULPITIL 200mg tablets x112CP |
|  | d4f4. | *SULPAREX 200mg tablets |
|  | d4f5. | DOLMATIL 400mg tablets |
|  | d4f6. | SULPOR 200mg/5mL oral solution |
|  | d4fw. | SULPIRIDE 200mg/5mL oral solution |
|  | d4fx. | SULPIRIDE 400mg tablets |
|  | d4fy. | SULPIRIDE 200mg/5mL sugar free solution |
|  | d4fz. | SULPIRIDE 200mg tablets |
| **Conventional** | d41.. | CHLORPROMAZINE HYDROCHLORIDE |
|  | d411. | CHLORPROMAZINE 10mg tablets |
|  | d412. | CHLORPROMAZINE 25mg tablets |
|  | d413. | CHLORPROMAZINE 50mg tablets |
|  | d414. | CHLORPROMAZINE 100mg tablets |
|  | d415. | CHLORPROMAZINE 25mg/5mL syrup |
|  | d416. | CHLORACTIL 25mg tablets |
|  | d417. | CHLORACTIL 50mg tablets |
|  | d418. | CHLORACTIL 100mg tablets |
|  | d419. | *DOZINE 25mg/5mL syrup |
|  | d41A. | CHLORPROMAZINE 25mg/5mL sugar free solution |
|  | d41B. | CHLORPROMAZINE 100mg/5mL sugar free solution |
|  | d41a. | *LARGACTIL 10mg tablets |
|  | d41b. | *LARGACTIL 25mg tablets |
|  | d41c. | *LARGACTIL 50mg tablets |
|  | d41d. | *LARGACTIL 100mg tablets |
|  | d41e. | *LARGACTIL 25mg/5mL syrup |
|  | d41f. | LARGACTIL FORTE 100mg/5mL syrup |
|  | d41g. | *LARGACTIL 25mg/mL injection |
|  | d41h. | LARGACTIL [CNS] 50mg/2mL injection |
|  | d41i. | *LARGACTIL 100mg suppositories |
|  | d41j. | CHLORPROMAZINE 100mg/5mL sugar free suspension |
|  | d41k. | CHLORPROMAZINE 100mg suppositories |
|  | d41l. | CHLORPROMAZINE 25mg/1mL injection |
|  | d41m. | CHLORPROMAZINE 50mg/2mL injection |
|  | d41o. | CHLORPROMAZINE 100mg/5mL syrup |
|  | d42.. | BENPERIDOL |
|  | d421. | ANQUIL 250micrograms tablets |
|  | d422. | *BENQUIL 250micrograms tablets |
|  | d42z. | BENPERIDOL 250microgram tablets |
|  | d43.. | *CHLORPROTHIXENE |
|  | d431. | *TARACTAN 15mg tablets |
|  | d432. | *TARACTAN 50mg tablets |
|  | d43y. | *CHLORPROTHIXENE 15mg tablets |
|  | d43z. | *CHLORPROTHIXENE 50mg tablets |
|  | d44.. | DROPERIDOL [CENTRAL NERVOUS SYSTEM USE] |
|  | d441. | *DROLEPTAN 10mg tablets |
|  | d442. | *DROLEPTAN 1mg/mL oral liquid |
|  | d443. | *DROLEPTAN 10mg/2mL injection |
|  | d444. | XOMOLIX 2.5mg/1mL solution for injection |
|  | d44w. | DROPERIDOL 2.5mg/1mL solution for injection |
|  | d44x. | *DROPERIDOL 10mg tablets |
|  | d44y. | *DROPERIDOL 1mg/mL oral liquid |
|  | d44z. | *DROPERIDOL 10mg/2mL injection |
|  | d45.. | FLUPENTIXOL [ANTIPSYCHOTIC] |
|  | d451. | DEPIXOL 3mg tablets |
|  | d45z. | FLUPENTIXOL 3mg tablets |
|  | d46.. | FLUPHENAZINE HYDROCHLORIDE |
|  | d461. | *MODITEN 1mg tablets |
|  | d462. | *MODITEN 2.5mg tablets |
|  | d463. | *MODITEN 5mg tablets |
|  | d46x. | FLUPHENAZINE HYDROCHLORIDE 1mg tablets |
|  | d46y. | FLUPHENAZINE HYDROCHLORIDE 2.5mg tablets |
|  | d46z. | FLUPHENAZINE HYDROCHLORIDE 5mg tablets |
|  | d47.. | HALOPERIDOL [ANTIPSYCHOTIC] |
|  | d471. | HALOPERIDOL 1.5mg tablets |
|  | d472. | HALOPERIDOL 5mg tablets |
|  | d473. | HALOPERIDOL 10mg tablets |
|  | d474. | HALOPERIDOL 20mg tablets |
|  | d475. | HALOPERIDOL 2mg/mL liquid |
|  | d476. | DOZIC 1mg/mL liquid |
|  | d477. | *DOZIC 2mg/mL liquid |
|  | d478. | FORTUNAN 500micrograms tablets |
|  | d479. | *FORTUNAN 1.5mg tablets |
|  | d47A. | HALOPERIDOL 2mg/5mL sugar free solution |
|  | d47B. | HALOPERIDOL 1mg/5mL sugar free solution |
|  | d47C. | KENTACE 1.5mg tablets |
|  | d47D. | KENTACE 5mg tablets |
|  | d47E. | KENTACE 10mg tablets |
|  | d47F. | KENTACE 20mg tablets |
|  | d47a. | *FORTUNAN 5mg tablets |
|  | d47b. | *FORTUNAN 10mg tablets |
|  | d47c. | *FORTUNAN 20mg tablets |
|  | d47d. | HALDOL 5mg tablets |
|  | d47e. | HALDOL 10mg tablets |
|  | d47f. | HALDOL 2mg/mL liquid |
|  | d47g. | *HALDOL 10mg/mL liquid |
|  | d47h. | HALDOL 5mg/1mL injection |
|  | d47i. | *HALDOL 10mg/2mL injection |
|  | d47j. | SERENACE 500micrograms capsules |
|  | d47k. | SERENACE 1.5mg tablets |
|  | d47l. | SERENACE 5mg tablets |
|  | d47m. | SERENACE 10mg tablets |
|  | d47n. | SERENACE 20mg tablets |
|  | d47o. | SERENACE 2mg/mL liquid 100mL |
|  | d47p. | SERENACE 5mg/1mL injection |
|  | d47q. | SERENACE 20mg/2mL injection |
|  | d47r. | HALOPERIDOL 500microgram capsules |
|  | d47s. | SERENACE 2mg/mL liquid 500mL |
|  | d47t. | HALOPERIDOL 1mg/mL liquid |
|  | d47u. | HALOPERIDOL 500micrograms tablets |
|  | d47v. | HALOPERIDOL 5mg/1mL injection |
|  | d47w. | HALOPERIDOL 10mg/2mL injection |
|  | d47x. | HALOPERIDOL 20mg/2mL injection |
|  | d47y. | HALOPERIDOL 10mg/mL oral solution |
|  | d48.. | LEVOMEPROMAZINE |
|  | d481. | NOZINAN 25mg/1mL injection |
|  | d482. | *VERACTIL 25mg tablets |
|  | d483. | NOZINAN 25mg tablets |
|  | d48y. | LEVOMEPROMAZINE 25mg/1mL injection |
|  | d48z. | LEVOMEPROMAZINE 25mg tablets |
|  | d49.. | OXYPERTINE |
|  | d491. | *INTEGRIN 10mg capsules |
|  | d492. | *INTEGRIN 40mg tablets |
|  | d49y. | *OXYPERTINE 10mg capsules |
|  | d49z. | *OXYPERTINE 40mg tablets |
|  | d4a.. | PERICYAZINE |
|  | d4a1. | *NEULACTIL 2.5mg tablets |
|  | d4a2. | *NEULACTIL 10mg tablets |
|  | d4a3. | *NEULACTIL 25mg tablets |
|  | d4a4. | *NEULACTIL FORTE 10mg/5mL syrp |
|  | d4aw. | PERICYAZINE 2.5mg tablets |
|  | d4ax. | PERICYAZINE 10mg tablets |
|  | d4ay. | *PERICYAZINE 25mg tablets |
|  | d4az. | PERICYAZINE 10mg/5mL syrup |
|  | d4b.. | PERPHENAZINE [CENTRAL NERVOUS SYSTEM USE] |
|  | d4b1. | FENTAZIN 2mg tablets |
|  | d4b2. | FENTAZIN 4mg tablets |
|  | d4b3. | *FENTAZIN 8mg tablets |
|  | d4b4. | *FENTAZIN 5mg/1mL injection |
|  | d4b5. | PERPHENAZINE 2mg/5mL sugar free solution |
|  | d4b6. | PERPHENAZINE 4mg/5mL sugar free solution |
|  | d4bx. | PERPHENAZINE 2mg tablets |
|  | d4by. | PERPHENAZINE 4mg tablets |
|  | d4bz. | *PERPHENAZINE 8mg tablets |
|  | d4c.. | PIMOZIDE |
|  | d4c1. | *ORAP 2mg tablets |
|  | d4c2. | ORAP 4mg tablets |
|  | d4c3. | *ORAP 10mg tablets |
|  | d4cx. | *PIMOZIDE 2mg tablets |
|  | d4cy. | PIMOZIDE 4mg tablets |
|  | d4cz. | *PIMOZIDE 10mg tablets |
|  | d4d.. | PROCHLORPERAZINE [antipsych] [see dhe..] |
|  | d4e.. | PROMAZINE HYDROCHLORIDE |
|  | d4e1. | *SPARINE 50mg/5mL suspension |
|  | d4e2. | *SPARINE 50mg/1mL injection |
|  | d4e3. | *SPARINE 100mg/2mL injection |
|  | d4e4. | PROMAZINE 25mg tablets |
|  | d4e5. | PROMAZINE 50mg tablets |
|  | d4ev. | PROMAZINE 25mg/5mL syrup |
|  | d4ew. | PROMAZINE 50mg/5mL syrup |
|  | d4ex. | *PROMAZINE 50mg/5mL suspension |
|  | d4ey. | PROMAZINE 50mg/1mL injection |
|  | d4ez. | *PROMAZINE 100mg/2mL injection |
|  | d4g.. | THIORIDAZINE |
|  | d4g1. | *MELLERIL 10mg tablets |
|  | d4g2. | *MELLERIL 25mg tablets |
|  | d4g3. | *MELLERIL 50mg tablets |
|  | d4g4. | *MELLERIL 100mg tablets |
|  | d4g5. | *MELLERIL 25mg/5mL suspension |
|  | d4g6. | MELLERIL 100mg/5mL oral suspension |
|  | d4g7. | MELLERIL 25mg/5mL orange syrup |
|  | d4gp. | THIORIDAZINE 10mg/5mL syrup |
|  | d4gq. | THIORIDAZINE 25mg/5mL sugar free solution |
|  | d4gr. | THIORIDAZINE 50mg/5mL sugar free solution |
|  | d4gs. | THIORIDAZINE 100mg/5mL sugar free solution |
|  | d4gt. | *THIORIDAZINE 10mg tablets |
|  | d4gu. | THIORIDAZINE 25mg tablets |
|  | d4gv. | THIORIDAZINE 50mg tablets |
|  | d4gw. | THIORIDAZINE 100mg tablets |
|  | d4gx. | THIORIDAZINE 25mg/5mL suspension |
|  | d4gy. | THIORIDAZINE 100mg/5mL oral suspension |
|  | d4gz. | *THIORIDAZINE 25mg/5mL syrup |
|  | d4h.. | TRIFLUOPERAZINE [ANTIPSYCHOTIC] |
|  | d4h1. | STELAZINE 1mg tablets |
|  | d4h2. | STELAZINE 5mg tablets |
|  | d4h3. | *STELAZINE 2mg m/r capsules |
|  | d4h4. | *STELAZINE 10mg m/r capsules |
|  | d4h5. | *STELAZINE 15mg m/r capsules |
|  | d4h6. | STELAZINE 1mg/5mL syrup |
|  | d4h7. | STELAZINE CONCENTRATE 10mg/mL liquid |
|  | d4h8. | *STELAZINE 1mg/1mL injection |
|  | d4h9. | TRIFLUOPERAZINE 5mg/5mL sugar free syrup |
|  | d4hA. | STELAZINE FORTE 5mg/5mL sugar free oral suspension |
|  | d4hr. | TRIFLUOPERAZINE 5mg/5mL sugar free oral suspension |
|  | d4hs. | TRIFLUOPERAZINE 1mg tablets |
|  | d4ht. | TRIFLUOPERAZINE 5mg tablets |
|  | d4hu. | *TRIFLUOPERAZINE 2mg m/r caps |
|  | d4hv. | *TRIFLUOPERAZINE 10mg m/r caps |
|  | d4hw. | *TRIFLUOPERAZINE 15mg m/r caps |
|  | d4hx. | TRIFLUOPERAZINE 1mg/5mL syrup |
|  | d4hy. | TRIFLUOPERAZINE 10mg/mL liquid |
|  | d4hz. | TRIFLUOPERAZINE 1mg/1mL injection |
|  | d4i1. | TRIFLUPERIDOL |
|  | d4i2. | TRIPERIDOL 500micrograms tablets |
|  | d4iy. | *TRIPERIDOL 1mg tablets |
|  | d4iz. | TRIFLUPERIDOL 500microgram tablets |
|  | d4l.. | *TRIFLUPERIDOL 1mg tablets |
|  | d4j.. | ZUCLOPENTHIXOL DIHYDROCHLORIDE |
|  | d4j1. | CLOPIXOL 2mg tablets |
|  | d4j2. | CLOPIXOL 10mg tablets |
|  | d4j3. | CLOPIXOL 25mg tablets |
|  | d4jx. | ZUCLOPENTHIXOL DIHYDROCHLORIDE 2mg tablets |
|  | d4jy. | ZUCLOPENTHIXOL DIHYDROCHLORIDE 10mg tablets |
|  | d4jz. | ZUCLOPENTHIXOL DIHYDROCHLORIDE 25mg tablets |
|  | d4k.. | LOXAPINE SUCCINATE |
|  | d4k1. | *LOXAPINE 10mg capsules |
|  | d4k2. | *LOXAPINE 25mg capsules |
|  | d4k3. | *LOXAPINE 50mg capsules |
|  | d4k4. | *LOXAPAC 10mg capsules |
|  | d4k5. | *LOXAPAC 25mg capsules |
|  | d4k6. | *LOXAPAC 50mg capsules |
|  | d4n.. | ZUCLOPENTHIXOL ACETATE |
|  | d4n1. | CLOPIXOL ACUPHASE 50mg/1mL injection (oily) |
|  | d4n2. | CLOPIXOL ACUPHASE 100mg/2mL injection (oily) |
|  | d4n3. | ZUCLOPENTHIXOL ACETATE 50mg/1mL injection (oily) |
|  | d4n4. | ZUCLOPENTHIXOL ACETATE 100mg/2mL injection (oily) |
|  | d5... | ANTIPSYCHOTIC DEPOT INJECTIONS |
|  | d51.. | FLUPENTIXOL DECANOATE |
|  | d511. | DEPIXOL 20mg/1mL injection |
|  | d512. | *DEPIXOL 20mg/1mL syringe |
|  | d513. | DEPIXOL 40mg/2mL injection |
|  | d514. | *DEPIXOL 40mg/2mL syringe |
|  | d515. | *DEPIXOL 200mg/10mL injection |
|  | d516. | DEPIXOL CONC. 100mg/1mL injection |
|  | d517. | DEPIXOL CONC. 500mg/5mL injection |
|  | d518. | DEPIXOL CONC. 50mg/0.5mL injection |
|  | d519. | FLUPENTIXOL 50mg/0.5mL injection |
|  | d51a. | DEPIXOL LOW VOLUME 200mg/1mL intramuscular injection |
|  | d51s. | FLUPENTHIXOL DECANOATE 20mg/1mL prefilled syringe |
|  | d51t. | FLUPENTHIXOL DECANOATE 40mg/2mL prefilled syringe |
|  | d51u. | FLUPENTIXOL DECANOATE 200mg/1mL intramuscular injection |
|  | d51v. | FLUPENTIXOL DECANOATE 20mg/1mL injection |
|  | d51w. | FLUPENTIXOL DECANOATE 40mg/2mL injection |
|  | d51x. | FLUPENTHIXOL DECANOATE 200mg/10mL injection |
|  | d51y. | FLUPENTIXOL DECANOATE 100mg/1mL injection |
|  | d51z. | FLUPENTHIXOL DECANOATE 500mg/5mL injection |
|  | d52.. | FLUPHENAZINE DECANOATE |
|  | d521. | MODECATE 12.5mg/0.5mL injection |
|  | d522. | MODECATE 25mg/1mL injection |
|  | d523. | *MODECATE 25mg/1mL syringe |
|  | d524. | MODECATE 50mg/2mL injection |
|  | d525. | *MODECATE 50mg/2mL syringe |
|  | d526. | *MODECATE 250mg/10mL injection |
|  | d527. | MODECATE CONCENTRATE 50mg/0.5mL injection |
|  | d528. | MODECATE CONCENTRATE 100mg/1mL injection |
|  | d529. | FLUPHENAZINE DECANOATE 50mg/0.5mL injection |
|  | d52A. | *DECAZATE 25mg/1mL injection |
|  | d52B. | *DECAZATE 50mg/0.5mL injection |
|  | d52C. | *DECAZATE 100mg/1mL injection |
|  | d52a. | FLUPHENAZINE DECANOATE 100mg/1mL injection |
|  | d52s. | FLUPHENAZINE DECANOATE 25mg/1mL prefilled syringe |
|  | d52t. | FLUPHENAZINE DECANOATE 50mg/2mL prefilled syringe |
|  | d52u. | FLUPHENAZINE DECANOATE 12.5mg/0.5mL injection |
|  | d52v. | FLUPHENAZINE DECANOATE 25mg/1mL injection |
|  | d52w. | FLUPHENAZINE DECANOATE 50mg/2mL injection |
|  | d52x. | FLUPHENAZINE DECANOATE 250mg/10mL injection |
|  | d53.. | *FLUPHENAZINE ENANTHATE |
|  | d531. | MODITEN ENANTHATE 25mg/1mL injection |
|  | d532. | FLUPHENAZINE ENANTHATE 25mg/1mL injection |
|  | d54.. | FLUSPIRILENE |
|  | d541. | *REDEPTIN 2mg/1mL injection |
|  | d542. | *REDEPTIN 6mg/3mL injection |
|  | d543. | *REDEPTIN 12mg/6mL injection |
|  | d544. | FLUSPIRILENE 2mg/1mL injection |
|  | d545. | FLUSPIRILENE 6mg/3mL injection |
|  | d546. | FLUSPIRILENE 12mg/6mL injection |
|  | d55.. | HALOPERIDOL DECANOATE |
|  | d551. | HALDOL DECANOATE 50mg/1mL injection |
|  | d552. | HALDOL DECANOATE 100mg/1mL injection |
|  | d553. | HALOPERIDOL 50mg/1mL injection |
|  | d554. | HALOPERIDOL 100mg/1mL injection |
|  | d56.. | PIPOTIAZINE PALMITATE |
|  | d561. | PIPORTIL DEPOT 50mg/1mL injection |
|  | d562. | PIPORTIL DEPOT 100mg/2mL injection |
|  | d563. | PIPOTIAZINE 50mg/1mL injection |
|  | d564. | PIPOTIAZINE 100mg/2mL injection |
|  | d57.. | ZUCLOPENTHIXOL DECANOATE |
|  | d571. | CLOPIXOL 200mg/1mL injection |
|  | d572. | *CLOPIXOL 2g/10mL injection |
|  | d573. | CLOPIXOL CONC. 500mg/1mL injection |
|  | d574. | CLOPIXOL ACUPHASE 50mg/1mL injection (oily) |
|  | d575. | CLOPIXOL ACUPHASE 100mg/2mL injection (oily) |
|  | d576. | ZUCLOPENTHIXOL DECANOATE 200mg/1mL injection |
|  | d577. | ZUCLOPENTHIXOL DECANOATE 50mg/1mL injection |
|  | d578. | ZUCLOPENTHIXOL DECANOATE 100mg/2mL injection |
|  | d57y. | ZUCLOPENTHIXOL DECANOATE 2g/10mL injection |
|  | d57z. | ZUCLOPENTHIXOL DECANOATE 500mg/1mL injection |
| **Atypical** | d4l.. | CLOZAPINE |
|  | d4l1. | CLOZAPINE 25mg tablets |
|  | d4l2. | CLOZAPINE 100mg tablets |
|  | d4l3. | CLOZARIL 25mg tablets x84CP |
|  | d4l4. | CLOZARIL 100mg tablets x84CP |
|  | d4l5. | CLOZARIL COMMUNITY PACK 25mg tablets x28CP |
|  | d4l6. | CLOZARIL COMMUNITY PACK 100mg tablets x28CP |
|  | d4l7. | DENZAPINE 25mg tablets |
|  | d4l8. | DENZAPINE 100mg tablets |
|  | d4l9. | ZAPONEX 25mg tablets |
|  | d4lA. | ZAPONEX 100mg tablets |
|  | d4lB. | DENZAPINE 50mg/mL oral suspension 100mL |
|  | d4lC. | CLOZAPINE 50mg/mL oral suspension |
|  | d4lD. | DENZAPINE 50mg tablets |
|  | d4lE. | CLOZAPINE 50mg tablets |
|  | d4lF. | DENZAPINE 200mg tablets |
|  | d4lG. | CLOZAPINE 200mg tablets |
|  | d4m.. | REMOXIPRIDE |
|  | d4m1. | REMOXIPRIDE 150mg m/r capsules |
|  | d4m2. | REMOXIPRIDE 300mg m/r capsules |
|  | d4m3. | *ROXIAM 150mg m/r capsules |
|  | d4m4. | *ROXIAM 300mg m/r capsules |
|  | d4p.. | RISPERIDONE |
|  | d4p1. | RISPERIDONE 1mg tablets |
|  | d4p2. | RISPERIDONE 2mg tablets |
|  | d4p3. | RISPERIDONE 3mg tablets |
|  | d4p4. | RISPERIDONE 4mg tablets |
|  | d4p5. | RISPERDAL 1mg tablets |
|  | d4p6. | RISPERDAL 2mg tablets |
|  | d4p7. | RISPERDAL 3mg tablets |
|  | d4p8. | RISPERDAL 4mg tablets |
|  | d4p9. | RISPERIDONE 1mg/mL liquid |
|  | d4pA. | RISPERDAL 1mg/mL liquid |
|  | d4pB. | RISPERIDONE 6mg tablets |
|  | d4pC. | RISPERDAL 6mg tablets |
|  | d4pD. | RISPERDAL 0.5mg tablets |
|  | d4pE. | RISPERDAL CONSTA 25mg powder+solvent for suspension for injection |
|  | d4pF. | RISPERDAL CONSTA 37.5mg powder+solvent for suspension for injection |
|  | d4pG. | RISPERDAL CONSTA 50mg powder+solvent for suspension for injection |
|  | d4pH. | RISPERIDONE 1mg oro-dispersible tablets |
|  | d4pJ. | RISPERIDONE 2mg oro-dispersible tablets |
|  | d4pK. | RISPERDAL QUICKLET 1mg oro-dispersible tablets |
|  | d4pL. | RISPERDAL QUICKLET 2mg oro-dispersible tablets |
|  | d4pM. | RISPERIDONE 0.5mg oro-dispersible tablets |
|  | d4pN. | RISPERDAL QUICKLET 0.5mg oro-dispersible tablets |
|  | d4pO. | RISPERDAL QUICKLET 3mg oro-dispersible tablets |
|  | d4pP. | RISPERDAL QUICKLET 4mg oro-dispersible tablets |
|  | d4pQ. | RISPERIDONE 3mg oro-dispersible tablets |
|  | d4pR. | RISPERIDONE 4mg oro-dispersible tablets |
|  | d4pw. | RISPERIDONE 50mg powder+solvent for suspension for injection |
|  | d4px. | RISPERIDONE 37.5mg powder+solvent for suspension for injection |
|  | d4py. | RISPERIDONE 25mg powder+solvent for suspension for injection |
|  | d4pz. | RISPERIDONE 0.5mg tablets |
|  | d4q.. | SERTINDOLE |
|  | d4q1. | SERTINDOLE 4mg tablets |
|  | d4q2. | SERTINDOLE 12mg tablets |
|  | d4q3. | SERTINDOLE 16mg tablets |
|  | d4q4. | SERTINDOLE 20mg tablets |
|  | d4q5. | SERDOLECT 4mg tablets |
|  | d4q6. | SERDOLECT 12mg tablets |
|  | d4q7. | SERDOLECT 16mg tablets |
|  | d4q8. | SERDOLECT 20mg tablets |
|  | d4r.. | OLANZAPINE |
|  | d4r1. | OLANZAPINE 5mg tablets |
|  | d4r2. | OLANZAPINE 7.5mg tablets |
|  | d4r3. | OLANZAPINE 10mg tablets |
|  | d4r4. | ZYPREXA 5mg tablets |
|  | d4r5. | ZYPREXA 7.5mg tablets |
|  | d4r6. | ZYPREXA 10mg tablets |
|  | d4r7. | OLANZAPINE 2.5mg tablets |
|  | d4r8. | ZYPREXA 2.5mg tablets |
|  | d4r9. | ZYPREXA VELOTAB 5mg dispersible tablets |
|  | d4rA. | ZYPREXA VELOTAB 10mg dispersible tablets |
|  | d4rB. | ZYPREXA 15mg tablets |
|  | d4rC. | ZYPREXA VELOTAB 15mg dispersible tablets |
|  | d4rD. | ZYPREXA 10mg injection (pdr for recon) |
|  | d4rE. | ZYPREXA VELOTAB 20mg dispersible tablets |
|  | d4rF. | ZYPREXA 20mg tablets |
|  | d4rG. | ZALASTA 2.5mg tablets |
|  | d4rH. | ZALASTA 5mg tablets |
|  | d4rI. | ZALASTA 7.5mg tablets |
|  | d4rJ. | ZALASTA 15mg tablets |
|  | d4rK. | ZALASTA 20mg tablets |
|  | d4rL. | ZALASTA 5mg dispersible tablets |
|  | d4rM. | ZALASTA 10mg dispersible tablets |
|  | d4rN. | ZALASTA 15mg dispersible tablets |
|  | d4rO. | ZALASTA 20mg dispersible tablets |
|  | d4rP. | ZALASTA 10mg tablets |
|  | d4rt. | OLANZAPINE 20mg tablets |
|  | d4ru. | OLANZAPINE 20mg dispersible tablets |
|  | d4rv. | OLANZAPINE 10mg injection (pdr for recon) |
|  | d4rw. | OLANZAPINE 15mg dispersible tablets |
|  | d4rx. | OLANZAPINE 15mg tablets |
|  | d4ry. | OLANZAPINE 5mg dispersible tablets |
|  | d4rz. | OLANZAPINE 10mg dispersible tablets |
|  | d4s.. | QUETIAPINE |
|  | d4s1. | QUETIAPINE 25mg tablets |
|  | d4s2. | QUETIAPINE 100mg tablets |
|  | d4s3. | QUETIAPINE 200mg tablets |
|  | d4s4. | QUETIAPINE 25mg+100mg tablets starter pack |
|  | d4s5. | SEROQUEL 25mg tablets |
|  | d4s6. | SEROQUEL 100mg tablets |
|  | d4s7. | SEROQUEL 200mg tablets |
|  | d4s8. | SEROQUEL 25mg+100mg tablets starter pack |
|  | d4s9. | SEROQUEL 150mg tablets |
|  | d4sA. | SEROQUEL 25mg+100mg+150mg tablets starter pack |
|  | d4sB. | SEROQUEL 300mg tablets |
|  | d4sC. | SEROQUEL XL 50mg m/r tablets |
|  | d4sD. | SEROQUEL XL 200mg m/r tablets |
|  | d4sE. | SEROQUEL XL 300mg m/r tablets |
|  | d4sF. | SEROQUEL XL 400mg m/r tablets |
|  | d4sG. | SEROQUEL XL 150mg m/r tablets |
|  | d4ss. | QUETIAPINE 150mg m/r tablets |
|  | d4st. | QUETIAPINE 400mg m/r tablets |
|  | d4su. | QUETIAPINE 300mg m/r tablets |
|  | d4sv. | QUETIAPINE 200mg m/r tablets |
|  | d4sw. | QUETIAPINE 50mg m/r tablets |
|  | d4sx. | QUETIAPINE 300mg tablets |
|  | d4sy. | QUETIAPINE 25mg+100mg+150mg tablets starter pack |
|  | d4sz. | QUETIAPINE 150mg tablets |
|  | d4t.. | AMISULPRIDE |
|  | d4t1. | AMISULPRIDE 50mg tablets |
|  | d4t2. | AMISULPRIDE 200mg tablets |
|  | d4t3. | SOLIAN 50mg tablets |
|  | d4t4. | SOLIAN 200mg tablets |
|  | d4t5. | SOLIAN 400mg tablets |
|  | d4t6. | SOLIAN 100mg/mL sugar free oral solution |
|  | d4t7. | SOLIAN 100mg tablets |
|  | d4tx. | AMISULPRIDE 100mg tablets |
|  | d4ty. | AMISULPRIDE 100mg/mL sugar free oral solution |
|  | d4tz. | AMISULPRIDE 400mg tablets |
|  | d4u.. | ZOTEPINE |
|  | d4u1. | *ZOTEPINE 25mg tablets |
|  | d4u2. | *ZOTEPINE 50mg tablets |
|  | d4u3. | *ZOTEPINE 100mg tablets |
|  | d4u4. | *ZOLEPTIL 25mg tablets |
|  | d4u5. | *ZOLEPTIL 50mg tablets |
|  | d4u6. | *ZOLEPTIL 100mg tablets |
|  | d4v.. | ARIPIPRAZOLE |
|  | d4v1. | ABILIFY 10mg tablets |
|  | d4v2. | ABILIFY 15mg tablets |
|  | d4v3. | ABILIFY 30mg tablets |
|  | d4v4. | ABILIFY 5mg tablets |
|  | d4v5. | ABILIFY 10mg oro-dispersible tablets |
|  | d4v6. | ABILIFY 15mg oro-dispersible tablets |
|  | d4v7. | ABILIFY 1mg/mL oral solution |
|  | d4v8. | ABILIFY 9.75mg/1.3mL solution for injection |
|  | d4vs. | ARIPIPRAZOLE 9.75mg/1.3mL solution for injection |
|  | d4vt. | ARIPIPRAZOLE 1mg/mL oral solution |
|  | d4vu. | ARIPIPRAZOLE 10mg oro-dispersible tablets |
|  | d4vv. | ARIPIPRAZOLE 15mg oro-dispersible tablets |
|  | d4vw. | ARIPIPRAZOLE 5mg tablets |
|  | d4vx. | ARIPIPRAZOLE 30mg tablets |
|  | d4vy. | ARIPIPRAZOLE 15mg tablets |
|  | d4vz. | ARIPIPRAZOLE 10mg tablets |
|  | d4w.. | PALIPERIDONE |
|  | d4w1. | INVEGA 3mg m/r tablets |
|  | d4w2. | INVEGA 6mg m/r tablets |
|  | d4w3. | INVEGA 9mg m/r tablets |
|  | d4w4. | *INVEGA 12mg m/r tablets |
|  | d4w5. | XEPLION 50mg suspension for injection prefilled syringe |
|  | d4w6. | XEPLION 75mg suspension for injection prefilled syringe |
|  | d4w7. | XEPLION 100mg suspension for injection prefilled syringe |
|  | d4w8. | XEPLION 150mg suspension for injection prefilled syringe |
|  | d4ws. | PALIPERIDONE 150mg suspension for injection pfs |
|  | d4wt. | PALIPERIDONE 100mg suspension for injection pfs |
|  | d4wu. | PALIPERIDONE 75mg suspension for injection prefilled syringe |
|  | d4wv. | PALIPERIDONE 50mg suspension for injection prefilled syringe |
|  | d4ww. | *PALIPERIDONE 12mg m/r tablets |
|  | d4wx. | PALIPERIDONE 9mg m/r tablets |
|  | d4wy. | PALIPERIDONE 6mg m/r tablets |
|  | d4wz. | PALIPERIDONE 3mg m/r tablets |
|  | d4x.. | ASENAPINE |
|  | d4x1. | SYCREST 5mg sublingual tablets |
|  | d4x2. | ASENAPINE 5mg sublingual tablets |
|  | d4x3. | SYCREST 10mg sublingual tablets |
|  | d4x4. | ASENAPINE 10mg sublingual tablets |
|  | d58.. | OLANZAPINE PAMOATE |
|  | d581. | ZYPADHERA 210mg powder+solvent for suspension for injection |
|  | d582. | ZYPADHERA 300mg powder+solvent for suspension for injection |
|  | d583. | ZYPADHERA 405mg powder+solvent for suspension for injection |
|  | d58x. | OLANZAPINE 405mg powder+solvent for suspension for injection |
|  | d58y. | OLANZAPINE 300mg powder+solvent for suspension for injection |
|  | d58z. | OLANZAPINE 210mg powder+solvent for suspension for injection |
| *Medication now discontinued | | |

# Table S21 List of Read codes used to identify prescription of hypnotics in SAIL

| **Read Code** | **Text Description** |
| --- | --- |
| d1... | HYPNOTICS |
| d11.. | CHLORAL HYDRATE |
| d111. | *CHLORAL 500mg/5mL mixture |
| d112. | CHLORAL PAEDIATRIC 200mg/5mL elixir |
| d113. | *NOCTEC 500mg capsules |
| d114. | CHLORAL HYDRATE 500mg capsules |
| d115. | WELLDORM 143mg/5mL elixir |
| d116. | WELLDORM tablets |
| d117. | CHLORAL HYDRATE 414mg tablets |
| d118. | CHLORAL HYDRATE 143mg/5mL elixir |
| d119. | CHLORAL HYDRATE 500mg/5mL syrup |
| d11A. | SOMNWELL 707mg tablets |
| d12.. | CLOMETHIAZOLE EDISYLATE [HYPNOTIC] |
| d121. | *HEMINEVRIN 192mg capsules |
| d122. | *HEMINEVRIN 250mg/5mL syrup |
| d123. | *HEMINEVRIN 8mg/mL infusion |
| d12v. | CLOMETHIAZOLE 192mg capsules |
| d12w. | *CLOMETHIAZOLE 250mg/5mL syrup |
| d12z. | CHLORMETHIAZOLE EDISYLATE 8mg/mL infusion |
| d13.. | *DICHLORALPHENAZONE |
| d131. | *WELLDORM 650mg tablets |
| d132. | *WELLDORM 225mg/5mL elixir |
| d13y. | DICHLORALPHENAZONE 650mg tablets |
| d13z. | DICHLORALPHENAZONE 225mg/5mL elixir |
| d14.. | *FLUNITRAZEPAM |
| d141. | *ROHYPNOL 1mg tablets |
| d14z. | *FLUNITRAZEPAM 1mg tablets |
| d15.. | FLURAZEPAM |
| d151. | DALMANE 15mg capsules |
| d152. | DALMANE 30mg capsules |
| d153. | *PAXANE 15mg capsules |
| d154. | *PAXANE 30mg capsules |
| d15y. | FLURAZEPAM 15mg capsules |
| d15z. | FLURAZEPAM 30mg capsules |
| d16.. | LOPRAZOLAM |
| d161. | LOPRAZOLAM 1mg tablets |
| d162. | *DORMONOCT 1mg tablets |
| d17.. | LORMETAZEPAM |
| d171. | LORMETAZEPAM 500micrograms tablets |
| d172. | LORMETAZEPAM 1mg tablets |
| d173. | NOCTAMID 500micrograms tablets |
| d174. | *NOCTAMID 1mg tablets |
| d18.. | NITRAZEPAM |
| d181. | *NITRAZEPAM 5mg capsules |
| d182. | NITRAZEPAM 5mg tablets |
| d183. | *NITRAZEPAM 10mg tablets |
| d184. | NITRAZEPAM 2.5mg/5mL mixture |
| d185. | *MOGADON 5mg capsules |
| d186. | MOGADON 5mg tablets |
| d187. | *NITRADOS 5mg tablets |
| d188. | *NOCTESED 5mg tablets |
| d189. | REMNOS 5mg tablets |
| d18a. | *REMNOS 10mg tablets |
| d18b. | *SOMNITE 5mg tablets |
| d18c. | SOMNITE 2.5mg/5mL mixture |
| d18d. | *SUREM 5mg capsules |
| d18e. | *UNISOMNIA 5mg tablets |
| d18f. | NITRAZEPAM 5mg/5mL suspension |
| d19.. | PROMETHAZINE HCL [HYPNOTIC] see section c8i.. |
| d1a.. | TEMAZEPAM [HYPNOTIC] |
| d1a1. | *TEMAZEPAM 10mg capsules |
| d1a2. | *TEMAZEPAM 15mg capsules |
| d1a3. | *TEMAZEPAM 20mg capsules |
| d1a4. | *TEMAZEPAM 30mg capsules |
| d1a5. | *TEMAZEPAM 10mg/5mL elixir |
| d1a6. | *NORMISON 10mg capsules |
| d1a7. | *NORMISON 20mg capsules |
| d1a8. | *TEMAZEPAM PLANPAK capsules |
| d1a9. | TEMAZEPAM 10mg tablets |
| d1aa. | TEMAZEPAM 20mg tablets |
| d1ab. | TEMAZEPAM GELTHIX 10mg capsules |
| d1ac. | TEMAZEPAM GELTHIX 20mg capsules |
| d1ad. | TEMAZEPAM GELTHIX 15mg capsules |
| d1ae. | TEMAZEPAM GELTHIX 30mg capsules |
| d1af. | TEMAZEPAM GEL FILLED 10mg capsules |
| d1ag. | TEMAZEPAM GEL FILLED 20mg capsules |
| d1ah. | TEMAZEPAM GEL FILLED 15mg capsules |
| d1ai. | TEMAZEPAM GEL FILLED 30mg capsules |
| d1aj. | TEMAZEPAM 10mg/sachet oral solution |
| d1ak. | TEMAZEPAM 20mg/sachet oral solution |
| d1al. | EUHYPNOS 10mg/sachet oral solution |
| d1am. | EUHYPNOS 20mg/sachet oral solution |
| d1an. | EUHYPNOS 10mg/5mL oral solution |
| d1ao. | TEMAZEPAM 10mg/5mL sugar free oral solution |
| d1b.. | *TRIAZOLAM |
| d1b1. | TRIAZOLAM 125microgram tablets |
| d1b2. | TRIAZOLAM 250microgram tablets |
| d1b3. | *HALCION 125microgram tablets |
| d1b4. | *HALCION 250microgram tablets |
| d1c.. | TRICLOFOS SODIUM |
| d1c1. | *TRICLOFOS 500mg/5mL liquid |
| d1d.. | ZOPICLONE |
| d1d1. | ZOPICLONE 7.5mg tablets |
| d1d2. | ZIMOVANE 7.5mg tablets |
| d1d3. | ZOPICLONE 3.75mg tablets |
| d1d4. | ZIMOVANE LS 3.75mg tablets |
| d1d5. | *ZILEZE 7.5 tablets |
| d1d6. | *ZILEZE 3.75 tablets |
| d1e.. | *GLUTETHIMIDE [no drugs here] |
| d1f.. | ZOLPIDEM |
| d1f1. | STILNOCT 5mg tablets |
| d1f2. | ZOLPIDEM TARTRATE 5mg tablets |
| d1f3. | ZOLPIDEM TARTRATE 10mg tablets |
| d1f4. | STILNOCT 10mg tablets |
| d1g.. | ZALEPLON |
| d1g1. | SONATA 5mg capsules |
| d1g2. | SONATA 10mg capsules |
| d1gy. | ZALEPLON 5mg capsules |
| d1gz. | ZALEPLON 10mg capsules |
| d1h.. | MELATONIN |
| d1h1. | CIRCADIN 2mg m/r tablets |
| d1hz. | MELATONIN 2mg m/r tablets |
| d1i.. | DEXMEDETOMIDINE |
| d1i1. | DEXDOR 200micrograms/2mL concentrate for soln for infusion |
| d1i2. | DEXMEDETOMIDINE 200micrograms/2mL conc for soln for inj |
| d1i3. | DEXDOR 400micrograms/4mL concentrate for soln for infusion |
| d1i4. | DEXMEDETOMIDINE 400micrograms/4mL conc for soln for inj |
| d1i5. | DEXDOR 1mg/10mL concentrate for solution for infusion |
| d1i6. | DEXMEDETOMIDINE 1mg/10mL concentrate for soln for injection |
| *Medication now discontinued | |

# Table S22 List of Read codes used to identify prescription of anxiolytics in SAIL

| **Read Code** | **Text Description** |
| --- | --- |
| d2... | ANXIOLYTICS |
| d21.. | DIAZEPAM [ANXIOLYTIC] |
| d211. | *DIAZEPAM 2mg capsules |
| d212. | *DIAZEPAM 5mg capsules |
| d213. | DIAZEPAM 2mg tablets |
| d214. | DIAZEPAM 5mg tablets |
| d215. | DIAZEPAM 10mg tablets |
| d216. | DIAZEPAM 2mg/5mL elixir |
| d217. | *ALUPRAM 2mg tablets |
| d218. | *ALUPRAM 5mg tablets |
| d219. | *ALUPRAM 10mg tablets |
| d21A. | DIAZEPAM 5mg/5mL oral solution |
| d21B. | *RIMAPAM 2mg tablets |
| d21C. | *RIMAPAM 5mg tablets |
| d21D. | *RIMAPAM 10mg tablets |
| d21E. | DIALAR 2mg/5mL syrup |
| d21F. | DIALAR 5mg/5mL syrup |
| d21G. | VALCLAIR 10mg suppositories |
| d21J. | DIAZEPAM 10mg/5mL suspension |
| d21a. | *ATENSINE 2mg tablets |
| d21b. | *ATENSINE 5mg tablets |
| d21c. | *ATENSINE 10mg tablets |
| d21d. | DIAZEMULS 10mg/2mL ampoules |
| d21e. | *EVACALM 2mg tablets |
| d21f. | *EVACALM 5mg tablets |
| d21g. | *SOLIS 2mg capsules |
| d21h. | *SOLIS 5mg capsules |
| d21i. | *STESOLID 10mg/2mL injection |
| d21j. | *STESOLID 20mg/4mL injection |
| d21k. | STESOLID 5mg rectal solution |
| d21l. | STESOLID 10mg rectal solution |
| d21m. | TENSIUM 2mg tablets |
| d21n. | TENSIUM 5mg tablets |
| d21o. | TENSIUM 10mg tablets |
| d21p. | *VALIUM 2mg capsules |
| d21q. | *VALIUM 5mg capsules |
| d21r. | *VALIUM 2mg tablets |
| d21s. | *VALIUM 5mg tablets |
| d21t. | *VALIUM 10mg tablets |
| d21u. | *VALIUM 2mg/5mL syrup |
| d21v. | VALIUM [ANXIOL] 10mg/2mL injection |
| d21w. | *VALIUM DUP DEL injection |
| d21x. | *VALIUM 5mg suppositories |
| d21y. | *VALIUM 10mg suppositories |
| d21z. | DIAZEPAM 10mg suppositories |
| d22.. | ALPRAZOLAM |
| d221. | XANAX 250micrograms tablets |
| d222. | XANAX 500micrograms tablets |
| d22y. | ALPRAZOLAM 250microgram tablets |
| d22z. | ALPRAZOLAM 500microgram tablets |
| d23.. | BROMAZEPAM |
| d231. | *LEXOTAN 1.5mg tablets |
| d232. | *LEXOTAN 3mg tablets |
| d23y. | *BROMAZEPAM 1.5mg tablets |
| d23z. | *BROMAZEPAM 3mg tablets |
| d24.. | CHLORDIAZEPOXIDE |
| d241. | CHLORDIAZEPOXIDE 5mg capsules |
| d242. | CHLORDIAZEPOXIDE 10mg capsules |
| d243. | CHLORDIAZEPOXIDE HYDROCHLORIDE 5mg tablets |
| d244. | CHLORDIAZEPOXIDE HYDROCHLORIDE 10mg tablets |
| d245. | CHLORDIAZEPOXIDE HYDROCHLORIDE 25mg tablets |
| d246. | *CHLORDIAZEPOXIDE 5mg tablets |
| d247. | *CHLORDIAZEPOXIDE 10mg tablets |
| d248. | *CHLORDIAZEPOXIDE 25mg tablets |
| d249. | LIBRIUM 5mg capsules |
| d24a. | LIBRIUM 10mg capsules |
| d24b. | *LIBRIUM 5mg tablets |
| d24c. | *LIBRIUM 10mg tablets |
| d24d. | *LIBRIUM 25mg tablets |
| d24e. | *LIBRIUM 100mg injection |
| d24f. | TROPIUM 5mg capsules |
| d24g. | *TROPIUM 10mg capsules |
| d24h. | TROPIUM 5mg tablets |
| d24i. | TROPIUM 10mg tablets |
| d24j. | *TROPIUM 25mg tablets |
| d25.. | CHLORMEZANONE |
| d251. | *TRANCOPAL 200mg tablets |
| d25z. | *CHLORMEZANONE 200mg tablets |
| d26.. | CLOBAZAM |
| d261. | *CLOBAZAM 10mg capsules |
| d262. | *FRISIUM 10mg capsules |
| d263. | CLOBAZAM 10mg tablets |
| d264. | FRISIUM 10mg tablets |
| d27.. | CLORAZEPATE DIPOTASSIUM |
| d271. | *TRANXENE 7.5mg capsules |
| d272. | *TRANXENE 15mg capsules |
| d27y. | CLORAZEPATE DIPOTASSIUM 7.5mg capsules |
| d27z. | CLORAZEPATE DIPOTASSIUM 15mg capsules |
| d28.. | HYDROXYZINE HCL [ANXIOLYTIC] |
| d281. | ATARAX 10mg tablets |
| d282. | ATARAX 25mg tablets |
| d283. | *ATARAX 10mg/5mL syrup |
| d284. | UCERAX 25mg tablets |
| d285. | UCERAX 10mg/5mL syrup |
| d28x. | HYDROXYZINE HCL 10mg tablets |
| d28y. | HYDROXYZINE HCL 25mg tablets |
| d28z. | HYDROXYZINE HCL 10mg/5mL syrup |
| d29.. | *KETAZOLAM |
| d291. | *ANXON 15mg capsules |
| d292. | *ANXON 30mg capsules |
| d29y. | *KETAZOLAM 15mg capsules |
| d29z. | *KETAZOLAM 30mg capsules |
| d2a.. | LORAZEPAM [ANXIOLYTIC] |
| d2a1. | LORAZEPAM 1mg tablets |
| d2a2. | LORAZEPAM 2.5mg tablets |
| d2a3. | *ALMAZINE 1mg tablets |
| d2a4. | *ALMAZINE 2.5mg tablets |
| d2a5. | *ATIVAN 1mg tablets |
| d2a6. | *ATIVAN 2.5mg tablets |
| d2a7. | ATIVAN 4mg/1mL injection |
| d2ax. | *ATIVAN 4mg/mL injection |
| d2az. | LORAZEPAM 4mg/1mL injection |
| d2b.. | *MEDAZEPAM |
| d2b1. | *NOBRIUM 5mg capsules |
| d2b2. | *NOBRIUM 10mg capsules |
| d2by. | *MEDAZEPAM 5mg capsules |
| d2bz. | *MEDAZEPAM 10mg capsules |
| d2c.. | MEPROBAMATE |
| d2c1. | *MEPROBAMATE 200mg tablets |
| d2c2. | MEPROBAMATE 400mg tablets |
| d2c3. | *EQUANIL 200mg tablets |
| d2c4. | *EQUANIL 400mg tablets |
| d2c5. | MEPRATE 400mg tablets |
| d2c6. | *TENAVOID tablets |
| d2d.. | OXAZEPAM |
| d2d1. | *OXAZEPAM 30mg capsules |
| d2d2. | OXAZEPAM 10mg tablets |
| d2d3. | OXAZEPAM 15mg tablets |
| d2d4. | OXAZEPAM 30mg tablets |
| d2d5. | *OXANID 10mg tablets |
| d2d6. | *OXANID 15mg tablets |
| d2d7. | *OXANID 30mg tablets |
| d2e.. | *PRAZEPAM |
| d2e1. | *CENTRAX 10mg tablets |
| d2ez. | *PRAZEPAM 10mg tablets |
| d2f.. | BUSPIRONE HYDROCHLORIDE |
| d2f1. | BUSPIRONE 5mg tablets |
| d2f2. | *BUSPAR 5mg tablets |
| d2f3. | *BUSPAR 5mg tablets x126 |
| d2f4. | *BUSPAR 10mg tablets |
| d2f5. | BUSPIRONE 10mg tablets |
| *Medication now discontinued | |

# Table S23 List of Read codes used to identify prescription of benzodiazepines in SAIL

| **Subtype** | **Read Code** | **Text Description** |
| --- | --- | --- |
| **Hypnotic** | d14.. | *FLUNITRAZEPAM |
|  | d141. | *ROHYPNOL 1mg tablets |
|  | d14z. | *FLUNITRAZEPAM 1mg tablets |
|  | d15.. | FLURAZEPAM |
|  | d151. | DALMANE 15mg capsules |
|  | d152. | DALMANE 30mg capsules |
|  | d153. | *PAXANE 15mg capsules |
|  | d154. | *PAXANE 30mg capsules |
|  | d15y. | FLURAZEPAM 15mg capsules |
|  | d15z. | FLURAZEPAM 30mg capsules |
|  | d16.. | LOPRAZOLAM |
|  | d161. | LOPRAZOLAM 1mg tablets |
|  | d162. | *DORMONOCT 1mg tablets |
|  | d17.. | LORMETAZEPAM |
|  | d171. | LORMETAZEPAM 500micrograms tablets |
|  | d172. | LORMETAZEPAM 1mg tablets |
|  | d173. | NOCTAMID 500micrograms tablets |
|  | d174. | *NOCTAMID 1mg tablets |
|  | d18.. | NITRAZEPAM |
|  | d181. | *NITRAZEPAM 5mg capsules |
|  | d182. | NITRAZEPAM 5mg tablets |
|  | d183. | *NITRAZEPAM 10mg tablets |
|  | d184. | NITRAZEPAM 2.5mg/5mL mixture |
|  | d185. | *MOGADON 5mg capsules |
|  | d186. | MOGADON 5mg tablets |
|  | d187. | *NITRADOS 5mg tablets |
|  | d188. | *NOCTESED 5mg tablets |
|  | d189. | REMNOS 5mg tablets |
|  | d18a. | *REMNOS 10mg tablets |
|  | d18b. | *SOMNITE 5mg tablets |
|  | d18c. | SOMNITE 2.5mg/5mL mixture |
|  | d18d. | *SUREM 5mg capsules |
|  | d18e. | *UNISOMNIA 5mg tablets |
|  | d18f. | NITRAZEPAM 5mg/5mL suspension |
|  | d1a.. | TEMAZEPAM [HYPNOTIC] |
|  | d1a1. | *TEMAZEPAM 10mg capsules |
|  | d1a2. | *TEMAZEPAM 15mg capsules |
|  | d1a3. | *TEMAZEPAM 20mg capsules |
|  | d1a4. | *TEMAZEPAM 30mg capsules |
|  | d1a5. | *TEMAZEPAM 10mg/5mL elixir |
|  | d1a6. | *NORMISON 10mg capsules |
|  | d1a7. | *NORMISON 20mg capsules |
|  | d1a8. | *TEMAZEPAM PLANPAK capsules |
|  | d1a9. | TEMAZEPAM 10mg tablets |
|  | d1aa. | TEMAZEPAM 20mg tablets |
|  | d1ab. | TEMAZEPAM GELTHIX 10mg capsules |
|  | d1ac. | TEMAZEPAM GELTHIX 20mg capsules |
|  | d1ad. | TEMAZEPAM GELTHIX 15mg capsules |
|  | d1ae. | TEMAZEPAM GELTHIX 30mg capsules |
|  | d1af. | TEMAZEPAM GEL FILLED 10mg capsules |
|  | d1ag. | TEMAZEPAM GEL FILLED 20mg capsules |
|  | d1ah. | TEMAZEPAM GEL FILLED 15mg capsules |
|  | d1ai. | TEMAZEPAM GEL FILLED 30mg capsules |
|  | d1aj. | TEMAZEPAM 10mg/sachet oral solution |
|  | d1ak. | TEMAZEPAM 20mg/sachet oral solution |
|  | d1al. | EUHYPNOS 10mg/sachet oral solution |
|  | d1am. | EUHYPNOS 20mg/sachet oral solution |
|  | d1an. | EUHYPNOS 10mg/5mL oral solution |
|  | d1ao. | TEMAZEPAM 10mg/5mL sugar free oral solution |
|  | d1b.. | *TRIAZOLAM |
|  | d1b1. | TRIAZOLAM 125microgram tablets |
|  | d1b2. | TRIAZOLAM 250microgram tablets |
|  | d1b3. | *HALCION 125microgram tablets |
|  | d1b4. | *HALCION 250microgram tablets |
| **Anxiolytics** | d21.. | DIAZEPAM [ANXIOLYTIC] |
|  | d211. | *DIAZEPAM 2mg capsules |
|  | d212. | *DIAZEPAM 5mg capsules |
|  | d213. | DIAZEPAM 2mg tablets |
|  | d214. | DIAZEPAM 5mg tablets |
|  | d215. | DIAZEPAM 10mg tablets |
|  | d216. | DIAZEPAM 2mg/5mL elixir |
|  | d217. | *ALUPRAM 2mg tablets |
|  | d218. | *ALUPRAM 5mg tablets |
|  | d219. | *ALUPRAM 10mg tablets |
|  | d21A. | DIAZEPAM 5mg/5mL oral solution |
|  | d21B. | *RIMAPAM 2mg tablets |
|  | d21C. | *RIMAPAM 5mg tablets |
|  | d21D. | *RIMAPAM 10mg tablets |
|  | d21E. | DIALAR 2mg/5mL syrup |
|  | d21F. | DIALAR 5mg/5mL syrup |
|  | d21G. | VALCLAIR 10mg suppositories |
|  | d21J. | DIAZEPAM 10mg/5mL suspension |
|  | d21a. | *ATENSINE 2mg tablets |
|  | d21b. | *ATENSINE 5mg tablets |
|  | d21c. | *ATENSINE 10mg tablets |
|  | d21d. | DIAZEMULS 10mg/2mL ampoules |
|  | d21e. | *EVACALM 2mg tablets |
|  | d21f. | *EVACALM 5mg tablets |
|  | d21g. | *SOLIS 2mg capsules |
|  | d21h. | *SOLIS 5mg capsules |
|  | d21i. | *STESOLID 10mg/2mL injection |
|  | d21j. | *STESOLID 20mg/4mL injection |
|  | d21k. | STESOLID 5mg rectal solution |
|  | d21l. | STESOLID 10mg rectal solution |
|  | d21m. | TENSIUM 2mg tablets |
|  | d21n. | TENSIUM 5mg tablets |
|  | d21o. | TENSIUM 10mg tablets |
|  | d21p. | *VALIUM 2mg capsules |
|  | d21q. | *VALIUM 5mg capsules |
|  | d21r. | *VALIUM 2mg tablets |
|  | d21s. | *VALIUM 5mg tablets |
|  | d21t. | *VALIUM 10mg tablets |
|  | d21u. | *VALIUM 2mg/5mL syrup |
|  | d21v. | VALIUM [ANXIOL] 10mg/2mL injection |
|  | d21w. | *VALIUM DUP DEL injection |
|  | d21x. | *VALIUM 5mg suppositories |
|  | d21y. | *VALIUM 10mg suppositories |
|  | d21z. | DIAZEPAM 10mg suppositories |
|  | d22.. | ALPRAZOLAM |
|  | d221. | XANAX 250micrograms tablets |
|  | d222. | XANAX 500micrograms tablets |
|  | d22y. | ALPRAZOLAM 250microgram tablets |
|  | d22z. | ALPRAZOLAM 500microgram tablets |
|  | d23.. | BROMAZEPAM |
|  | d231. | *LEXOTAN 1.5mg tablets |
|  | d232. | *LEXOTAN 3mg tablets |
|  | d23y. | *BROMAZEPAM 1.5mg tablets |
|  | d23z. | *BROMAZEPAM 3mg tablets |
|  | d24.. | CHLORDIAZEPOXIDE |
|  | d241. | CHLORDIAZEPOXIDE 5mg capsules |
|  | d242. | CHLORDIAZEPOXIDE 10mg capsules |
|  | d243. | CHLORDIAZEPOXIDE HYDROCHLORIDE 5mg tablets |
|  | d244. | CHLORDIAZEPOXIDE HYDROCHLORIDE 10mg tablets |
|  | d245. | CHLORDIAZEPOXIDE HYDROCHLORIDE 25mg tablets |
|  | d246. | *CHLORDIAZEPOXIDE 5mg tablets |
|  | d247. | *CHLORDIAZEPOXIDE 10mg tablets |
|  | d248. | *CHLORDIAZEPOXIDE 25mg tablets |
|  | d249. | LIBRIUM 5mg capsules |
|  | d24a. | LIBRIUM 10mg capsules |
|  | d24b. | *LIBRIUM 5mg tablets |
|  | d24c. | *LIBRIUM 10mg tablets |
|  | d24d. | *LIBRIUM 25mg tablets |
|  | d24e. | *LIBRIUM 100mg injection |
|  | d24f. | TROPIUM 5mg capsules |
|  | d24g. | *TROPIUM 10mg capsules |
|  | d24h. | TROPIUM 5mg tablets |
|  | d24i. | TROPIUM 10mg tablets |
|  | d24j. | *TROPIUM 25mg tablets |
|  | d26.. | CLOBAZAM |
|  | d261. | *CLOBAZAM 10mg capsules |
|  | d262. | *FRISIUM 10mg capsules |
|  | d263. | CLOBAZAM 10mg tablets |
|  | d264. | FRISIUM 10mg tablets |
|  | d27.. | CLORAZEPATE DIPOTASSIUM |
|  | d271. | *TRANXENE 7.5mg capsules |
|  | d272. | *TRANXENE 15mg capsules |
|  | d27y. | CLORAZEPATE DIPOTASSIUM 7.5mg capsules |
|  | d27z. | CLORAZEPATE DIPOTASSIUM 15mg capsules |
|  | d29.. | *KETAZOLAM |
|  | d291. | *ANXON 15mg capsules |
|  | d292. | *ANXON 30mg capsules |
|  | d29y. | *KETAZOLAM 15mg capsules |
|  | d29z. | *KETAZOLAM 30mg capsules |
|  | d2a.. | LORAZEPAM [ANXIOLYTIC] |
|  | d2a1. | LORAZEPAM 1mg tablets |
|  | d2a2. | LORAZEPAM 2.5mg tablets |
|  | d2a3. | *ALMAZINE 1mg tablets |
|  | d2a4. | *ALMAZINE 2.5mg tablets |
|  | d2a5. | *ATIVAN 1mg tablets |
|  | d2a6. | *ATIVAN 2.5mg tablets |
|  | d2a7. | ATIVAN 4mg/1mL injection |
|  | d2ax. | *ATIVAN 4mg/mL injection |
|  | d2az. | LORAZEPAM 4mg/1mL injection |
|  | d2b.. | *MEDAZEPAM |
|  | d2b1. | *NOBRIUM 5mg capsules |
|  | d2b2. | *NOBRIUM 10mg capsules |
|  | d2by. | *MEDAZEPAM 5mg capsules |
|  | d2bz. | *MEDAZEPAM 10mg capsules |
|  | d2d.. | OXAZEPAM |
|  | d2d1. | *OXAZEPAM 30mg capsules |
|  | d2d2. | OXAZEPAM 10mg tablets |
|  | d2d3. | OXAZEPAM 15mg tablets |
|  | d2d4. | OXAZEPAM 30mg tablets |
|  | d2d5. | *OXANID 10mg tablets |
|  | d2d6. | *OXANID 15mg tablets |
|  | d2d7. | *OXANID 30mg tablets |
|  | d2e.. | *PRAZEPAM |
|  | d2e1. | *CENTRAX 10mg tablets |
|  | d2ez. | *PRAZEPAM 10mg tablets |
| *Medication now discontinued | | |
